# Furan Oxidation-Cyclization to Oxazepines: Favoring *7‑exo-trig* over *6‑endo-trig* and *5‑exo-trig* Trajectories

**DOI:** 10.1021/acs.orglett.5c03626

**Published:** 2025-11-28

**Authors:** Fatma Albayrak Halac, Busra Nur Aydın Kandemir, Hasan Secen, Fraser F. Fleming, Ramazan Altundas

**Affiliations:** † Department of Chemistry, Faculty of Science, 52962 Gebze Technical University, Gebze, Kocaeli 41400, Türkiye; ‡ Department of Chemistry, Faculty of Science, 37503 Atatürk University, 25240 Erzurum, Türkiye; § Department of Chemistry, Drexel University, Philadelphia, Pennsylvania 19104, United States

## Abstract

The sequential oxidation-cyclization of hydroxyaminoalkylfurans to oxazepines is controlled by a regioselective *7-exo-trig* cyclization rather than the usually more favorable, *6-endo-trig* and *5-exo-trig* cyclizations. The key trajectory determinant is the generation of *Z*-dienones, which place the terminal substituents in close proximity. The versatile furan approach to oxazepines exploits an underdeveloped stereochemical feature allowing an efficient, regioselective, and robust route to variously substituted oxazepines ideal for bioactive discovery.

## Graphical abstract

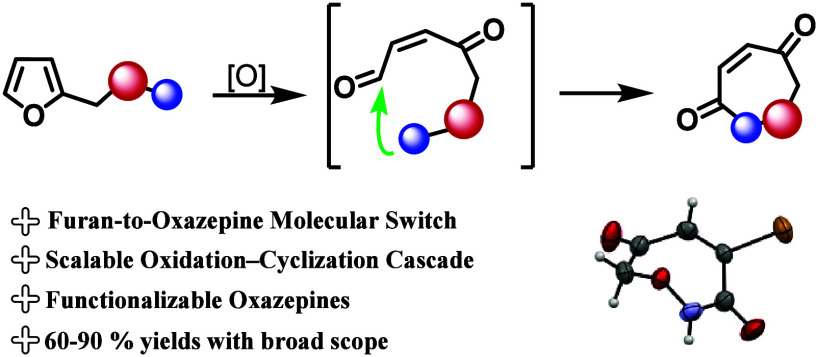

Seven-membered nitrogen heterocycles occupy a privileged position among the pharmaceuticals. The centrality of azapines is epitomized in the prescription of benzodiazepines to more than 30 million Americans each year.^1^ The high volume and numerous benzodiazepine analogs captures both the importance of these 7-membered heterocycles and the need to develop new scaffolds that more precisely target receptors while minimizing unwanted side effects.^2^

Among the emerging heterocyclic scaffolds are the oxazepanes, a non-natural yet bioactive scaffold incorporating a unique *N–O* connectivity (Figure 1).^3^ For example, the series of amide-substituted oxazepanes **1** inhibit calpains, enzymes that control cell signaling, muscle motility, and apoptosis,^4^ whereas the fused oxazepanes **2a** and structural analogs of the eudistomins (**2b**) inhibit the influenza virus.^5^ A complementary role for the oxazepenes is to provide a biocompatible scaffold for appending bioactive fragments to direct cell function as demonstrated for a library of analogs **3**, which used an FKBP binding domain to induce protein association (**3**).^6^

**Figure 1:**
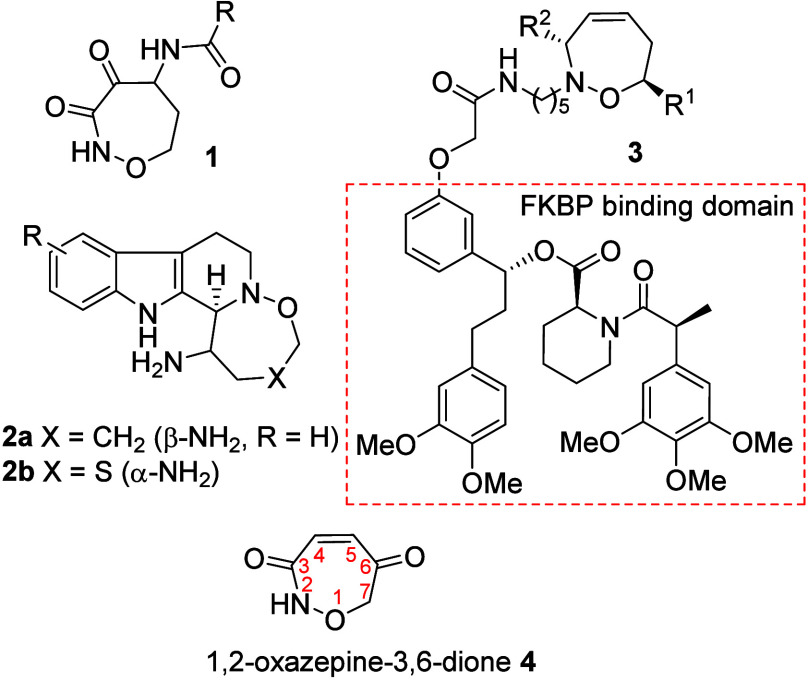
Bioactive oxazepine scaffolds.

Absent from the current oxazepanes are analogs allowing facile variation of substituents around the core at positions 2–7 to prepare diverse libraries of bioactive oxazepines.^3^ The four current approaches for accessing oxazepanes have not provided this diversity: ring-closing metathesis^6,7^ rearrangements,^5,8^ hydroxylamine cyclizations,^9^ and nitrone cycloadditions.^10^ The oxazepindione **4**, a currently unknown variant, appeared particularly attractive because of the potential for the controlled introduction of substituents through olefin and carbonyl additions.

A conceptually attractive entry to substituted oxazepines is via a sequenced oxidation-cyclization of substituted furans. Furans are nonobvious oxazepane precursors because the intermediate enediones have three different cyclization modes for which the requisite *7-endo-trig* would appear least likely to predominate (Scheme 1).^11^ However, buried within the enedione is a veiled stereochemical control element with the potential, largely unexplored, to promote cyclization to a 7-membered oxazepane: the olefin geometry.^12^ The *Z*-dienone geometry in **6** should position the terminal carbonyl proximal to the internal nucleophile favoring a *7-exo-trig* cyclization (solid orange arrow #1) in preference to the usual *6-endo-trig* (dashed black arrow #2) or *5-exo-trig* (dashed green arrow #3) cyclizations. Described below is the realization of this goal via the synthesis of several furans and their regioselective cyclization to a series of functionalized oxazepines **7**.

**Scheme 1:**
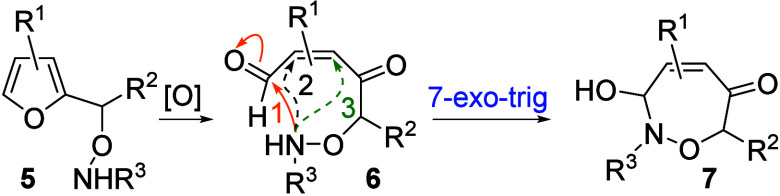
Furan Oxidation Route to Oxazepines

The feasibility of the furan-derived synthesis of oxazepines was explored by using inexpensive furfural (**8a**) as the precursor to the oxidation prototype. Reduction of furfural (**8a**) followed by PPh_3_-DEAD activation and in situ displacement with *N*-hydroxyphthalimide (NHPI) efficiently provided phthalimide **9a**. Subsequent release of the phthalimide with hydrazine, followed by treatment with Boc anhydride, afforded **5a** with the *N–O* functionality suitably protected for furan oxidation (Scheme 2).

**Scheme 2:**
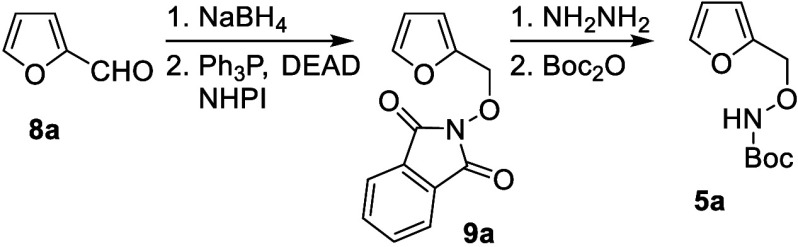
Synthesis of Furylhydroxylamine Prototype **5a**

Two different reagents were evaluated for the oxidation of furan **5a** because often a strong reagent sensitivity is observed.^13^ Comparative oxidations of **5a** with NBS in water against *m*-CPBA in CH_2_Cl_2_ indicated that NBS afforded a rather complex reaction mixture whereas the use of *m*-CPBA (1.5 equiv, 0–25 °C) afforded essentially only one component. Furthermore, the presence of diagnostic ^1^H NMR signals at 6.40 and 6.20 ppm and IR absorption at 3400 cm^–1^ supported the formation of the oxazepine **7a** from a *7-exo-trig* cyclization of **6a**. Repeating the *m*-CPBA oxidation-cyclization sequence on a 1 g scale followed by chromate oxidation of **7a** provided oxazepindione **10a** in 70% yield as a beautiful white solid (Scheme 3).

**Scheme 3:**
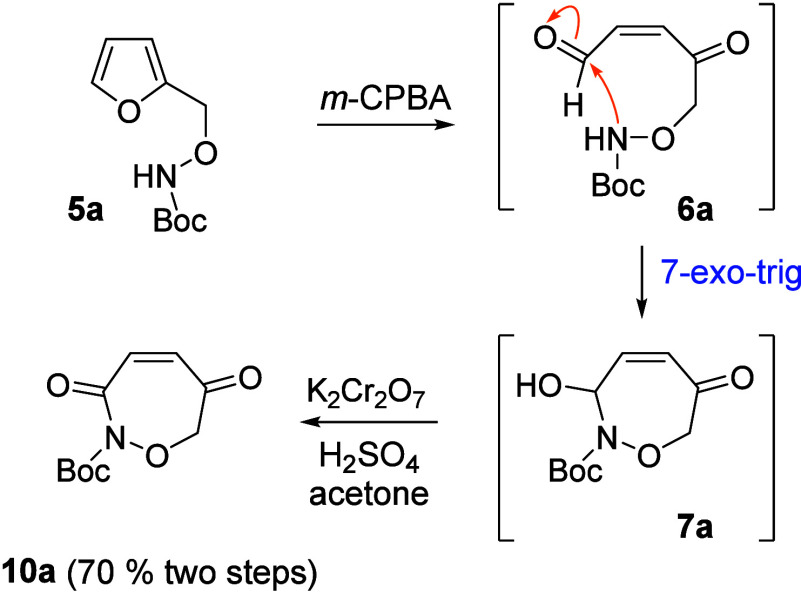
Oxidation-Cyclization to Oxazepines **7a** and **10a**

Having established that the key cyclization proceeds selectively and efficiently to the oxazepine, we prepared a series of substrates to evaluate the reaction scope. The requisite furanmethanols **11** were readily prepared (details in SI) and then subjected to the hydroxyphthalimide displacement-hydrazinolysis to provide the *o*-furylmethylhydroxylamines **12** (Table 1) that were directly treated with Boc anhydride to provide furans **5** for oxidation. The synthesis of furan methanol **11** and the two-step conversion to **5** are efficient and robust and provide a range of substituted furans for oxidative cyclization.

**Table 1:**
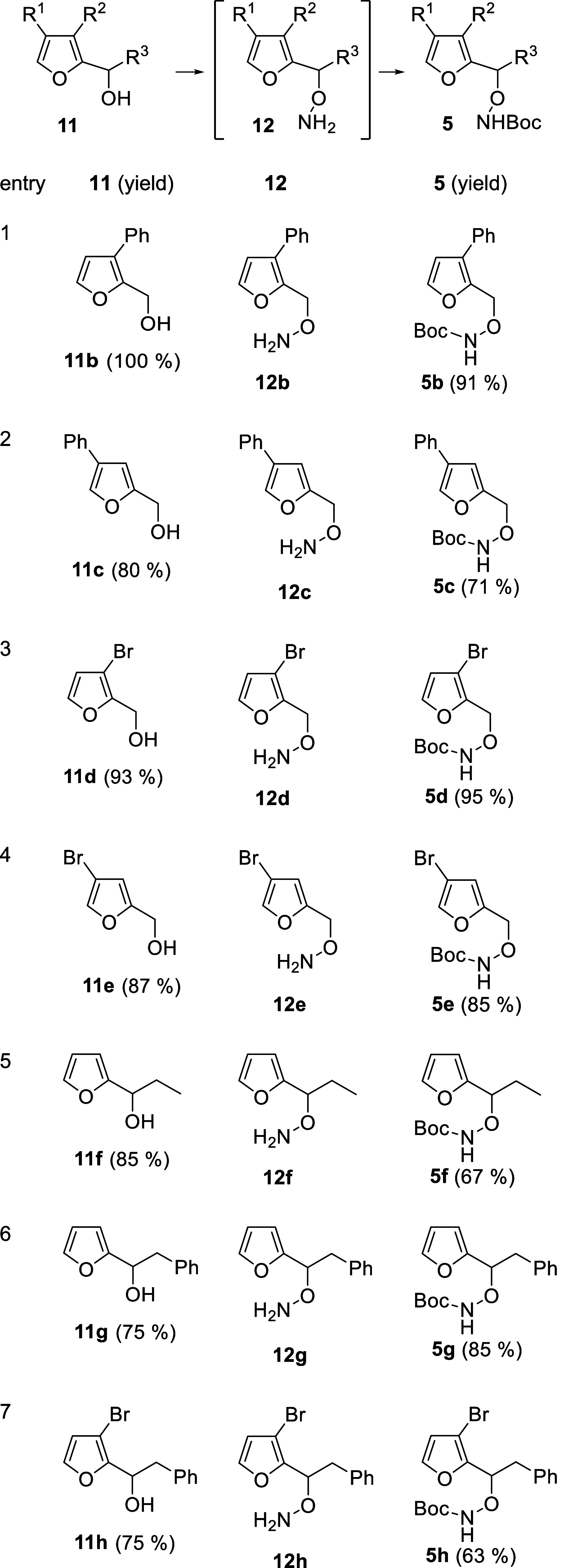
Synthesis of Boc-Protected Hydroxylamines **5** ^a^

a The crude was used in the next step without any further purification; yield was not calculated.

The scope of furan oxidation-cyclization was explored by applying the *m*-CPBA oxidation procedure to a series of Boc-protected hydroxylamines **5** (Scheme 4). Simply exposing the 3-phenyl- or 4-phenylhydroxylamines **5b** and **5c** to *m*-CPBA for 20 min smoothly afforded oxazepines **7b** and **7c** in 57% and 71% yield, respectively.^14^ Analogous oxidations of the 3-bromo- or 4-bromohydroxylamines **5d** and **5e** similarly afforded the oxazepines **7d** and **7e**; in the latter, case exposure to K_2_CrO_7_/H_2_SO_4_ afforded oxazepindione **10e** in which the Boc protecting group was efficiently removed. X-ray analysis confirmed the oxazepine structures (Scheme 4). Applying the same stepwise oxidation sequence to the ethyl-substituted hydroxylamine **5f** effectively installed the ethyl group at C-7 of oxazepine **7f** that was oxidized to oxazepindione **10f**. Telescoping both oxidation steps allowed the direct conversion of hydroxylamines **5g** and **5h** into oxazepindiones **10g** and **10h**.

**Scheme 4:**
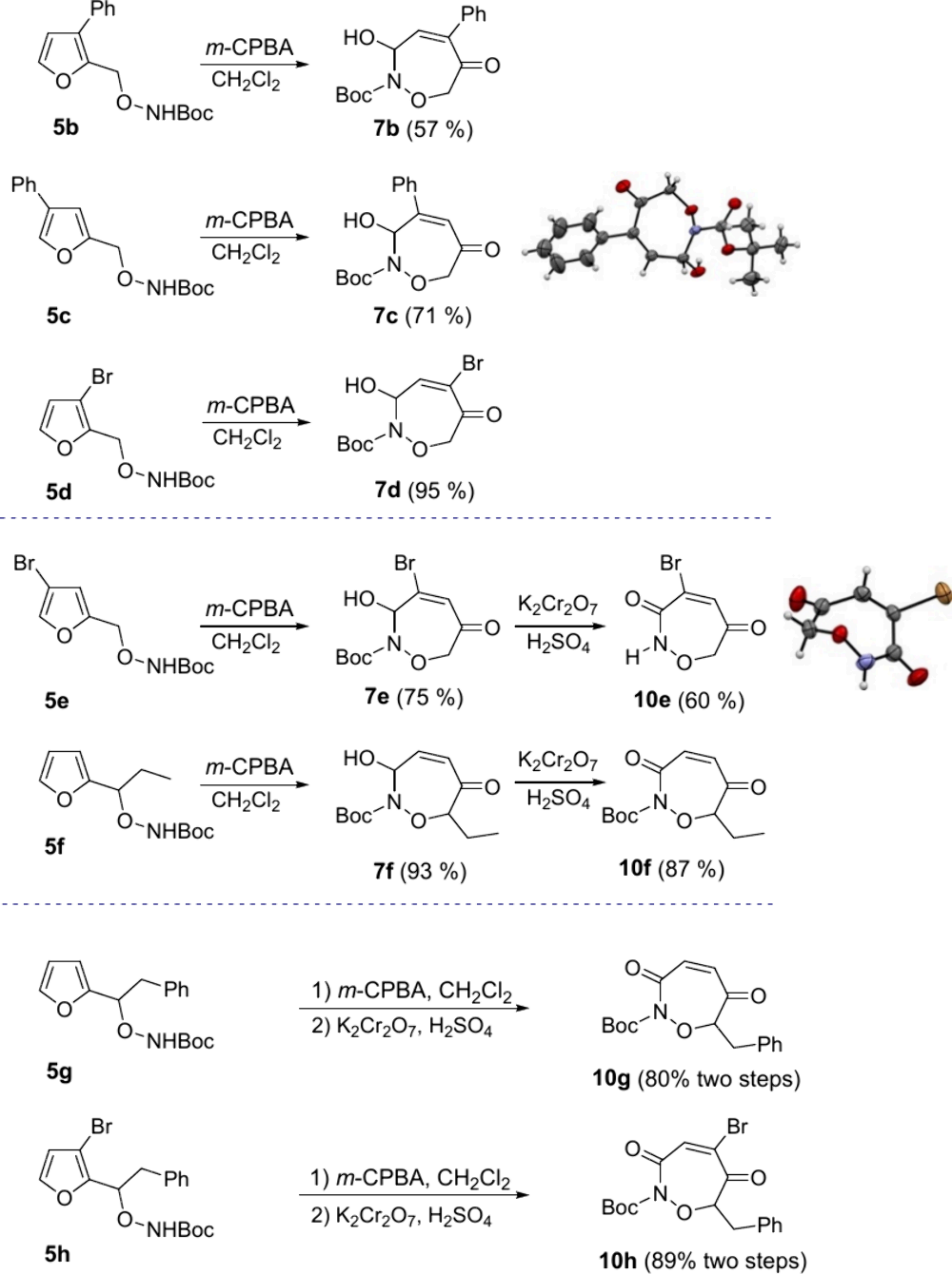
Furan Oxidation-Cyclization to Oxazepines

Having successfully synthesized a series of substituted oxazepines, we explored the potential for further functionalization. Treating **10e** with *tert*-butyl acrylate under typical Heck coupling conditions followed by chromatographic separation very efficiently afforded **14e** (Scheme 5). The excellent yield demonstrates the viability of elaborating these benign oxazepine scaffolds.

**Scheme 5:**
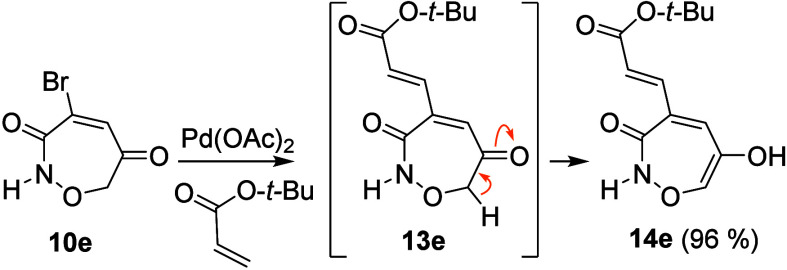
Representative Oxazepine Functionalization

The oxidative cyclization of a series of hydroxylaminoalkylfurans efficiently afforded a series of richly substituted oxazepines and oxazepindiones. The regioselective cyclization exploits a rare feature of the intermediate dienones generated by furan oxidation: the *Z*-olefin geometry predisposes cyclization with an appended nucleophile to 7-membered oxazepines rather than to 5- or 6-membered heterocycles. Oxidation of the oxazepines smoothly affords 1,2-oxepin-3,6-diones cleanly, allowing the processes to be telescoped into an efficient one-pot process. Collectively, the furan oxidative cyclization strategy provides rapid access to a range of variously substituted oxazepines that are readily manipulated by reactions such as Heck olefination. The rapid synthesis of diverse oxazepines, the fundamental insight into regioselective *7-exo-trig* cyclizations, and biological potential of derivatives provide a versatile platform for developing these novel chemical entities that should be attractive for applications in synthetic organic chemistry, drug discovery, and materials science.

## Supplementary Material





## Data Availability

The data underlying this study are available in the published article and its Supporting Information.

## Notes

Experimental details, NMR data (copies of ^1^H and ^13^C NMR spectra), and X-ray crystallographic data (PDF)
FAIR data, including the primary NMR FID files, for compounds **5a**–**5h**, **7a**–**7f**, **8b**–**8d**, **9a**–**9h**, **10a**–**10h**, **11a**–**11g**, **12b**–**12f**, and **14e** (ZIP)
