# Supplementary material for: Furan Oxidation-Cyclization to Oxazepines: Favoring 7‑exo-trig over 6‑endo-trig and 5‑exo-trig Trajectories
Source: Org Lett. 2025 Nov 28;27(49):13408–11. doi: 10.1021/acs.orglett.5c03626 (PMC12706832; doi:10.1021/acs.orglett.5c03626)
Supplement: Supplementary file 1 [file ol5c03626_si_001.pdf]

SUPPORTING INFORMATION

FURAN OXIDATION-CYCLIZATION TO OXAZEPINES: FAVORING *7-EXO-TRIG* OVER *6-ENDO-TRIG* AND *5-EXO-TRIG* TRAJECTORIES

Fatma Albayrak Halac,[a] Busra Nur Aydın Kandemir,[a,b] Hasan Secen,[b] Fraser F. Fleming,[c] Ramazan Altundas[a]*

[a]Department of Chemistry, Faculty of Science, Gebze Technical University, Gebze, 41400, Kocaeli, Türkiye; [b]Department of Chemistry, Faculty of Science, Atatürk University, 25240, Erzurum, Türkiye; [c]Department of Chemistry, Drexel University, Philadelphia, 19104, Pennsylvania, United States

Email: raltundas@gtu.edu.tr

Table of Contents

1. General Information 3
2. Experimental Procedures 3
  2.1. General Procedure for The Preparation of Hydroxyalkylfurans. 3
    2.1.1. General Procedure for Lithiation of Furan Derivatives. 3
    2.1.2. General Procedure for Lithiation of 3-Bromofuran Derivatives. 3
    2.1.3. General Procedure for Reduction of Furaldehyde Derivatives. 3
  2.2. General Procedure for The Suzuki Coupling Reaction. 4
  2.3. General Procedure for The Heck Coupling Reaction. 4
  2.4. General Procedure for The Preparation of Furylalkylhydroxyphthalimide. 4
  2.5. General Procedure for The Preparation of Furylalkylhydroxylamine. 4
  2.6. General Procedure for The Preparation of Boc-Protected Furylalkylhydroxylamine. 4
  2.7. General Procedure for Furan Oxidation Using *m*CPBA. 4
    2.8. General Procedure for Furan Oxidation Using NBS. 4
  2.9. General Procedure for The Jones Oxidation. 4
3. Characterization of Compounds 5
  furan-2-ylmethanol (11a) 5
  2-(furan-2-ylmethoxy)isoindoline-1,3-dione (9a) 6
  O-(furan-2-ylmethyl)hydroxylamine (12a) 6
  tert-butyl (furan-2-ylmethoxy)carbamate (5a) 6
  tert-butyl 3,6-dioxo-6,7-dihydro-1,2-oxazepine-2(3H)-carboxylate (10a) 6
  3-phenylfuran-2-carbaldehyde (8b) 7

(3-phenylfuran-2-yl)methanol (11b) 7
2-((3-phenylfuran-2-yl)methoxy)isoindoline-1,3-dione (9b) 7
O-((3-phenylfuran-2-yl)methyl)hydroxylamine (12b) 7
tert-butyl ((3-phenylfuran-2-yl)methoxy)carbamate (5b) 8
tert-butyl 3-hydroxy-6-oxo-5-phenyl-6,7-dihydro-1,2-oxazepine-2(*3H*)-carboxylate (7b) 8
4-phenylfuran-2-carbaldehyde (8c) 8
(4-phenylfuran-2-yl)methanol (11c) 8
2-((4-phenylfuran-2-yl)methoxy)isoindoline-1,3-dione (9c) 9
O-((4-phenylfuran-2-yl)methyl)hydroxylamine (12c) 9
tert-butyl ((4-phenylfuran-2-yl)methoxy)carbamate (5c) 9
tert-butyl 3-hydroxy-6-oxo-4-phenyl-6,7-dihydro-1,2-oxazepine-2(*3H*)-carboxylate (7c) 10
3-bromofuran-2-carbaldehyde (8d) 10
(3-bromofuran-2-yl)methanol (11d) 10
2-((3-bromofuran-2-yl)methoxy)isoindoline-1,3-dione (9d) 10
O-((3-bromofuran-2-yl)methyl)hydroxylamine (12d) 11
tert-butyl ((3-bromofuran-2-yl)methoxy)carbamate (5d) 11
tert-butyl 5-bromo-3-hydroxy-6-oxo-6,7-dihydro-1,2-oxazepine-2(*3H*)-carboxylate (7d) 11
5-bromo-3-hydroxy-2,3-dihydro-1,2-oxazepin-6(*7H*)-one (7d) 11
(4-bromofuran-2-yl)methanol (11e) 12
2-((4-bromofuran-2-yl)methoxy)isoindoline-1,3-dione (9e) 12
O-((4-bromofuran-2-yl)methyl)hydroxylamine (12e) 12
tert-butyl ((4-bromofuran-2-yl)methoxy)carbamate (5e) 12
tert-butyl 4-bromo-3-hydroxy-6-oxo-6,7-dihydro-1,2-oxazepine-2(*3H*)-carboxylate (7e) 13
4-bromo-1,2-oxazepine-3,6(*2H,7H*)-dione (10e) 13
tert-butyl (E)-3-(6-hydroxy-3-oxo-2,3-dihydro-1,2-oxazepin-4-yl)acrylate (14e) 13
1-(furan-2-yl)propan-1-ol (11f) 14
2-(1-(furan-2-yl)propoxy)isoindoline-1,3-dione (9f) 14
O-(1-(furan-2-yl)propyl)hydroxylamine (12f) 14
tert-butyl (1-(furan-2-yl)propoxy)carbamate (5f) 14
tert-butyl 7-ethyl-3-hydroxy-6-oxo-6,7-dihydro-1,2-oxazepine-2(*3H*)-carboxylate (7f) 15
tert-butyl 7-ethyl-3,6-dioxo-6,7-dihydro-1,2-oxazepine-2(*3H*)-carboxylate (10f) 15
1-(furan-2-yl)-2-phenylethanol (11g) 15
2-(1-(furan-2-yl)-2-phenylethoxy)isoindoline-1,3-dione (9g) 15
O-(1-(furan-2-yl)-2-phenylethyl)hydroxylamine (12g) 16
tert-butyl (1-(furan-2-yl)-2-phenylethoxy)carbamate (5g) 16
tert-butyl 7-benzyl-3,6-dioxo-6,7-dihydro-1,2-oxazepine-2(*3H*)-carboxylate (10g) 16
1-(3-bromofuran-2-yl)-2-phenylethanol (11h) 16

2-(1-(3-bromofuran-2-yl)-2-phenylethoxy)isoindoline-1,3-dione (9h) 17
O-(1-(3-bromofuran-2-yl)-2-phenylethyl)hydroxylamine (12h) 17
tert-butyl (1-(3-bromofuran-2-yl)-2-phenylethoxy)carbamate (5h) 17
tert-butyl 7-benzyl-5-bromo-3,6-dioxo-6,7-dihydro-1,2-oxazepine-2(*3H*)-carboxylate (10h) 17
4. X-ray Crystallographic Data 18
X-ray crystallographic data and structure refinement 18
5. ^{1}H and ^{13}C NMR Spectra 20
6. References 65

1. GENERAL INFORMATION

All reactions were carried out using oven dried glassware. All reagents and solvents were purchased from Sigma-Aldrich, Alfa Aeser and Merck depending on their availability. Anhydrous solvents were distilled over appropriate drying agents prior to use. All reactions were monitored by TLC (Merck 105715 Silica Gel 60 F254 25 TLC Plates) with detection by UV light (254 nm). PMA (phosphomolybdic acid) and CAM (cerium ammonium molybdate) were used as the TLC stain. The crude reaction mixtures were purified by column chromatography using Silica gel 60 (0.040-0.063 mm, 230-400 mesh). FTIR spectra were recorded on a Perkin Elmer Spectrum 100 spectrometer. ^{1}H and ^{13}C NMR spectra were recorded on a Varian 500 MHz spectrometer and Bruker 500 MHz spectrometer in $CDCl_3$ or DMSO-d6 solutions. Chemical shifts (δ) are quoted in ppm and referenced to TMS as internal standard. Coupling constants (J) are quoted in Hz. Multiplicity was recorded for ^{1}H NMR as follows *s* = singlet, *brs* = broad singlet, *d* = doublet, *t* = triplet, *q* = quartet, *m* = multiplet, *p* = pentet, *dd* = doublet of doublets. HRMS analyses were performed on Agilent Technologies 6200 series TOF/6500 series. MALDI-TOF mass spectrometry analyses were carried out on Bruker microflex LT MALDI-TOF MS spectrometer. The matrix is indicated in brackets for each compound. LC-MS analyses were performed using a Thermo Scientific TSQ Quantis Plus Mass Spectrometer. The X-ray single crystal diffraction data were performed using a Bruker Smart Apex II Quazar single crystal X-ray diffractometer.

2. EXPERIMENTAL PROCEDURES

2.1. General Procedure for The Preparation of Hydroxyalkylfurans.

2.1.1. General Procedure for Lithiation of Furan Derivatives. To a solution of furan (73.5 mmol, 1 eq) in dry THF (150 mL, 0.5 M) was added *n*-BuLi (2.5 M in Hexanes, 73.5 mmol, 1 eq) at -78 °C in an acetone/liquid nitrogen bath under nitrogen atmosphere. The resulting mixture was stirred while warming to -10 °C. It was then cooled to -78°C and treated with aldehyde derivatives (73.5 mmol, 1 eq). The reaction was stirred for overnight and was quenched with saturated NH_4Cl solution. The reaction mixture was extracted with EtOAc (3x). The combined organic layers were dried over Na_2SO_4, filtered and concentrated *in vacuo*. The residues were purified by column chromatography (EtOAc/*n*-Hexane) to afford the corresponding product.

2.1.2. General Procedure for Lithiation of 3-Bromofuran Derivatives. To a solution of Diisopropylamine (37.4 mmol, 1.1 eq) in dry THF (120 mL, 0.3 M) was added *n*-BuLi (2.5 M in Hexanes, 34 mmol, 1 eq) at -78 °C in an acetone/liquid nitrogen bath under nitrogen atmosphere. After stirring for 10 minutes, 3-bromofuran (34 mmol, 1 eq) was added to the resulting mixture. The reaction temperature was maintained between -78°C and -60°C while stirring for 1.5 hours. The reaction was then treated with aldehyde derivative or DMF (34 mmol, 1 eq). The reaction was stirred for overnight and was quenched with saturated NH_4Cl solution. The reaction mixture was extracted with EtOAc (3x). The combined organic layers were dried over Na_2SO_4, filtered and concentrated *in vacuo*. The residues were purified by column chromatography (EtOAc/*n*-Hexane) to afford the corresponding product.

2.1.3. General Procedure for Reduction of Furaldehyde Derivatives. Furaldehyde derivative filtered through silicagel pad with *n*-Hexane, if necessary. After evaporation of solvent, furfural derivatives (8.6 mmol, 1 eq) dissolved in methanol (43 mL, 0.2 M). Reaction flask was placed in an ice bath, and $NaBH_4$ (13 mmol, 1.5 eq) was added portionwise at 0°C. Reaction mixture was stirred at same temperature for 30 min to 2h. After completion confirmed by TLC, reaction mixture was quenched with saturated NH_4Cl solution, and most of the solvent was evaporated. The mixture was extracted with EtOAc (3x). The combined organic layers were dried over Na_2SO_4, filtered and concentrated

in vacuo. The residues were purified by column chromatography (EtOAc/*n*-Hexane) to afford the corresponding product.

2.2. General Procedure for The Suzuki Coupling Reaction. To a solution of the starting material (8.6 mmol, 1 eq) in 1,4-dioxane (43 mL, 0.2 M), $Pd(PPh_3)_4$ (0.17 mmol, 0.02 eq), phenylboronic acid (10.3 mmol, 1.2 eq), and K_2CO_3 (21.5 mmol, 2.5 eq) were added at room temperature. The reaction mixture was refluxed in an oil bath for 2 days. Upon the completion of the reaction judged by TLC, the reaction mixture was filtered through filter paper and transferred to a separatory funnel. The mixture was diluted with EtOAc and extracted with brine. The aqueous layer was extracted with EtOAc (3x). The combined organic layers were dried over Na_2SO_4, filtered and concentrated *in vacuo*. The crude was purified by column chromatography (EtOAc/*n*-Hexane) to afford the corresponding product.

2.3. General Procedure for The Heck Coupling Reaction. To a solution of $Pd(OAc)_2$ (0.0007 mmol, 0.0015 eq) and PPh_3 (0.0006 mmol, 0.0012 eq) in acetonitrile (2.4 mL, 0.2 M) were added Et_3N (0.005 mmol, 0.01 eq), the starting material (0.48 mmol, 1 eq), and *tert*-butyl acrylate (1.44 mmol, 3 eq). The mixture was sealed in a tube and stirred at 100°C in an oil bath for overnight. After cooling to room temperature, the reaction completion was confirmed by TLC. The mixture was then diluted with water and extracted with EtOAc (3x). The combined organic layers were dried over Na_2SO_4, filtered and concentrated *in vacuo*. The crude product was purified by column chromatography (EtOAc/*n*-Hexane) to afford the corresponding product.

2.4. General Procedure for The Preparation of Furylalkylhydroxyphthalimide. To a solution of PPh_3 (8.25 mmol, 1.1 eq) and *N*-hydroxyphthalimide (8.25 mmol, 1.1 eq) in a portion of dry THF was added DEAD (40 wt % in toluene) (8.25 mmol, 1.1 eq) at 0 °C under nitrogen atmosphere in an ice bath. A solution of hyroxyalkylfuran (7.5 mmol, 1 eq) in a separate portion of dry THF was then added. The total reaction volume was adjusted to give a concentration of 0.2 M (37 mL) with respect to the hydroxyalkylfuran, and the reaction mixture was allowed to warm to room temperature. Upon the completion of the reaction judged by TLC, it was quenched with brine. The organic layer was separated, and the aqueous layer was extracted with EtOAc (3x). The combined organic layers were dried over Na_2SO_4, filtered and concentrated *in vacuo*. The crude was purified by column chromatography (EtOAc/*n*-Hexane) to afford the furyl hydroxyphthalimide.

2.5. General Procedure for The Preparation of Furylalkylhydroxylamine. To solution of furyl hydroxyphthalimide (4.6 mmol, 1 eq) in dry DCM (23 mL, 0.2 M) was added hydrazine hydrate (23 mmol, 5 eq) under nitrogen atmosphere at rt. The reaction mixture was stirred for overnight. Upon the completion of the reaction judged by TLC and the mixture was filtered and concentrated *in vacuo*. The crude product was not fully characterized and was used directly in the next step.

2.6. General Procedure for The Preparation of Boc-Protected Furylalkylhydroxylamine. To a solution of crude furylalkylhydroxylamine (4.4 mmol, 1 eq) in a portion of dry THF was added Et_3N (6.6 mmol, 1.5 eq) at 0 °C in an ice bath under nitrogen atmosphere. Boc_2O (4.84 mmol, 1.1 eq) in a separate portion dry THF was then added dropwise. The total reaction volume was adjusted to give a concentration of 0.2 M (15+7 mL) with respect to the furylalkylhydroxylamine and the reaction mixture was stirred for overnight while allowed to warm to rt. Upon completion judged by TLC, the reaction was quenched with saturated NH_4Cl solution and extracted with EtOAc (3x). The combined organic layers were dried over Na_2SO_4, filtered and concentrated *in vacuo*. The residue was purified by silicagel column chromatography (EtOAc/*n*-Hexane) to afford Boc-protected product.

2.7. General Procedure for Furan Oxidation Using *m*CPBA. To a solution of Boc-protected furylalkylhydroxylamine (1.4 mmol, 1 eq) in DCM (7 mL, 0.2 M) was added *m*CPBA (2.1 mmol, 1.5 eq) at rt. The resulting mixture was stirred overnight. Upon the completion of the reaction judged by TLC, it was quenched with saturated $NaHSO_3$ solution and extracted with DCM (3x). The combined organic layers were washed with saturated $NaHCO_3$ solution (2x). The organic layer was dried over Na_2SO_4, filtered and concentrated *in vacuo*. The residue was used in next step without further purification.

2.8. General Procedure for Furan Oxidation Using NBS. Boc-protected furylalkylhydroxylamine (0.2 mmol, 1 eq) was dissolved in THF (1.2 mL): H_2O (0.6 mL) and cooled to 0 °C in an ice bath. The total reaction volume was adjusted to achieve a concentration of 0.1 M with respect to the furylalkylhydroxylamine. $NaHCO_3$ (0.5 mmol, 2.5 eq), NaOAc (0.3 mmol, 1.5 eq) and NBS (0.3 mmol, 1.5 eq) were added to the mixture sequentially. The resulting mixture was stirred for 20 min. Upon the completion of the reaction judged by TLC, it was quenched with saturated $NaHCO_3$ solution and extracted with EtOAc (3x). The organic layer was dried over Na_2SO_4, filtered and concentrated *in vacuo*. The residue was used in next step without further purification.

2.9. General Procedure for The Jones Oxidation. The crude (1.2 mmol, 1 eq) from *general procedure 2.7.* or *general procedure 2.8.* was dissolved in acetone (12 mL, 0.1 M), and Jones reagent (1.3 M, 1.3 mmol, 1.1 eq) was added dropwise at rt. Upon the completion of the reaction judged by TLC, the reaction was quenched with H_2O and concentrated in vacuo. The residue was extracted with DCM (3x). The combined organic layers were dried over Na_2SO_4, filtered and

concentrated *in vacuo*. The residue was purified by silicagel column chromatography (EtOAc/*n*-Hexane) to afford corresponding 1,2-oxazepine-3,6-dion.

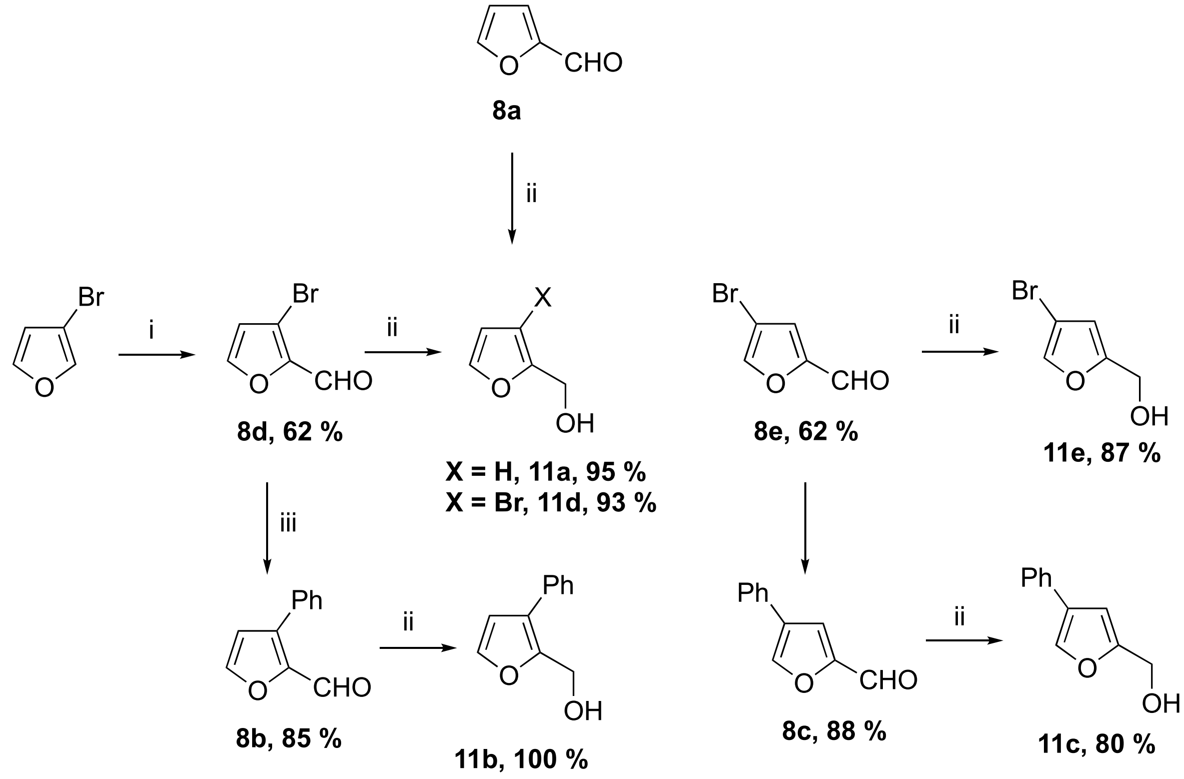

Scheme S1. Preparation of furfuryl alcohols starting from corresponding furfuryl aldehydes. Reagents and conditions: (i) DIPA, *n*-BuLi (2.5 M in Hexanes), DMF, THF, -78 °C to rt, 24h; 62%; (ii) $NaBH_4$, MeOH, 0 °C, 30 min; (iii) $Pd(PPh_3)_4$, Phenylboronic acid, K_2CO_3, 1,4-dioxane: H_2O (1:1), 110 °C, 48h.

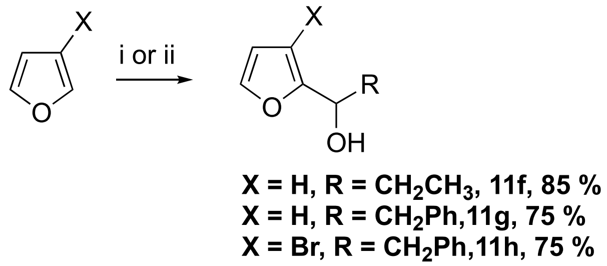

Scheme S2. Preparation of furfuryl alcohols starting from corresponding furan derivatives. Reagents and conditions: (i) *n*-BuLi (2.5 M in Hexanes), aldehyde derivative, THF, -78 °C to rt, 24h; (ii) DIPA, *n*-BuLi (2.5 M in Hexanes), THF, -78 °C, 5 min. and phenylacetaldehyde, -78 °C to rt, 16h.

3. CHARACTERIZATION OF COMPOUNDS

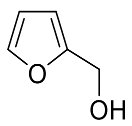

furan-2-ylmethanol (11a) was synthesized according to *general procedure 2.1.3.,* starting from furfural (10 g, 110 mmol) to yield **11a** (10.3 g, 105 mmol, 95%) as a colorless liquid; Rf:0.26 (20% EtOAc/*n*-Hexane).

^1H NMR ($CDCl_3$, 500 MHz): δ 7.39 (d, *J* = 1.0 Hz, 1H), 6.35 – 6.31 (m, 1H), 6.27 (d, *J* = 3.1 Hz, 1H), 4.54 (s, 2H), 3.23 (s, 1H) ppm.
$^{13}C\{^1H\}$ NMR ($CDCl_3$, 125 MHz): δ 154.1, 142.5, 110.3, 107.7, 57.1 ppm.

The data are good agreement with the literature.[1]

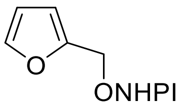

2-(furan-2-ylmethoxy)isoindoline-1,3-dione (9a) was synthesized according to *general procedure 2.4.,* starting from **11a** (5 g, 52.6 mmol) to yield **9a** (10.6 g, 43.6 mmol, 83%) as a white solid; m.p.: 125-127 °C; Rf:0.32 (20% EtOAc/*n*-Hexane).

^{1}H NMR (CDCl$_3$, 400 MHz): δ 7.81 (dd, *J* = 5.3, 3.2 Hz, 2H), 7.73 (dd, *J* = 5.6, 3.0 Hz, 2H), 7.47 (d, *J* = 1.8 Hz, 1H), 6.49 (d, *J* = 3.3 Hz, 1H), 6.35 – 6.34 (m, 1H), 5.16 (s, 2H) ppm.
^{13}C{^{1}H} NMR (CDCl$_3$, 101 MHz): δ 163.4, 148.0, 144.6, 134.5, 128.8, 123.6, 113.4, 110.9, 70.4 ppm.
IR (ATR): ν = 3240, 3133, 3092, 3042, 2991, 1723 cm^{-1}.
HRMS (ESI): (m/z) [M + H]$^+$ Calcd for $C_{13}H_9NO_4$ 244.0609; Found 244.0603.

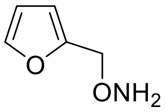

***O*-(furan-2-ylmethyl)hydroxylamine (12a)** was synthesized according to *general procedure 2.5.*, starting from **9a** (7 g, 28.8 mmol) to yield **12a** as a colorless liquid, which was not fully characterized and was used directly in the next step.

IR (ATR): ν = 3318, 3241, 3037, 2989, 2918, 1693 cm^{-1}.

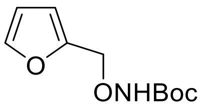

***tert*-butyl (furan-2-ylmethoxy)carbamate (5a)** was synthesized according to *general procedure 2.6.,* starting from **12a** (6 g, 52.9 mmol) to yield **5a** (7.9 g, 37.0 mmol, 72% overall) as a light-yellow liquid; Rf:0.54 (20% EtOAc/*n*-Hexane).

^{1}H NMR (CDCl$_3$, 500 MHz): δ 7.36 – 7.35 (m, 1H), 6.35 (d, *J* = 3.1 Hz, 1H), 6.28 (dd, *J* = 3.1, 1.9 Hz, 1H), 4.72 (s, 2H), 1.39 (s, 9H) ppm.
^{13}C{^{1}H} NMR (CDCl$_3$, 125 MHz): δ 156.7, 149.8, 143.5, 111.3, 110.5, 81.9, 69.7, 28.2 ppm.
IR (ATR): ν = 3305, 1685 cm^{-1}.
HRMS (ESI): (m/z) [M + Na]$^+$ Calcd for $C_{10}H_{15}NO_4Na$ 236.0899; Found 236.0894.

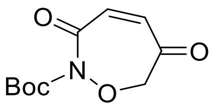

***tert*-butyl 3,6-dioxo-6,7-dihydro-1,2-oxazepine-2(3H)-carboxylate (10a)**
7a was synthesized according to *general procedure 2.7.,* starting from **5a** (1.08 g, 5.06 mmol) to yield **7a**. It was used in next step without further purification. **10a** was synthesized according to *general procedure 2.9.,* starting from **7a** (950 mg, 4.11 mmol) to yield **10a** (804 mg, 3.54 mmol, 70% overall) as a colorless liquid; Rf:0.83 (40% EtOAc/*n*-Hexane).

^{1}H NMR (CDCl$_3$, 500 MHz): δ 6.57 (d, *J* = 12.3 Hz, 1H), 6.38 (d, *J* = 12.4 Hz, 1H), 4.62 (s, 2H), 1.52 (s, 9H) ppm.
^{13}C{^{1}H} NMR (CDCl$_3$, 125 MHz): δ 198.3, 163.7, 148.1, 134.7, 133.2, 86.1, 82.0, 28.0 ppm.
IR (ATR): ν = 1772, 1753, 1699 cm^{-1}.
HRMS (ESI): (m/z) [M + Na]$^+$ Calcd for $C_{10}H_{13}NO_5Na$ 250.0691; Found 250.0693.

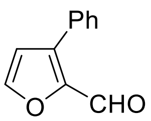

3-phenylfuran-2-carbaldehyde (8b) was synthesized according to *general procedure 2.2.,* starting from **8d** (1.08 g, 6.17 mmol) to yield **8b** (903 mg, 5.24 mmol, 85%) as a yellow liquid; Rf:0.54 (20% EtOAc/*n*-Hexane).

^{1}H NMR ($CDCl_3$, 500 MHz): δ 9.62 (s, 1H), 7.59 (s, 1H), 7.48 – 7.42 (m, 2H), 7.40 – 7.32 (m, 3H), 6.62 (d, *J* = 1.9 Hz, 1H) ppm.
^{13}C{^{1}H} NMR ($CDCl_3$, 125 MHz): δ 176.8, 146.5 (2x), 137.8, 129.4, 128.2, 128.0, 127.9, 112.6 ppm.
IR (ATR): ν = 3129, 3055, 2843, 1661, 1604, 1593, 1580, 1562, 1258, 1153, 1065 cm^{-1}.
HRMS (ESI): (m/z) $[M + H]^+$ Calcd for $C_{11}H_9O_2$ 173.0602; Found 173.0595.

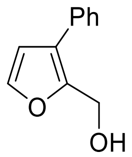

(3-phenylfuran-2-yl)methanol (11b) was synthesized according to *general procedure 2.1.3.,* starting from **8b** (2.94 g, 17 mmol) to yield **11b** (2.96 g, 17 mmol, 100%) as a yellow liquid; Rf:0.27 (20% EtOAc/*n*-Hexane).

^{1}H NMR ($CDCl_3$, 500 MHz): δ 7.43 – 7.30 (m, 5H), 7.25 (t, *J* = 7.3 Hz, 1H), 7.19 (s, 1H), 6.51 (s, 1H), 4.66 (s, 2H) ppm.
^{13}C{^{1}H} NMR ($CDCl_3$,125 MHz): δ 149.3, 142.1, 133.0, 128.8, 128.0, 127.2, 124.5, 111.5, 56.2 ppm.
IR (ATR): ν = 3378, 3052, 2955, 2930, 2848, 1010 cm^{-1}.
HRMS (ESI): (m/z) $[M + H]^+$ Calcd for $C_{11}H_{11}O_2$ 175.0759; Found 175.0744.

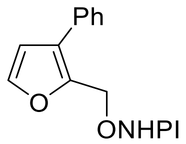

2-((3-phenylfuran-2-yl)methoxy)isoindoline-1,3-dione (9b) was synthesized according to *general procedure 2.4.,* starting from **11b** (1.42 g, 8.15 mmol) to yield **9b** (2.21 g, 6.93 mmol, 85%) as a yellow solid; m.p.: 149-151 °C; Rf:0.23 (20% EtOAc/*n*-Hexane).

^{1}H NMR ($CDCl_3$, 500 MHz): δ 7.65 (tdd, *J* = 9.3, 5.3, 2.4 Hz, 4H), 7.43 (d, *J* = 2.0 Hz, 1H), 7.39 (d, *J* = 7.5 Hz, 2H), 7.25 – 7.22 (m, 2H), 7.18 – 7.15 (m, 1H), 6.50 (d, *J* = 2.2 Hz, 1H), 5.20 (s, 2H) ppm.
^{13}C{^{1}H} NMR ($CDCl_3$, 125 MHz): δ 163.2, 143.9, 143.6, 134.3, 132.1, 129.5, 128.9, 128.7, 128.0, 127.5, 123.4, 111.8, 69.3 ppm.
IR (ATR): ν = 3149, 3059, 2956, 2930, 1789, 1730, 1509, 1256, 1185, 1082 cm^{-1}.
HRMS (ESI): (m/z) $[M + Na]^+$ Calcd for $C_{19}H_{13}NO_4Na$ 342.0742; Found 342.0723.

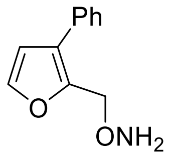

***O*-((3-phenylfuran-2-yl)methyl)hydroxylamine (12b)** was synthesized according to *general procedure 2.5.,* starting from **9b** (1.8 g, 5.64 mmol) to yield **12b** as a light-yellow liquid, which was not fully characterized and was used directly in the next step.

^{1}H NMR ($CDCl_3$, 500 MHz): δ 7.43 (d, *J* = 7.6 Hz, 2H), 7.37 (s, 1H), 7.33 – 7.29 (m, 2H), 7.25 – 7.20 (m, 1H), 6.50 (s, 1H), 5.43 (s, 2H), 4.65 (d, *J* = 1.9 Hz, 2H) ppm.
^{13}C{^{1}H} NMR ($CDCl_3$, 125 MHz): δ 147.0, 142.5, 133.0, 128.7, 128.6, 128.2, 128.0, 127.2, 111.4, 109.6, 68.5 ppm.
IR (ATR): ν = 3319, 3244, 3115, 3055, 3023, 2921, 2870, 1584, 1145 cm^{-1}.

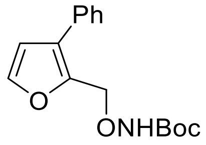

***tert*-butyl ((3-phenylfuran-2-yl)methoxy)carbamate (5b)** was synthesized according to *general procedure 2.6.,* starting from **12b** (1.22 g, 6.45 mmol) to yield **5b** (1.7 g, 5.87 mmol, 91%) as a yellow liquid; Rf:0.4 (20% EtOAc/*n*-Hexane).

^{1}H NMR ($CDCl_3$, 500 MHz): δ 7.43 (d, *J* = 7.6 Hz, 2H), 7.39 – 7.38 (m, 1H), 7.32 (t, *J* = 7.1 Hz, 3H), 7.26 – 7.20 (m, 1H), 6.50 (d, *J* = 1.8 Hz, 1H), 4.83 (s, 2H), 1.38 (s, 9H) ppm.
^{13}C{^{1}H} NMR ($CDCl_3$, 125 MHz): δ 156.7, 145.5, 143.0, 132.8, 128.8, 128.1, 127.4 (2x), 111.5, 81.9, 68.8, 28.2 ppm.
IR (ATR): ν = 3290, 3063, 2978, 2937, 1719, 1697, 1159, 1098 cm^{-1}.
HRMS (ESI): (m/z) [M + Na]$^+$ Calcd for $C_{16}H_{19}NO_4Na$ 312.1212; Found 312.1206.

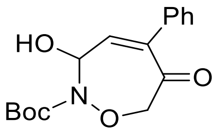

***tert*-butyl 3-hydroxy-6-oxo-5-phenyl-6,7-dihydro-1,2-oxazepine-2(*3H*)-carboxylate (7b)** was synthesized according to *general procedure 2.7.,* starting from **5b** (100 mg, 0.35 mmol) to yield **7b** (61 mg, 0.2 mmol, 57%) as a white solid; m.p.: 103-105 °C; Rf:0.14 (20% EtOAc/*n*-Hexane).

^{1}H NMR ($CDCl_3$, 500 MHz): δ 7.31 – 7.26 (m, 3H), 7.23 (dd, *J* = 6.8, 2.8 Hz, 2H), 6.40 (d, *J* = 3.3 Hz, 1H), 6.14 (dd, *J* = 8.8, 3.4 Hz, 1H), 4.67 (dd, *J* = 115.9, 16.6 Hz, 2H), 3.32 (d, *J* = 9.1 Hz, 1H), 1.48 (s, 9H) ppm.
^{13}C{^{1}H} NMR ($CDCl_3$, 125 MHz): δ 201.9, 154.8, 140.2, 137.7, 136.3, 128.6, 128.5 (2x), 83.9, 82.8, 82.0, 28.2 ppm.
IR (ATR): ν = 3397, 3059, 2979, 2920, 2852, 1690, 1153, 1099 cm^{-1}.
HRMS (ESI): (m/z) [M + Na]$^+$ Calcd for $C_{16}H_{19}NO_5Na$ 328.1161; Found 328.1141.

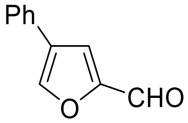

4-phenylfuran-2-carbaldehyde (8c) was synthesized according to *general procedure 2.2.,* starting from 3-bromo-2-furaldehyde (**8e**) (1.5 g, 8.6 mmol) to yield **8c** (1.3 g, 7.57 mmol, 88%) as a yellow liquid; Rf:0.53 (20% EtOAc/*n*-Hexane).

^{1}H NMR ($CDCl_3$, 500 MHz): δ 9.63 (s, 1H), 7.87 (s, 1H), 7.43 (d, *J* = 9.0 Hz, 3H), 7.35 (t, *J* = 7.5 Hz, 2H), 7.27 (t, *J* = 7.5 Hz, 1H) ppm.
^{13}C{^{1}H} NMR ($CDCl_3$, 125 MHz): δ 177.1, 152.6, 142.7, 129.4, 128.3, 128.1, 127.1 (2x), 125.0 ppm.
IR (ATR): ν = 3129, 3055, 3030, 2916, 2848, 1674, 1522, 1147, 1049 cm^{-1}.
HRMS (ESI): (m/z) [M + H]$^+$ Calcd for $C_{11}H_9O_2$ 173.0602; Found 173.0599.

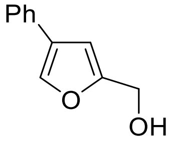

(4-phenylfuran-2-yl)methanol (11c) was synthesized according to *general procedure 2.1.3.,* starting from **8c** (1.3 g, 7.55 mmol) to yield **11c** (1.05 g, 6.04 mmol, 80%) as a white solid; m.p.: 134-136 °C; Rf:0.24 (20% EtOAc/*n*-Hexane).

^{1}H NMR ($CDCl_3$, 500 MHz): δ 7.61 (s, 1H), 7.39 (d, *J* = 7.3 Hz, 2H), 7.30 (d, *J* = 7.9 Hz, 2H), 7.19 (d, *J* = 8.1 Hz, 1H), 6.54 (s, 1H), 4.57 (s, 2H) ppm.

$^{13}C\{^1H\}$ NMR ($CDCl_3$, 125 MHz): δ 155.0, 138.4, 132.3, 128.9, 127.3, 127.1, 125.8, 107.1, 57.7 ppm.
IR (ATR): ν = 3281, 3059, 3033, 2919, 2852, 1451, 1131 cm^{-1}.
HRMS (ESI): (m/z) $[M + H]^+$ Calcd for $C_{11}H_{11}O_2$ 175.0759; Found 175.0745.

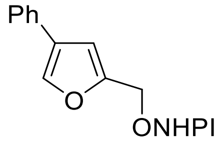

2-((4-phenylfuran-2-yl)methoxy)isoindoline-1,3-dione (9c) was synthesized according to *general procedure 2.4.,* starting from **11c** (957 mg, 5.5 mmol) to yield **9c** (1.58 g, 4.95 mmol, 90%) as a yellow solid; m.p.: 99-100 °C; Rf:0.23 (20% EtOAc/*n*-Hexane).

^1H NMR ($CDCl_3$, 500 MHz): δ 7.74 (tt, *J* = 6.1, 4.5, 3.2 Hz, 2H), 7.70 – 7.64 (m, 3H), 7.35 (d, *J* = 7.6 Hz, 2H), 7.27 (t, *J* = 7.6 Hz, 2H), 7.18 (d, *J* = 6.9 Hz, 1H), 6.74 (s, 1H), 5.13 (s, 2H) ppm.
$^{13}C\{^1H\}$ NMR ($CDCl_3$, 125 MHz): δ 163.4, 149.0, 140.2, 134.5, 131.8, 128.9, 128.8 (2x), 127.2, 125.8, 123.6, 112.2, 70.6 ppm.
IR (ATR): ν = 3096, 3079, 3026, 2933, 2855, 1766, 1725, 1613, 1375, 1186, 1081 cm^{-1}.
HRMS (ESI): (m/z) $[M + H]^+$ Calcd for $C_{19}H_{14}NO_4$ 320.0923; Found 320.0916.

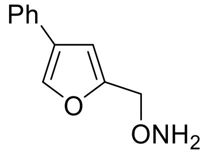

***O*-((4-phenylfuran-2-yl)methyl)hydroxylamine (12c)** was synthesized according to *general procedure 2.5.,* starting from **9c** (1 g, 3.13 mmol) to yield **12c** as a light-yellow liquid, which was not fully characterized and was used directly in the next step.

^1H NMR ($CDCl_3$, 500 MHz): δ 7.61 (s, 1H), 7.39 – 7.33 (m, 2H), 7.30 – 7.22 (m, 2H), 7.16 (t, *J* = 7.5 Hz, 1H), 6.60 (s, 1H), 5.63 – 4.84 (m, 2H), 4.55 (s, 2H) ppm.
$^{13}C\{^1H\}$ NMR ($CDCl_3$, 125 MHz): δ 152.5, 138.8, 132.3, 128.9 (2x), 127.1, 125.8, 109.2, 69.6 ppm.
IR (ATR): ν = 3316, 3237, 3030, 2921, 2863, 1582, 1353, 1133 cm^{-1}.

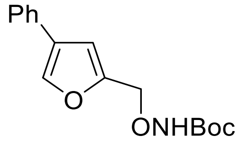

***tert*-butyl ((4-phenylfuran-2-yl)methoxy)carbamate (5c)** was synthesized according to *general procedure 2.6.,* starting from **12c** (281 mg, 1.48 mmol) to yield **5c** (304 mg, 1.05 mmol, 71%) as a colorless liquid; Rf:0.28 (20% EtOAc/*n*-Hexane).

^1H NMR ($CDCl_3$, 500 MHz): δ 7.65 (s, 1H), 7.42 – 7.37 (m, 2H), 7.30 (t, *J* = 7.6 Hz, 2H), 7.20 (t, *J* = 6.0 Hz, 1H), 7.11 (s, 1H), 6.67 (s, 1H), 4.76 (s, 2H), 1.41 (s, 9H) ppm.
$^{13}C\{^1H\}$ NMR ($CDCl_3$, 125 MHz): δ 156.9, 151.0, 139.2, 132.1, 128.9, 127.4, 127.2, 125.8, 110.4, 82.0, 69.8, 28.2 ppm.
IR (ATR): ν = 3294, 3055, 3030, 2979, 2933, 1710, 1367, 1157, 1104 cm^{-1}.
HRMS (ESI): (m/z) $[M + Na]^+$ Calcd for $C_{16}H_{19}NO_4Na$ 312.1212; Found 312.1206.

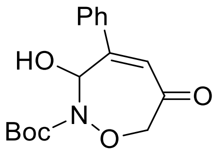

***tert*-butyl 3-hydroxy-6-oxo-4-phenyl-6,7-dihydro-1,2-oxazepine-2(*3H*)-carboxylate (7c)** was synthesized according to *general procedure 2.7.,* starting from **5c** (105 mg, 0.36 mmol) to yield **7c** (78 mg, 0.26 mmol, 71%) as a colorless liquid; Rf:0.14 (20% EtOAc/*n*-Hexane).

^{1}H NMR ($CDCl_3$, 500 MHz): δ 7.51 – 7.47 (m, 2H), 7.44 – 7.37 (m, 3H), 6.87 (d, *J* = 8.2 Hz, 1H), 6.35 (d, *J* = 1.4 Hz, 1H), 5.13 – 5.07 (m, 1H), 1.41 (s, 9H) ppm.
^{13}C{^{1}H} NMR ($CDCl_3$, 125 MHz): δ 170.5, 161.7, 154.2, 131.9, 129.3, 128.8, 127.6, 115.9, 83.3, 81.8, 29.7, 28.2 ppm.
IR (ATR): ν = 3318, 3007, 2981, 2925, 2855, 1754, 1688, 1628, 1522, 1252, 1155 cm^{-1}.
MALDI-TOF (matrix: DIT): (m/z) $[M – Boc]^+$ Calcd for $C_{11}H_{10}NO_3$ 204.0661; Found 204.0675.

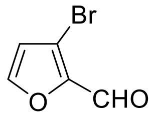

3-bromofuran-2-carbaldehyde (8d) was synthesized according to *general procedure 2.1.2,* starting from 3-bromofuran (5 g, 34 mmol) to yield **8d** (3.7 g, 21.1 mmol, 62%) as a yellow liquid; Rf:0.58 (20% EtOAc/*n*-Hexane).

^{1}H NMR ($CDCl_3$, 500 MHz): δ 9.66 (s, 1H), 7.57 (s, 1H), 6.60 (d, *J* = 1.7 Hz, 1H) ppm.
^{13}C{^{1}H} NMR ($CDCl_3$, 125 MHz): δ 176.3, 148.1, 148.0, 116.6, 112.6 ppm.
IR (ATR): ν = 3144, 3130, 2849, 2818, 1672, 1552, 1145, 1064, 1012 cm^{-1}.
LRMS (ESI): (m/z) $[M + H]^+$ Calcd for $C_5H_4BrO_2$ 174.94; Found 174.75.
The data are good agreement with the literature.[2]

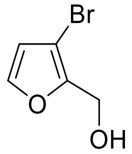

(3-bromofuran-2-yl)methanol (11d) was synthesized according to *general procedure 2.1.3,* starting from **8d** (1.51 g, 8.6 mmol) to yield **11d** (1.41 g, 8 mmol, 93%) as a yellow liquid; Rf:0.27 (20% EtOAc/*n*-Hexane).

^{1}H NMR ($CDCl_3$, 500 MHz): δ 7.30 (d, *J* = 2.0 Hz, 1H), 6.35 (d, *J* = 2.0 Hz, 1H), 4.57 (s, 2H) ppm.
^{13}C{^{1}H} NMR ($CDCl_3$, 125 MHz): δ 150.8, 142.9, 114.0, 98.7, 55.3 ppm.
IR (ATR): ν = 3350, 2852, 2923, 2854, 1504, 1186, 1075, 1021 cm^{-1}.
HRMS data for this compound have been previously reported.[3]
HRMS (ESI): (m/z) $[M - H_2O + H]^+$ Calcd for C_5H_4BrO 158.9445; Found 158.9428.

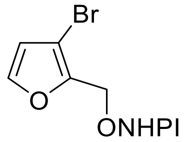

2-((3-bromofuran-2-yl)methoxy)isoindoline-1,3-dione (9d) was synthesized according to *general procedure 2.4.,* starting from **11d** (2.85 g, 16.1 mmol) to yield **9d** (4.67 g, 14.5 mmol, 90%) as a white solid; m.p.: 133-135 °C; Rf:0.34 (20% EtOAc/*n*-Hexane).

^{1}H NMR ($CDCl_3$, 500 MHz): δ 7.74 (dd, *J* = 5.5, 3.1 Hz, 2H), 7.67 (dd, *J* = 5.5, 3.1 Hz, 2H), 7.41 (d, *J* = 2.0 Hz, 1H), 6.33 (d, *J* = 2.0 Hz, 1H), 5.12 (s, 2H) ppm.
^{13}C{^{1}H} NMR ($CDCl_3$, 125 MHz): δ 162.2, 144.8, 143.7, 133.4, 127.9, 122.5, 113.3, 103.2, 66.9 ppm.
IR (ATR): ν = 3142, 3122, 3100, 3041, 1794, 1727, 1611, 1595, 1493, 1184, 1134, 1079, 1012 cm^{-1}.
HRMS (ESI): (m/z) $[M + H]^+$ Calcd for $C_{13}H_9BrNO_4$ 321.9715; Found 321.9702.

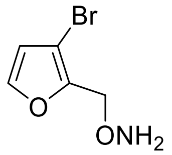

***O*-((3-bromofuran-2-yl)methyl)hydroxylamine (12d)** was synthesized according to *general procedure 2.5.,* starting from **9d** (3 g, 9.31 mmol) to yield **12d** as a colorless liquid, which was not fully characterized and was used directly in the next step.

^{1}H NMR ($CDCl_3$, 500 MHz): δ 7.32 (d, *J* = 2.0 Hz, 1H), 6.36 (d, *J* = 2.0 Hz, 1H), 5.45 (s, 2H), 4.58 (s, 2H) ppm.
^{13}C{^{1}H} NMR ($CDCl_3$, 125 MHz): δ 148.7, 143.2, 114.1, 100.8, 67.2 ppm.
IR (ATR): ν = 3318, 3244, 3148, 3124, 2920, 2867, 1584, 1501, 1139, 1351, 1078, 1010 cm^{-1}.

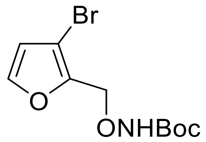

***tert*-butyl ((3-bromofuran-2-yl)methoxy)carbamate (5d)** was synthesized according to *general procedure 2.6.,* starting from **12d** (1.22 g, 6.45 mmol) to yield **5d** (1.8 g, 6.13 mmol, 95%) as a colorless liquid; Rf:0.4 (20% EtOAc/*n*-Hexane).

^{1}H NMR ($CDCl_3$, 500 MHz): δ 7.34 (d, *J* = 1.9 Hz, 1H), 7.07 (s, 1H), 6.38 (d, *J* = 1.9 Hz, 1H), 4.78 (s, 2H), 1.41 (s, 9H) ppm.
^{13}C{^{1}H} NMR ($CDCl_3$, 125 MHz): δ 156.6, 147.4, 143.7, 114.2, 102.3, 82.0, 67.3, 28.2 ppm.
IR (ATR): ν = 3289, 3152, 3130, 2979, 2930, 2870, 1719, 1249, 1165, 1103 cm^{-1}.
HRMS (ESI): (m/z) $[M + Na]^+$ Calcd for $C_{10}H_{14}BrNO_4Na$ 314.0004; Found 313.9999.

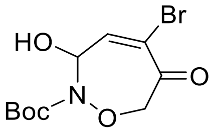

***tert*-butyl 5-bromo-3-hydroxy-6-oxo-6,7-dihydro-1,2-oxazepine-2(*3H*)-carboxylate (7d)** was synthesized according to *general procedure 2.7.,* starting from **5d** (200 mg, 0.68 mmol) to yield **7d** (200 mg, 0.65 mmol, 95%) as a white solid; Rf:0.34 (30% EtOAc/*n*-Hexane).

^{1}H NMR ($CDCl_3$, 500 MHz): δ 7.87 (s, 1H), 7.55 (s, 1H), 6.33 (s, 1H), 4.14 (d, *J* = 12.5 Hz, 1H), 4.03 (d, *J* = 12.5 Hz, 1H), 1.45 (s, 9H) ppm.
MALDI-TOF (matrix: IAA): (m/z) $[M+ Na]^+$ Calcd for $C_{10}H_{14}BrNO_5Na$ 329.9953; Found 329.9927.

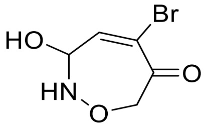

5-bromo-3-hydroxy-2,3-dihydro-1,2-oxazepin-6(*7H*)-one (7d) was synthesized according to *general procedure 2.8.,* starting from **5d** (60 mg, 0.21 mmol) to yield **7d** (30 mg, 0.14 mmol, 67%) as a white solid; m.p.: 148-150 ^{o}C; Rf:0.15 (30% EtOAc/*n*-Hexane).

^{1}H NMR ($CDCl_3$, 500 MHz): δ 7.28 (d, *J* = 3.4 Hz, 1H), 5.59 (d, *J* = 3.4 Hz, 1H), 4.68 (d, *J* = 16.7 Hz, 1H), 4.26 (d, *J* = 16.7 Hz, 1H) ppm.
^{13}C{^{1}H} NMR ($CDCl_3$, 125 MHz): δ 187.0, 145.9, 123.7, 90.1, 66.9 ppm.
IR (ATR): ν = 3437, 3401, 3059, 2922, 2844, 1710, 1614, 1424, 1316, 1130 cm^{-1}.
MALDI-TOF (matrix: SA): (m/z) $[M]^+$ Calcd for $C_5H_6BrNO_3$ 206.9531; Found 206.9531.

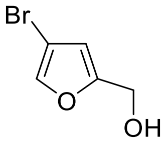

(4-bromofuran-2-yl)methanol (11e) was synthesized according to *general procedure 2.1.3.,* starting from 4-Bromo-2-furaldehyde (**8e**) (1.5 g, 8.57 mmol) to yield **11e** (1.32 g, 7.45 mmol, 87%) as a yellow liquid; Rf:0.3 (20% EtOAc/*n*-Hexane).

^{1}H NMR ($CDCl_3$, 500 MHz): δ 7.33 (s, 1H), 6.29 (s, 1H), 4.51 (s, 2H) ppm.
$^{13}C\{^1H\}$ NMR ($CDCl_3$, 125 MHz): δ 154.9, 140.8, 111.2, 100.1, 57.4 ppm.
IR (ATR): ν = 3311, 3150, 2925, 2869, 1592, 1223, 1124, 1009 cm^{-1}.
MALDI-TOF (matrix: DIT): (m/z) [M]$^+$ Calcd for $C_5H_5BrO_2$ 175.9473; Found 175.9471.

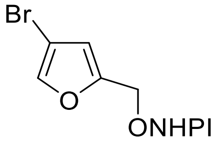

2-((4-bromofuran-2-yl)methoxy)isoindoline-1,3-dione (9e) was synthesized according to *general procedure 2.4.,* starting from **11e** (1.32 g, 7.46 mmol) to yield **9e** (2.21 g, 6.86 mmol, 92%) as a white solid; m.p.: 154-156 ^oC; Rf:0.27 (20% EtOAc/*n*-Hexane).

^{1}H NMR ($CDCl_3$, 500 MHz): δ 7.76 (dd, *J* = 5.4, 3.1 Hz, 2H), 7.69 (dd, *J* = 5.3, 3.0 Hz, 2H), 7.41 (s, 1H), 6.49 (s, 1H), 5.05 (s, 2H) ppm.
$^{13}C\{^1H\}$ NMR ($CDCl_3$, 125 MHz): δ 163.3, 149.1, 142.7, 134.6, 128.8, 123.7, 116.1, 100.3, 70.1 ppm.
IR (ATR): ν = 3142, 3110, 2919, 2851, 1774, 1720, 1607, 1252, 1133, 1081 cm^{-1}.
HRMS (ESI): (m/z) [M + H]$^+$ Calcd for $C_{13}H_9BrNO_4$ 321.9715; Found 321.9705.

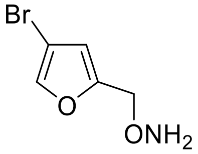

***O*-((4-bromofuran-2-yl)methyl)hydroxylamine (12e)** was synthesized according to *general procedure 2.5.,* starting from **9e** (1.47 g, 4.56 mmol) to yield **12e** as a light-yellow liquid, which was not fully characterized and was used directly in the next step.

^{1}H NMR ($CDCl_3$, 500 MHz): δ 7.42 (s, 1H), 6.43 (s, 1H), 5.27 (s, 2H), 4.58 (s, 2H) ppm.
$^{13}C\{^1H\}$ NMR ($CDCl_3$, 125 MHz): δ 152.6, 141.1, 113.1, 100.0, 69.2 ppm.
IR (ATR): ν = 3317, 3241, 3147, 3115, 2919, 2863, 1585, 1223, 1126 cm^{-1}.

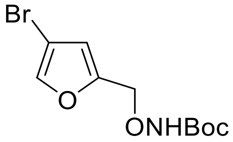

***tert*-butyl ((4-bromofuran-2-yl)methoxy)carbamate (5e)** was synthesized according to *general procedure 2.6.,* starting from **12e** (850 mg, 4.43 mmol) to yield **5e** (1.1 g, 3.67 mmol, 85%) as a yellow liquid; Rf:0.47 (20% EtOAc/*n*-Hexane).

^{1}H NMR ($CDCl_3$, 500 MHz): δ 7.37 (s, 1H), 7.04 (s, 1H), 6.41 (s, 1H), 4.69 (s, 2H), 1.40 (s, 9H) ppm.

$^{13}C\{^1H\}$ NMR ($CDCl_3$, 125 MHz): δ 156.6, 151.0, 141.6, 114.4, 100.1, 82.1, 69.5, 28.1 ppm.
IR (ATR): ν = 3281, 3144, 2978, 2928, 2874, 2855, 1715, 1247, 1162, 1104 cm^{-1}.
HRMS (ESI): (m/z) $[M + H]^+$ Calcd for $C_{10}H_{15}BrNO_4$ 292.0184; Found 292.0174.

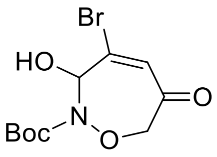

***tert*-butyl 4-bromo-3-hydroxy-6-oxo-6,7-dihydro-1,2-oxazepine-2(*3H*)-carboxylate (7e)** was synthesized according to *general procedure 2.7.,* starting from **5e** (400 mg, 1.37 mmol) to yield **7e** (317 mg, 1.03 mmol, 75%) as a light-yellow liquid; Rf:0.17 (20% EtOAc/*n*-Hexane).

^1H NMR ($CDCl_3$, 500 MHz): δ 6.53 (s, 1H), 5.97 (s, 1H), 4.69 – 4.40 (m, 2H), 3.98 (s, 1H), 1.46 (s, 9H) ppm.
$^{13}C\{^1H\}$ NMR ($CDCl_3$, 125 MHz): δ 196.9, 154.3, 142.4, 132.8, 85.6, 84.6, 80.9, 28.1 ppm.
IR (ATR): ν = 3376, 2985, 2941, 1720, 1683, 1600, 1150, 1072 cm^{-1}.
HRMS (ESI): (m/z) $[M + Na]^+$ Calcd for $C_{10}H_{14}BrNO_5Na$ 329.9953; Found 329.9944.

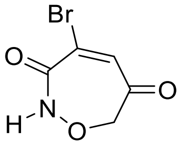

4-bromo-1,2-oxazepine-3,6(*2H*,*7H*)-dione (10e) was synthesized according to *general procedure 2.9.,* starting from **7e** (364 mg, 1.18 mmol) to yield **10e** (146 mg, 0.71 mmol, 60%) as a cream solid; m.p.: 138-140 °C; Rf:0.1 (20% EtOAc/*n*-Hexane).

^1H NMR ($CDCl_3$, 500 MHz): δ 8.93 (s, 1H), 7.18 (s, 1H), 4.61 (d, *J* = 2.9 Hz, 2H) ppm.
$^{13}C\{^1H\}$ NMR ($CDCl_3$, 125 MHz): δ 196.4, 165.8, 136.9, 133.6, 82.0 ppm.
IR (ATR): ν = 3223, 3074, 3023, 2975, 2926, 1677, 1655, 1648, 1585, 1244, 1107, 1011 cm^{-1}.
HRMS (ESI): (m/z) $[M + H]^+$ Calcd for $C_5H_5BrNO_3$ 205.9453; Found 205.9446.

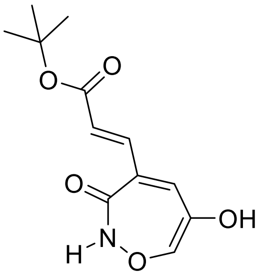

***tert*-butyl (E)-3-(6-hydroxy-3-oxo-2,3-dihydro-1,2-oxazepin-4-yl)acrylate (14e)** was synthesized according to *general procedure 2.3.,* starting from **10e** (100 mg, 0.48 mmol) to yield **14e** (117 mg, 0.46 mmol, 96%) as a yellow solid; m.p.: 107-109 °C; Rf:0.63 (30% EtOAc/*n*-Hexane).

^1H NMR ($CDCl_3$, 500 MHz): δ 9.85 (s, 1H), 7.88 (d, *J* = 15.9 Hz, 1H), 7.54 (d, *J* = 1.9 Hz, 1H), 6.69 (d, *J* = 2.1 Hz, 1H), 6.31 (d, *J* = 15.9 Hz, 1H), 1.46 (s, 9H) ppm.
$^{13}C\{^1H\}$ NMR ($CDCl_3$, 125 MHz): δ 178.2, 165.3, 149.2, 147.6, 130.8, 130.5, 126.2, 110.3, 81.3, 28.1 ppm.
IR (ATR): ν = 3411, 3205, 2970, 2924, 2852, 1721, 1655, 1464, 1383, 1365, 1147, 1055 cm^{-1}.
HRMS (APCI): (m/z) $[M + H]^+$ Calcd for $C_{12}H_{16}NO_5$ 254.1028; Found 254.1025.

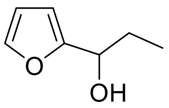

1-(furan-2-yl)propan-1-ol (11f) was synthesized according to *general procedure 2.1.1.,* starting from furan (5 g, 73.5 mmol) to yield **11f** (7.88 g, 62.5 mmol, 85%) as a light-yellow liquid; Rf:0.47 (20% EtOAc/*n*-Hexane).

^{1}H NMR ($CDCl_3$, 500 MHz): δ 7.30 (d, *J* = 2.2 Hz, 1H), 6.26 (dd, *J* = 3.3, 1.8 Hz, 1H), 6.16 (d, *J* = 3.1 Hz, 1H), 4.53 (t, *J* = 6.8 Hz, 1H), 1.81 (ddt, *J* = 16.5, 14.1, 6.9 Hz, 2H), 0.88 (t, *J* = 7.5 Hz, 3H) ppm.
^{13}C{^{1}H} NMR ($CDCl_3$, 125 MHz): δ 156.7, 141.9, 110.1, 105.9, 69.2, 28.6, 9.9 ppm.
IR (ATR): ν = 3363, 3115, 2966, 2935, 2878, 1667, 1504, 1149 cm^{-1}.
HRMS (ESI): (m/z) $[M + H]^+$ Calcd for $C_7H_{11}O_2$ 127.0759; Found 127.0751.

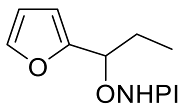

2-(1-(furan-2-yl)propoxy)isoindoline-1,3-dione (9f) was synthesized according to *general procedure 2.4.,* starting from **11f** (1.81 g, 14.3 mmol) to yield **9f** (3.04 g, 11.2 mmol, 78%) as a light-yellow solid; m.p.: 103-105 ^{o}C; Rf:0.34 (20% EtOAc/*n*-Hexane).

^{1}H NMR ($CDCl_3$, 500 MHz): δ 7.72 – 7.61 (m, 4H), 7.37 (s, 1H), 6.28 (d, *J* = 3.0 Hz, 1H), 6.22 (d, *J* = 2.9 Hz, 1H), 5.06 (t, *J* = 7.3 Hz, 1H), 2.16 (ddq, *J* = 53.0, 14.3, 7.2 Hz, 2H), 0.97 (t, *J* = 7.5 Hz, 3H) ppm.
^{13}C{^{1}H} NMR ($CDCl_3$, 125 MHz): δ 163.5, 150.7, 143.8, 134.3, 128.8, 123.4, 111.5, 110.4, 82.9, 24.4, 10.1 ppm.
IR (ATR): ν = 2973, 2933, 2880, 1789, 1728, 1609, 1372, 1186, 1081 cm^{-1}.
HRMS (ESI): (m/z) $[M + Na]^+$ Calcd for $C_{15}H_{13}NO_4Na$ 294.0742; Found 294.0736.

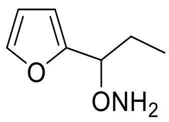

***O*-(1-(furan-2-yl)propyl)hydroxylamine (12f)** was synthesized according to *general procedure 2.5.,* starting from **9f** (1.4 g, 5.16 mmol) to yield **12f** as a light-yellow liquid, which was not fully characterized and was used directly in the next step.

^{1}H NMR ($CDCl_3$, 500 MHz): δ 7.32 (s, 1H), 6.35 – 6.17 (m, 2H), 5.15 (s, 2H), 4.36 (t, *J* = 7.1 Hz, 1H), 1.94 – 1.58 (m, 2H), 0.83 (td, *J* = 7.4, 1.8 Hz, 3H) ppm.
^{13}C{^{1}H} NMR ($CDCl_3$, 125 MHz): δ 154.3, 142.3, 110.1, 108.1, 81.3, 25.3, 10.0 ppm.
IR (ATR): ν = 3319, 3241, 2969, 2937, 2879, 1584, 1151 cm^{-1}.

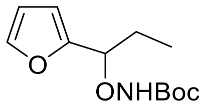

***tert*-butyl (1-(furan-2-yl)propoxy)carbamate (5f)** was synthesized according to *general procedure 2.6.,* starting from **12f** (560 mg, 3.97 mmol) to yield **5f** (642 mg, 2.66 mmol, 67%) as a colorless liquid; Rf:0.47 (20% EtOAc/*n*-Hexane).

^{1}H NMR ($CDCl_3$, 500 MHz): δ 7.35 (s, 1H), 6.89 (s, 1H), 6.28 (d, *J* = 2.0 Hz, 2H), 4.60 (t, *J* = 7.2 Hz, 1H), 1.89 (ddq, *J* = 63.9, 14.1, 7.2 Hz, 2H), 1.40 – 1.37 (m, 9H), 0.89 (t, *J* = 7.4 Hz, 3H) ppm.
^{13}C{^{1}H} NMR ($CDCl_3$, 125 MHz): δ 156.5, 152.8, 142.7, 110.2, 109.4, 81.8, 81.6, 28.2, 24.8, 10.1 ppm.
IR (ATR): ν = 3300, 3119, 2976, 2936, 2877, 1697, 1367, 1157 cm^{-1}.
HRMS (ESI): (m/z) $[M + Na]^+$ Calcd for $C_{12}H_{19}NO_4Na$ 264.1212; Found 264.1205.

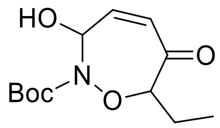

***tert*-butyl 7-ethyl-3-hydroxy-6-oxo-6,7-dihydro-1,2-oxazepine-2(*3H*)-carboxylate (7f)** was synthesized according to *general procedure 2.7.,* starting from **5f** (192 mg, 0.79 mmol) to yield **7f** (189 mg, 0.73 mmol, 93%) as a colorless oil; diastereomeric mixture. Rf:0.17 (20% EtOAc/*n*-Hexane).

^{1}H NMR (CDCl$_3$, 500 MHz): δ 6.40 (s, 1H), 6.22 – 6.19 (m, 1H), 5.90 (d, *J* = 12.1 Hz, 1H), 4.43 – 4.37 (m, 1H), 1.67 (qt, *J* = 16.9, 8.0 Hz, 2H), 1.48 (s, 9H), 1.00 (t, *J* = 7.5 Hz, 3H) ppm.
^{13}C{^{1}H} NMR (CDCl$_3$, 125 MHz): δ 204.4, 155.2, 138.7, 128.8, 93.6, 88.4, 83.7, 28.2, 24.9, 10.1 ppm.
IR (ATR): ν = 3432, 2978, 2933, 2881, 1716, 1678, 1370, 1160, 1046 cm^{-1}.
HRMS (ESI): (m/z) [M + Na]$^+$ Calcd for $C_{12}H_{19}NO_5Na$ 280.1161; Found 280.1160.

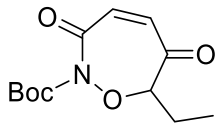

***tert*-butyl 7-ethyl-3,6-dioxo-6,7-dihydro-1,2-oxazepine-2(*3H*)-carboxylate (10f)** was synthesized according to *general procedure 2.9.,* starting from **7f** (180 mg, 0.7 mmol) to yield **10f** (155 mg, 0.61 mmol, 87%) as a cream solid; m.p.: 125-127 °C; Rf:0.37 (20% EtOAc/*n*-Hexane).

^{1}H NMR (CDCl$_3$, 500 MHz): δ 6.51 (d, *J* = 12.1 Hz, 1H), 6.35 (d, *J* = 12.2 Hz, 1H), 4.43 (dd, *J* = 8.6, 4.0 Hz, 1H), 1.93 – 1.67 (m, 2H), 1.51 (s, 9H), 1.04 (t, *J* = 7.4 Hz, 3H) ppm.
^{13}C{^{1}H} NMR (CDCl$_3$, 125 MHz): δ 201.5, 164.2, 148.3, 134.1, 133.8, 94.1, 85.7, 28.0, 25.2, 9.8 ppm.
IR (ATR): ν = 3048, 2980, 2936, 2881, 1772, 1752, 1692, 1318, 1139 cm^{-1}.
HRMS (ESI): (m/z) [M + Na]$^+$ Calcd for $C_{12}H_{17}NO_5Na$ 278.1004; Found 278.0996.

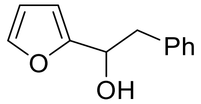

1-(furan-2-yl)-2-phenylethanol (11g) was synthesized according to *general procedure 2.1.1.,* starting from furan (5 g, 73.4 mmol) to yield **11g** (10.4 g, 55 mmol, 75%) as a colorless liquid; Rf:0.4 (20% EtOAc/*n*-Hexane).

^{1}H NMR (CDCl$_3$, 500 MHz): δ 7.33 (dd, *J* = 1.8, 0.8 Hz, 1H), 7.27 – 7.19 (m, 2H), 7.19 – 7.13 (m, 3H), 6.25 (dd, *J* = 3.2, 1.8 Hz, 1H), 6.14 (d, *J* = 3.2 Hz, 1H), 4.86 – 4.83 (m, 1H), 3.12 (dd, *J* = 13.7, 5.4 Hz, 1H), 3.04 (dd, *J* = 13.7, 8.1 Hz, 1H), 1.94 (s, 1H) ppm.
^{13}C{^{1}H} NMR (CDCl$_3$, 125 MHz): δ 156.0, 142.2, 137.6, 129.6, 128.7, 126.9, 110.4, 106.6, 69.0, 42.4 ppm.
IR (ATR): ν = 3368 cm^{-1}.
HRMS (ESI): (m/z) [M]$^+$ Calcd for $C_{12}H_{12}O_2$ 188.0837; Found 188.0837.

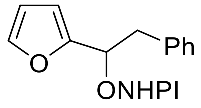

2-(1-(furan-2-yl)-2-phenylethoxy)isoindoline-1,3-dione (9g) was synthesized according to *general procedure 2.4.,* starting from **11g** (3.4 g, 18.5 mmol) to yield **9g** (4.8 g, 14.4 mmol, 78%) as a yellow solid; m.p.: 102-104 °C; Rf:0.5 (20% EtOAc/*n*-Hexane).

^{1}H NMR (CDCl$_3$, 500 MHz): δ 7.71 –7.65 (m, 2H), 7.64 – 7.61 (m, 2H), 7.40 (s, 1H), 7.19 – 7.09 (m, 5H), 6.20 (s, 1H), 6.16 (s, 1H), 5.39 (t, *J* = 7.4 Hz, 1H), 3.51 (dd, *J* = 13.9, 6.4 Hz, 1H), 3.44 (dd, *J* = 13.9, 8.4 Hz, 1H) ppm.

$^{13}C\{^1H\}$ NMR ($CDCl_3$, 125 MHz): δ 163.3, 149.7, 143.9, 136.2, 134.4, 129.2, 128.7, 128.4, 126.7, 123.4, 112.5, 110.5, 81.8, 37.4 ppm.
IR (ATR): ν = 3137, 3065, 3033, 1726 cm^{-1}.
HRMS (ESI): (m/z) [M]$^+$ Calcd for $C_{20}H_{15}NO_4$ 333.1001; Found 333.1007.

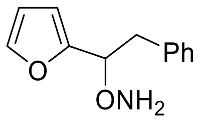

***O*-(1-(furan-2-yl)-2-phenylethyl)hydroxylamine (12g)** was synthesized according to *general procedure 2.5.,* starting from **9g** (4.2 g, 12.4 mmol) to yield **12g** as a light-yellow liquid, which was not fully characterized and was used directly in the next step.

IR (ATR): ν = 3322, 3248, 3148, 3118, 3063, 3026, 2952, 2918 cm^{-1}.

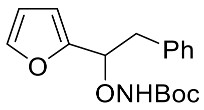

***tert*-butyl (1-(furan-2-yl)-2-phenylethoxy)carbamate (5g)** was synthesized according to *general procedure 2.6.,* starting from **12g** (1.52 g, 12.3 mmol) to yield **5g** (3 g, 9.9 mmol, 85% overall) as a light-yellow liquid; Rf:0.8 (20% EtOAc/*n*-Hexane).

^1H NMR ($CDCl_3$, 400 MHz): δ 7.43 (dd, *J* = 1.7, 0.7 Hz, 1H), 7.25 – 7.20 (m, 2H), 7.19 – 7.11 (m, 3H), 6.31 (dd, *J* = 3.2, 1.8 Hz, 1H), 6.27 (m, 1H), 4.97 (dd, *J* = 8.4, 6.2 Hz, 1H), 3.35 (dd, *J* = 13.7, 6.2 Hz, 1H), 3.22 (dd, *J* = 13.7, 8.4 Hz, 1H), 1.43 (s, 9H) ppm.
$^{13}C\{^1H\}$ NMR ($CDCl_3$, 101 MHz): δ 156.7, 151.9, 143.0, 137.1, 129.5, 128.5, 126.7, 110.7, 110.5, 81.9, 81.3, 38.2, 28.4 ppm.
IR (ATR): ν = 3306, 1742 cm^{-1}.
HRMS (ESI): (m/z) [M + Na]$^+$ Calcd for $C_{17}H_{21}NO_4Na$ 326.1368; Found 326.1363.

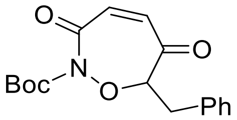

tert-butyl 7-benzyl-3,6-dioxo-6,7-dihydro-1,2-oxazepine-2(3H)-carboxylate (10g)
7g was synthesized according to *general procedure 2.7.,* starting from **5g** (400 mg, 13.9 mmol) to yield **7g**. It was used in next step without further purification. **10g** was synthesized according to *general procedure 2.9.,* starting from **7g** (371 mg, 1.16 mmol) to yield **10g** (360 mg, 1.13 mmol, 80% overall) as a colorless liquid; Rf:0.83 (40% EtOAc/*n*-Hexane).

^1H NMR ($CDCl_3$, 400 MHz): δ 7.30 – 7.20 (m, 5H), 6.45 (d, *J* = 12.2 Hz, 1H), 6.22 (d, *J* = 12.2 Hz, 1H), 4.83 (dd, *J* = 6.6, 4.2 Hz, 1H), 3.23 (dd, *J* = 14.6, 4.1 Hz, 1H), 3.14 (dd, *J* = 14.6, 6.7 Hz, 1H), 1.46 (s, 9H) ppm.
$^{13}C\{^1H\}$ NMR ($CDCl_3$, 101 MHz): δ 201.1, 164.3, 148.3, 134.9, 134.0, 133.8, 130.2, 128.7, 127.4, 93.3, 85.8, 37.9, 28.1 ppm.
IR (ATR): ν = 1753, 1773, 1696 cm^{-1}.
HRMS (ESI): (m/z) [M + Na]$^+$ Calcd for $C_{17}H_{19}NO_5Na$ 340.1161; Found 340.1155.

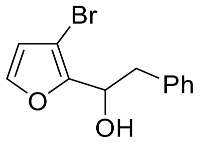

1-(3-bromofuran-2-yl)-2-phenylethanol (11h) was synthesized according to *general procedure 2.1.2.,* starting from 3-bromofuran (5 g, 34 mmol) to yield **11h** (7.1 g, 26.5 mmol, 75%) as a light-yellow liquid; Rf:0.36 (10% EtOAc/*n*-Hexane).

^{1}H NMR (CDCl$_3$, 500 MHz): δ 7.30 (s, 1H), 7.23 – 7.15 (m, 3H), 7.10 (d, *J* = 7.5 Hz, 2H), 6.30 (s, 1H), 4.96 (t, *J* = 7.0 Hz, 1H), 3.14 (dd, *J* = 13.6, 8.0 Hz, 1H), 3.05 (dd, *J* = 13.6, 6.2 Hz, 1H), 1.97 (s, 1H) ppm.
^{13}C{^{1}H} NMR (CDCl$_3$, 125 MHz): δ 151.4, 142.3, 136.9, 129.3, 128.5, 126.8, 113.9, 97.7, 66.9, 41.7 ppm.
IR (ATR): ν = 3368, 3028, 2927 cm^{-1}.
LRMS (ESI): (m/z) [M - H]$^+$ Calcd for $C_{12}H_{10}BrO_2$ 264.98; Found 264.97.

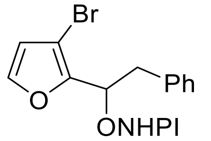

2-(1-(3-bromofuran-2-yl)-2-phenylethoxy)isoindoline-1,3-dione (9h) was synthesized according to *general procedure 2.4.,* starting from **11h** (4.5 g, 16.8 mmol) to yield **9h** (4.16 g, 10.1 mmol, 60%) as a light-yellow oil; Rf:0.4 (20% EtOAc/*n*-Hexane).

^{1}H NMR (CDCl$_3$, 500 MHz): δ 7.67 (ddd, *J* = 31.0, 5.1, 3.2 Hz, 4H), 7.45 (s, 1H), 7.23 – 7.12 (m, 5H), 6.22 – 6.21 (m, 1H), 5.54 (t, *J* = 7.5 Hz, 1H), 3.58 (dd, *J* = 14.1, 6.7 Hz, 1H), 3.44 (dd, *J* = 14.1, 8.4 Hz, 1H) ppm.
^{13}C{^{1}H} NMR (CDCl$_3$, 125 MHz): δ 163.2, 146.7, 144.2, 135.6, 134.4, 129.1, 128.8, 128.5, 126.9, 123.4, 114.0, 103.8, 79.1, 36.4 ppm.
IR (ATR): ν = 3142, 3125, 3033, 2925, 2852, 1728 cm^{-1}.
LRMS (ESI): (m/z) [M – $C_8H_4NO_2$]$^+$ Calcd for $C_{20}H_{14}BrNO_4$ 264.99; Found 264.94.

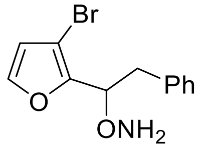

***O*-(1-(3-bromofuran-2-yl)-2-phenylethyl)hydroxylamine (12h)** was synthesized according to *general procedure 2.5.,* starting from **9h** (1.3 g, 3.15 mmol) to yield **12h** as a light-yellow liquid, which was not fully characterized and was used directly in the next step.

IR (ATR): ν = 3322, 3248, 3150, 3120, 3022, 2950 cm^{-1}.

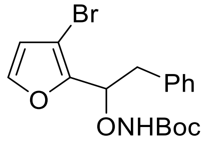

***tert*-butyl (1-(3-bromofuran-2-yl)-2-phenylethoxy)carbamate (5h)** was synthesized according to *general procedure 2.6.,* starting from **12h** (814 mg, 2.9 mmol) to yield **5h** (770 mg, 2 mmol, 63% overall) as a light-yellow liquid; Rf:0.56 (20% EtOAc/*n*-Hexane).

^{1}H NMR (CDCl$_3$, 500 MHz): δ 7.41 – 7.40 (m, 1H), 7.25 – 7.12 (m, 5H), 7.00 (s, 1H), 6.36 (d, *J* = 1.6 Hz, 1H), 5.19 – 5.16 (m, 1H), 3.37 (dd, *J* = 13.7, 6.5 Hz, 1H), 3.20 (dd, *J* = 13.7, 8.6 Hz, 1H), 1.44 (s, 9H) ppm.
^{13}C{^{1}H} NMR (CDCl$_3$, 125 MHz): δ 156.4, 148.5, 143.0, 136.4, 129.1, 128.4, 126.6, 114.00, 101.9, 81.8, 78.7, 37.3, 28.2 ppm.
IR (ATR): ν = 3300, 2981, 1711 cm^{-1}.
HRMS (ESI): (m/z) [M + Na]$^+$ Calcd for $C_{17}H_{20}BrNO_4Na$ 404.0473; Found 404.0471.

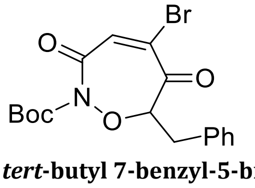

***tert*-butyl 7-benzyl-5-bromo-3,6-dioxo-6,7-dihydro-1,2-oxazepine-2(*3H*)-carboxylate (10h)**

7h was synthesized according to *general procedure 2.7.,* starting from **5h** (50 mg, 0.13 mmol) to yield **7h**. It was used in next step without further purification. **10h** was synthesized according to *general procedure 2.9.,* starting from **7h** (47 mg, 0.12 mmol) to yield **10h** (46 mg, 0.12 mmol, 89% overall) as a colorless liquid; Rf:0.80 (40% EtOAc/*n*-Hexane).

^{1}H NMR ($CDCl_3$, 500 MHz): δ 7.32 (d, *J* = 7.1 Hz, 3H), 7.18 (d, *J* = 6.4 Hz, 2H), 6.55 (s, 1H), 5.28 (s, 1H), 3.82 (d, *J* = 16.7 Hz, 1H), 3.70 (d, *J* = 16.1 Hz, 1H), 1.42 (s, 9H) ppm.
$^{13}C\{^{1}H\}$ NMR ($CDCl_3$, 125 MHz): δ 198.8, 165.1, 147.2, 143.1, 131.5, 129.6, 129.6, 128.8, 127.7, 92.7, 85.4, 53.4, 40.9, 30.9, 29.7, 27.8 ppm.
IR (ATR): ν = 3407, 2977, 1788, 1740 cm^{-1}.
HRMS (ESI): (m/z) $[M + Na]^+$ Calcd for $C_{17}H_{18}BrNO_5Na$ 418.0266; Found 418.0257.

4. X-RAY CRYSTALLOGRAPHIC DATA

X-ray crystallographic data and structure refinement

Data for the single crystal compound were acquired with Bruker APEX II QUAZAR three-circle diffractometer. Crystal structure validations and geometrical calculations were applied using the Platon software.[4] Mercury software was used for visualization of the .cif files.[5] The structures (Figure S1 and Figure S2) have been solved by the Bruker SHELXTL Software Package and refined using OLEX2.refine.[6,7] The software used for molecular graphics: OLEX2 1.3; to prepare material for publication: OLEX2 1.3. Data integration and reduction were realized with SAINT.[8] The crystallographic refinement data is summarized in Table S1 and Table S2.

Sample Preparation for Crystal Growth. The compound **7b** was dissolved in minimum amount of Dichloromethane and kept at room temperature for slow evaporation (4 days). Needle-shaped crystal was formed which was subjected to X-ray diffraction analysis.

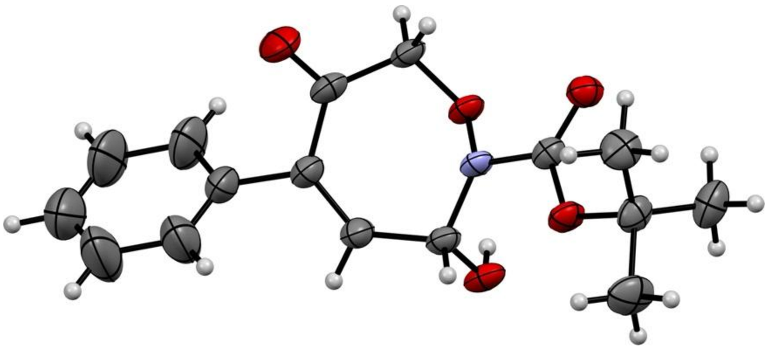

Figure S1: XRAY structure of *tert*-butyl 3-hydroxy-6-oxo-5-phenyl-6,7-dihydro-1,2-oxazepine-2(*3H*)-carboxylate (**7b**) with 30% ellipsoid (CCDC 2465168).

**Table S1:** Crystal data and structure refinement for **7b**.	
Identification code	23gtu294_FAL_1020_0m_a
Empirical formula	$C_{16}H_{19}NO_5$
Formula weight	305.333
Temperature/K	296.15
Crystal system	triclinic
Space group	P-1
a/Å	6.774(3)
b/Å	9.965(4)
c/Å	12.846(5)
α/°	86.745(6)
β/°	89.369(6)
γ/°	74.872(6)
Volume/Å^3	835.8(6)

Z	2
$\rho_{calc}g/cm^3$	1.213
μ/mm^{-1}	0.090
F(000)	324.2
Crystal size/mm^3	0.138 × 0.109 × 0.086
Radiation	Mo Kα (λ = 0.71073)
2Θ range for data collection/°	3.18 to 50.02
Index ranges	-8 ≤ h ≤ 8, -11 ≤ k ≤ 11, -15 ≤ l ≤ 15
Reflections collected	10346
Independent reflections	2939 [R_{int} = 0.0773, R_{sigma} = 0.0936]
Data/restraints/parameters	2939/3/204
Goodness-of-fit on F^2	1.000
Final R indexes [I>=2σ (I)]	R_1 = 0.0748, wR_2 = 0.2038
Final R indexes [all data]	R_1 = 0.1500, wR_2 = 0.2518
Largest diff. peak/hole / e $Å^{-3}$	0.52/-0.44

Sample Preparation for Crystal Growth. The compound **10e** was dissolved in minimum amount of DCM/*n*-Hexane (1:1) and kept at room temperature for slow evaporation (7 days). Needle-shaped crystal was formed which was subjected to X-ray diffraction analysis.

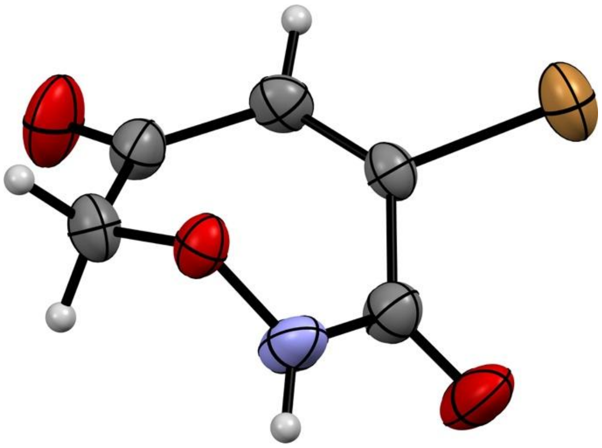

Figure S2: XRAY structure of 4-bromo-1,2-oxazepine-3,6(2H,7H)-dione (**10e**) with 30% ellipsoid (CCDC 2465167).

**Table S2:** Crystal data and structure refinement for **10e**.	
Identification code	23gtu293_FAL1078_10_0m_a
Empirical formula	$C_5H_4BrNO_3$
Formula weight	205.996
Temperature/K	273.15
Crystal system	monoclinic
Space group	$P2_1/c$
a/Å	11.001(7)
b/Å	7.824(3)
c/Å	7.773(6)
α/°	90
β/°	93.00(5)
γ/°	90
Volume/$Å^3$	668.1(7)
Z	4

ρ_{calc}g/cm^3	2.048
μ/mm^{-1}	6.092
F(000)	399.4
Crystal size/mm^3	0.163 × 0.115 × 0.114
Radiation	Mo Kα (λ = 0.71073)
2Θ range for data collection/°	3.7 to 54.98
Index ranges	-14 ≤ h ≤ 13, -10 ≤ k ≤ 10, -10 ≤ l ≤ 10
Reflections collected	6697
Independent reflections	1532 [R_{int} = 0.0421, R_{sigma} = 0.0405]
Data/restraints/parameters	1532/0/91
Goodness-of-fit on F^2	1.015
Final R indexes [I>=2σ (I)]	R_1 = 0.0344, wR_2 = 0.0669
Final R indexes [all data]	R_1 = 0.0631, wR_2 = 0.0753
Largest diff. peak/hole / e Å^{-3}	0.66/-0.67

5. ^{1}H AND ^{13}C NMR SPECTRA

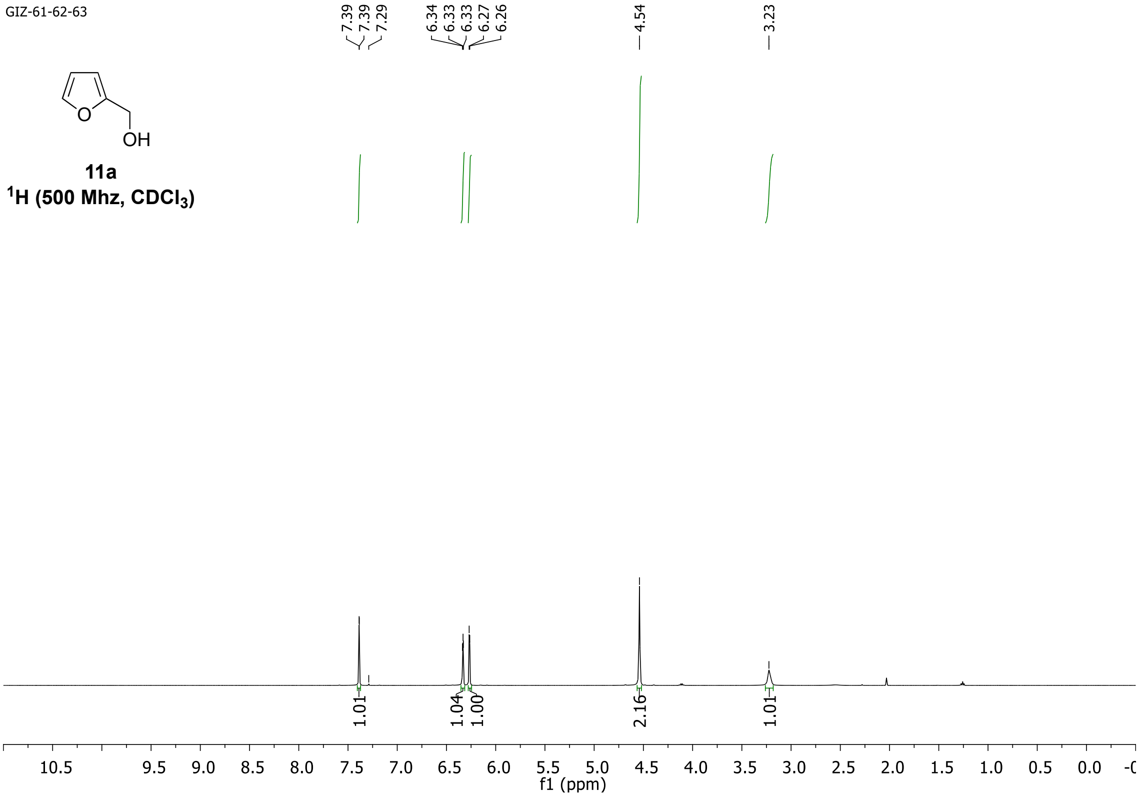

^{1}H NMR Spectrum of furan-2-ylmethanol (**11a**)

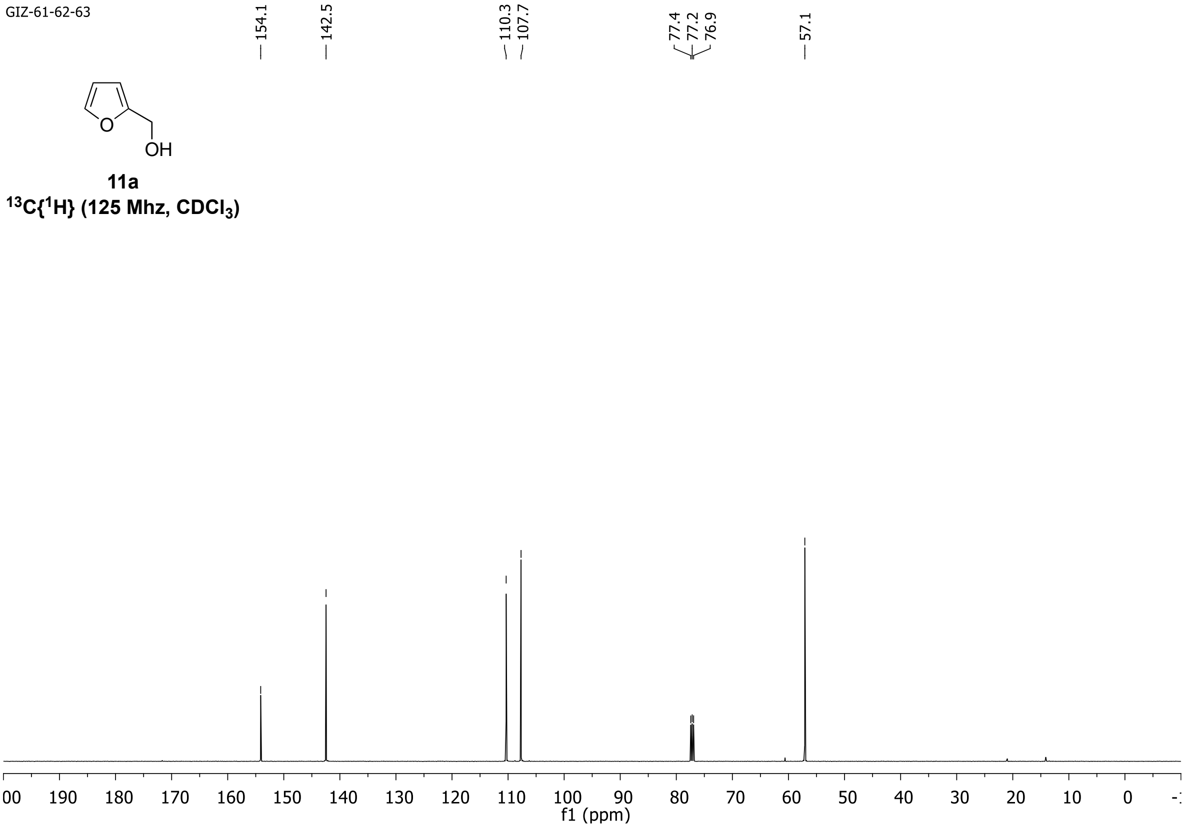

$^{13}C\{^{1}H\}$ NMR Spectrum of furan-2-ylmethanol (**11a**)

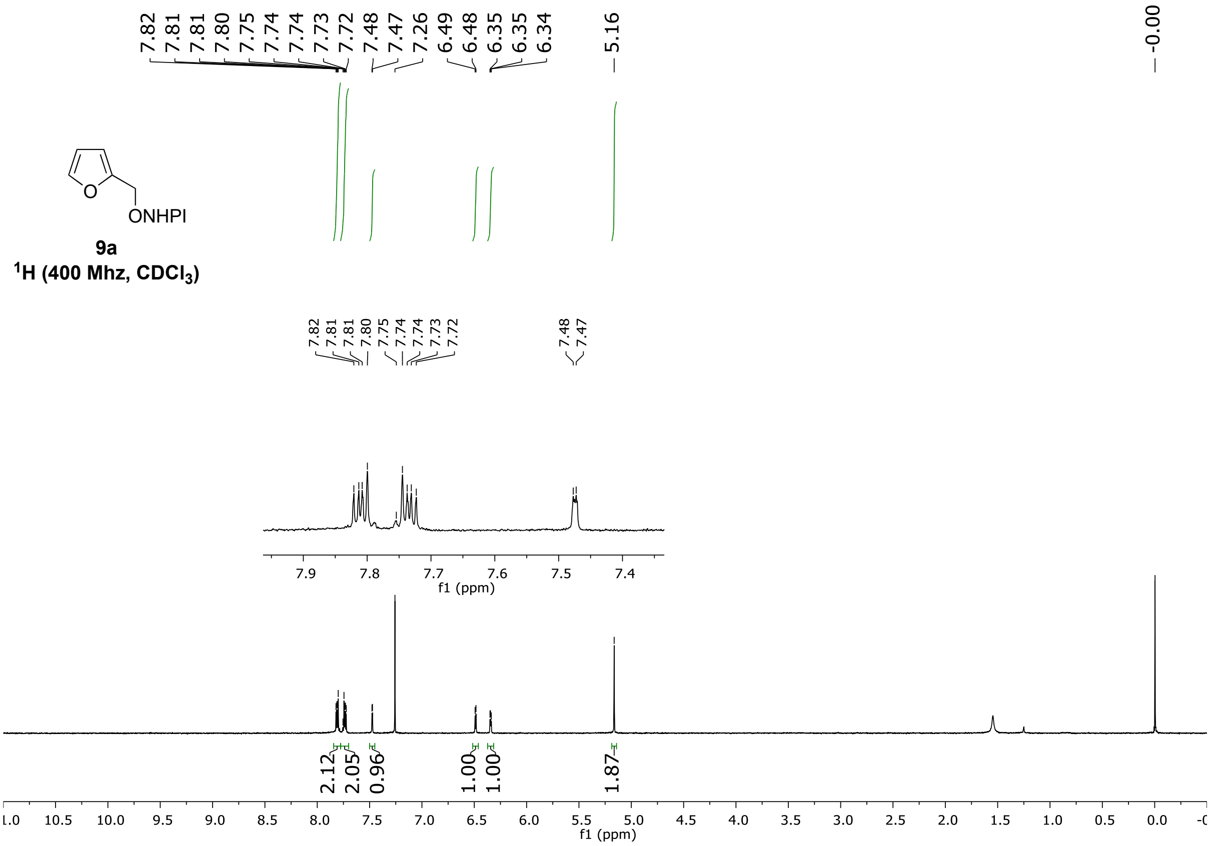

^{1}H NMR Spectrum of 2-(furan-2-ylmethoxy)isoindoline-1,3-dione (**9a**)

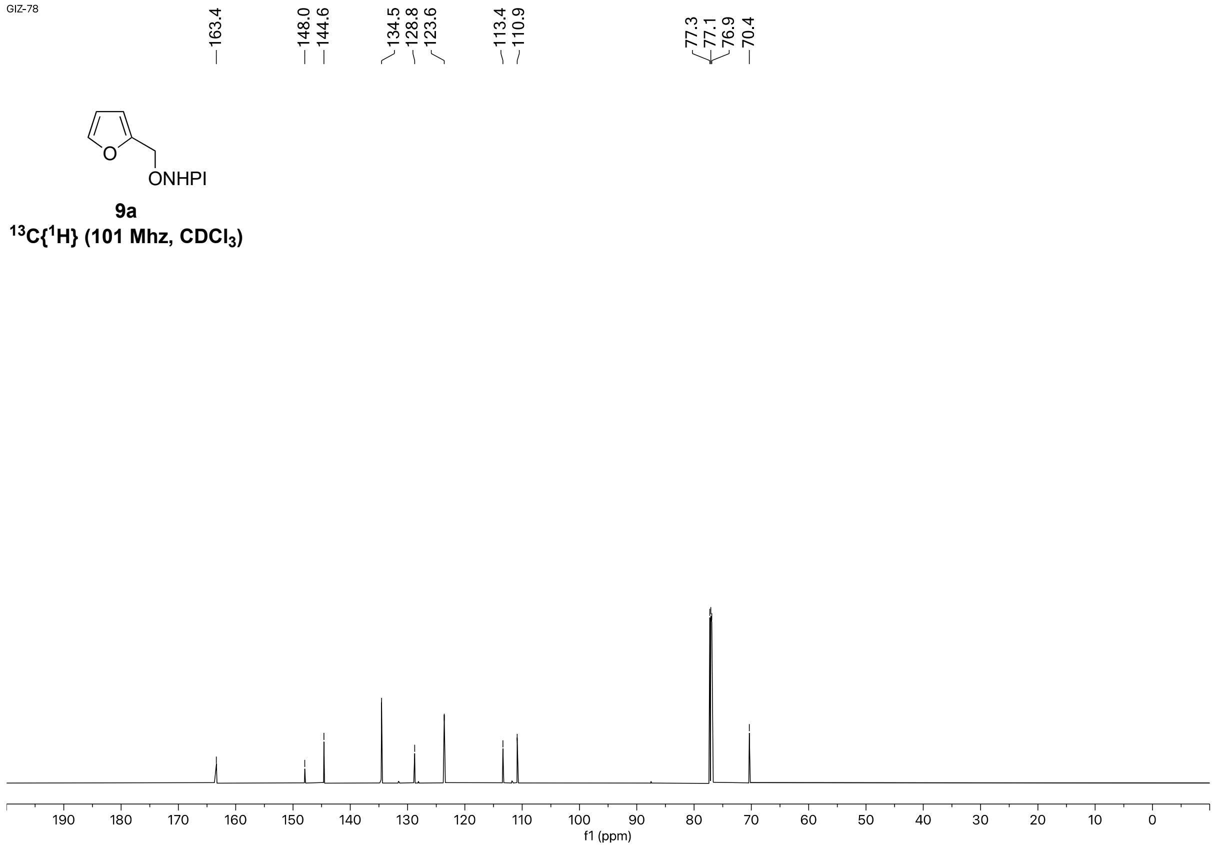

$^{13}C\{^{1}H\}$ NMR Spectrum of 2-(furan-2-ylmethoxy)isoindoline-1,3-dione (**9a**)

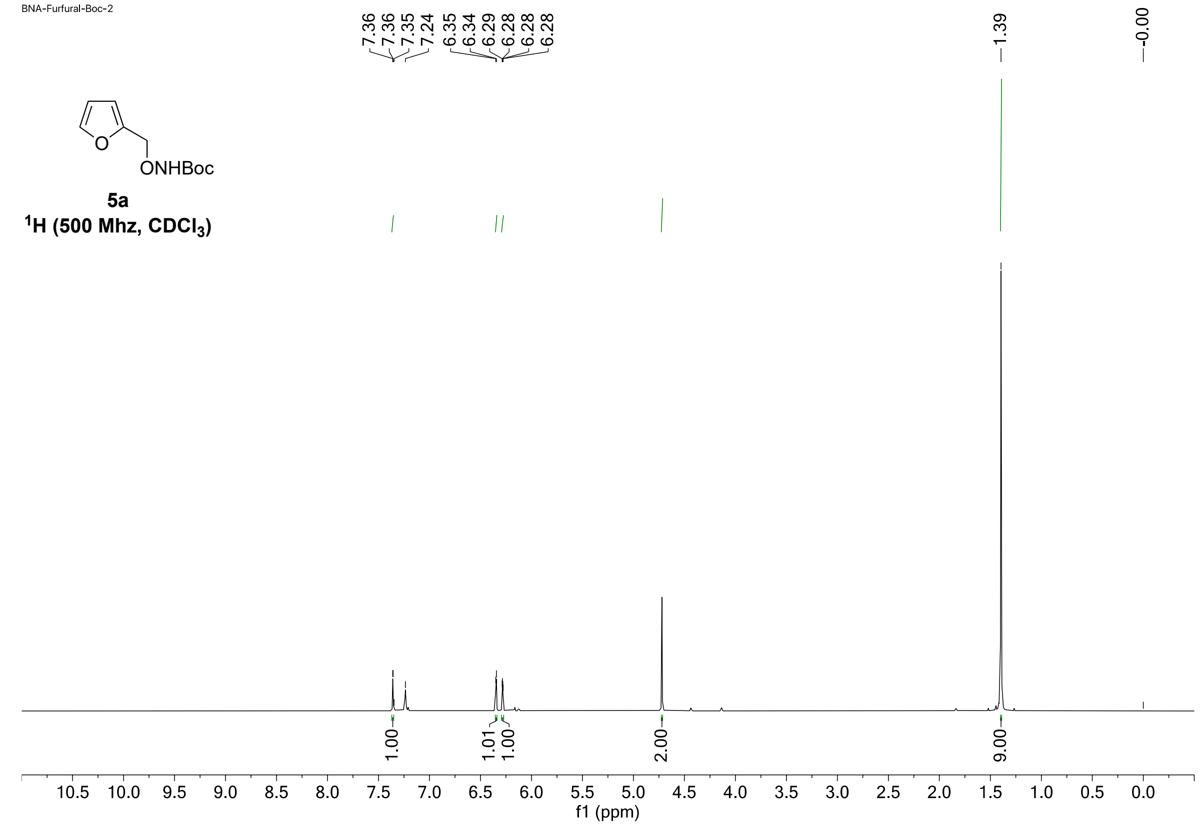

^{1}H NMR Spectrum of *tert*-butyl (furan-2-ylmethoxy)carbamate (**5a**)

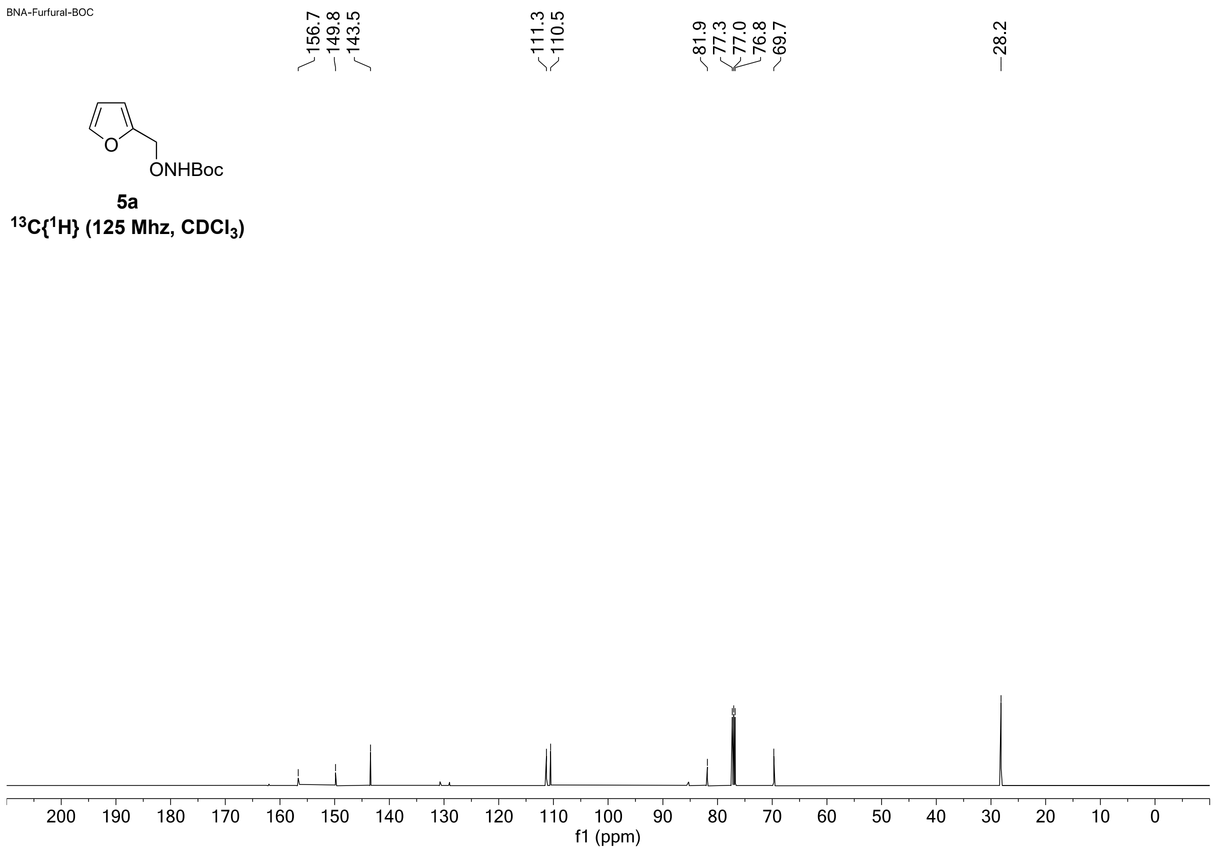

13C{1H} NMR Spectrum of *tert*-butyl (furan-2-ylmethoxy)carbamate (**5a**)

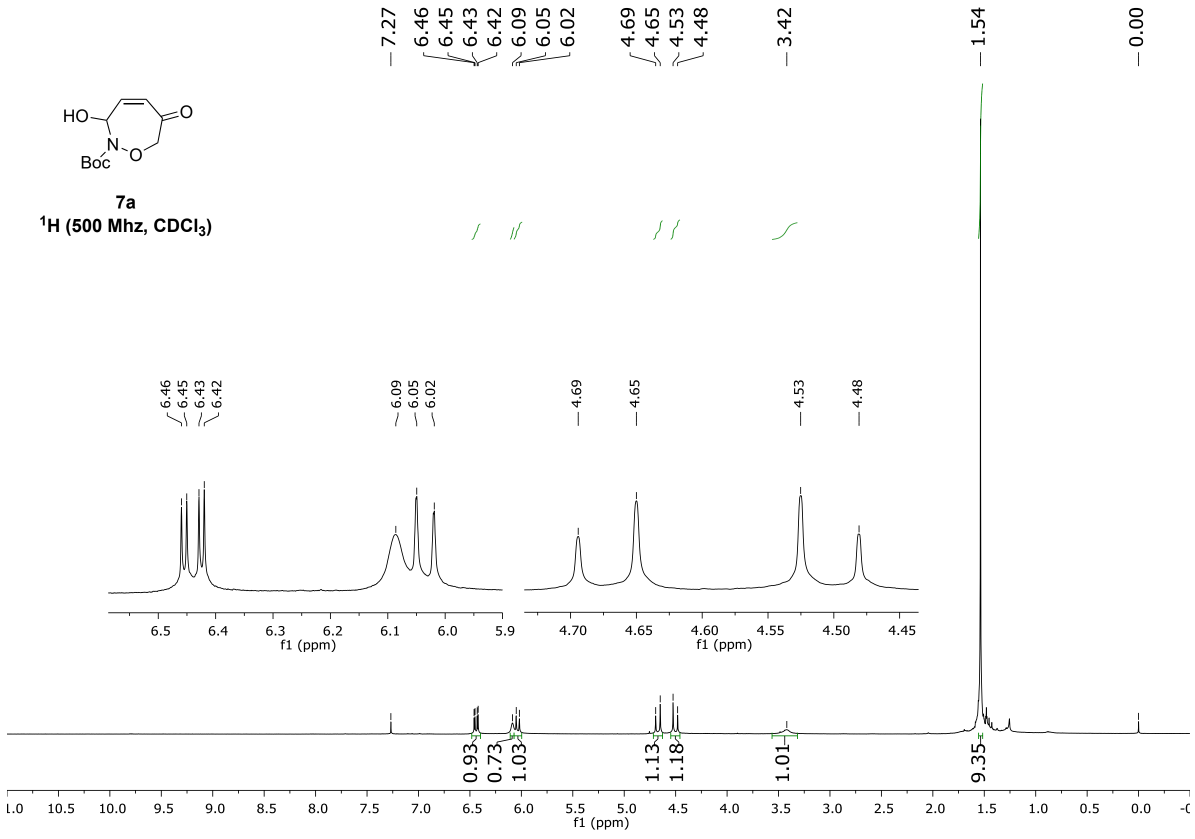

1H NMR Spectrum of *tert*-butyl 3-hydroxy-6-oxo-6,7-dihydro-1,2-oxazepine-2(*3H*)-carboxylate (**7a**)

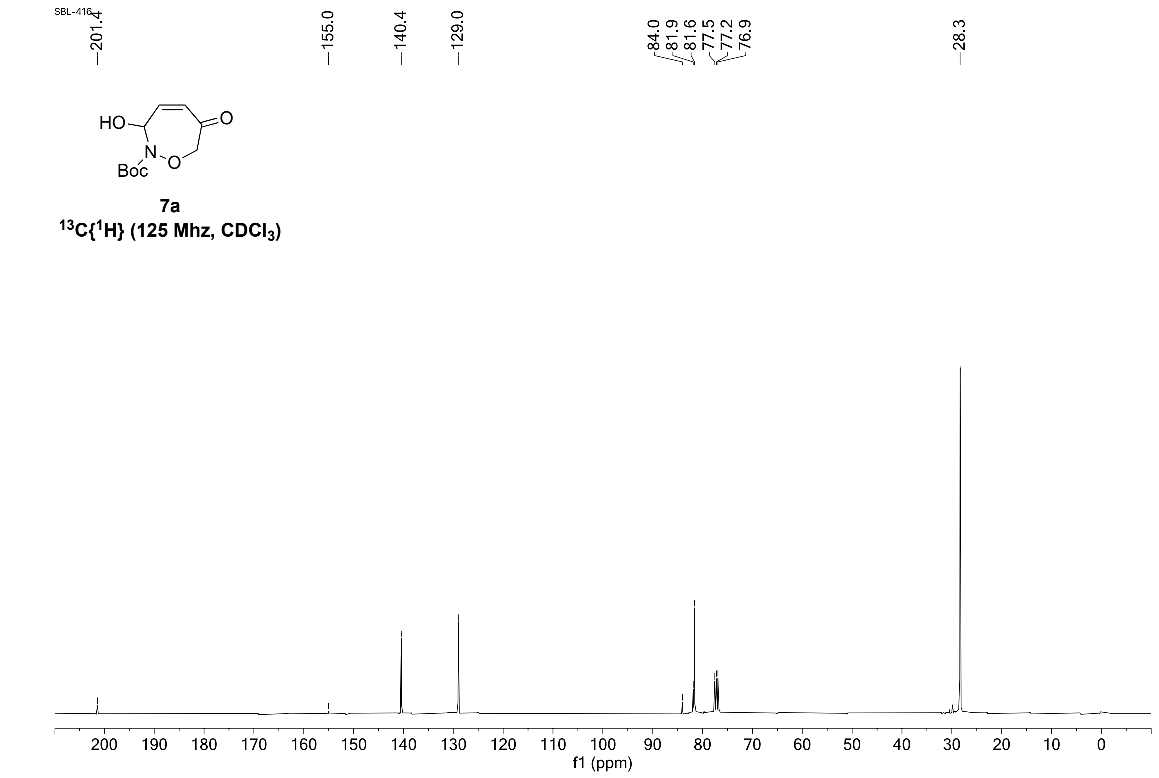

$^{13}C\{^1H\}$ NMR Spectrum of *tert*-butyl 3-hydroxy-6-oxo-6,7-dihydro-1,2-oxazepine-2(*3H*)-carboxylate (**7a**)

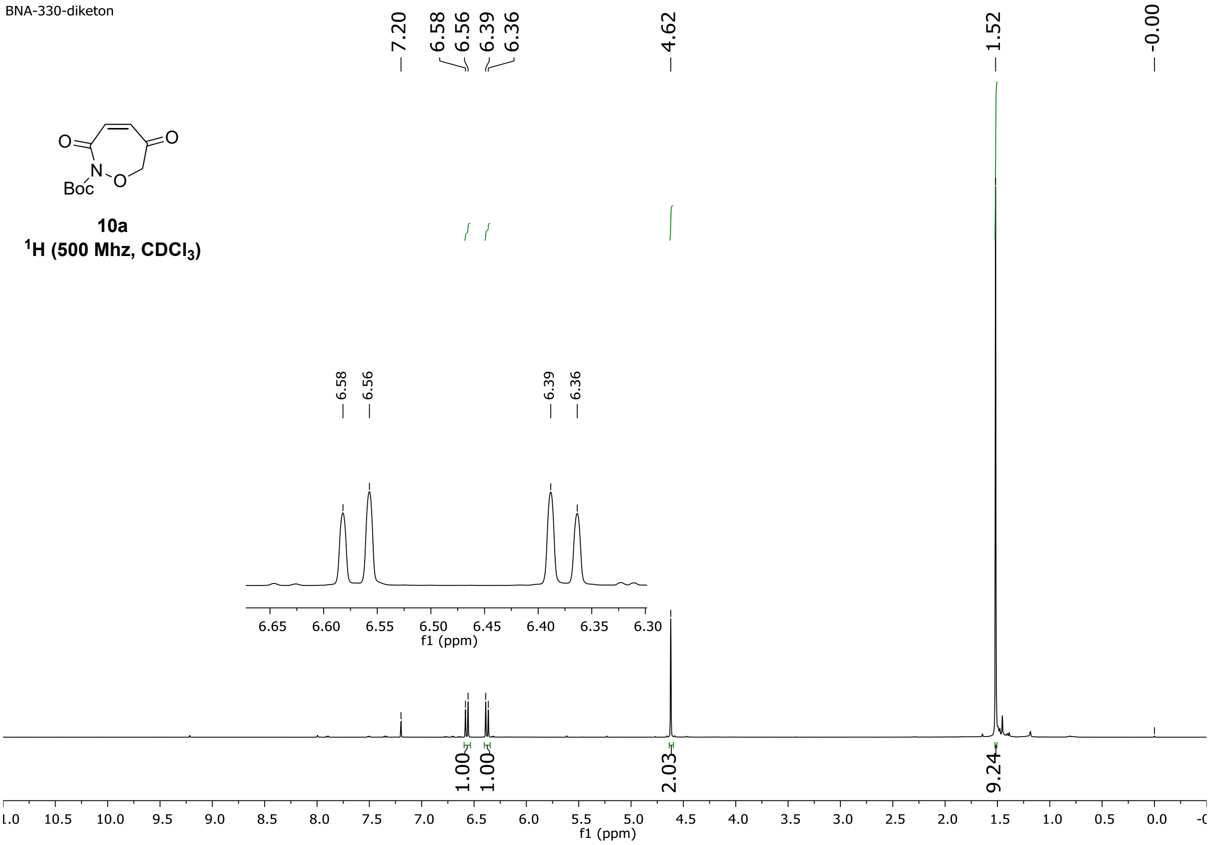

^1H NMR Spectrum of *tert*-butyl 3,6-dioxo-6,7-dihydro-1,2-oxazepine-2(*3H*)-carboxylate (**10a**)

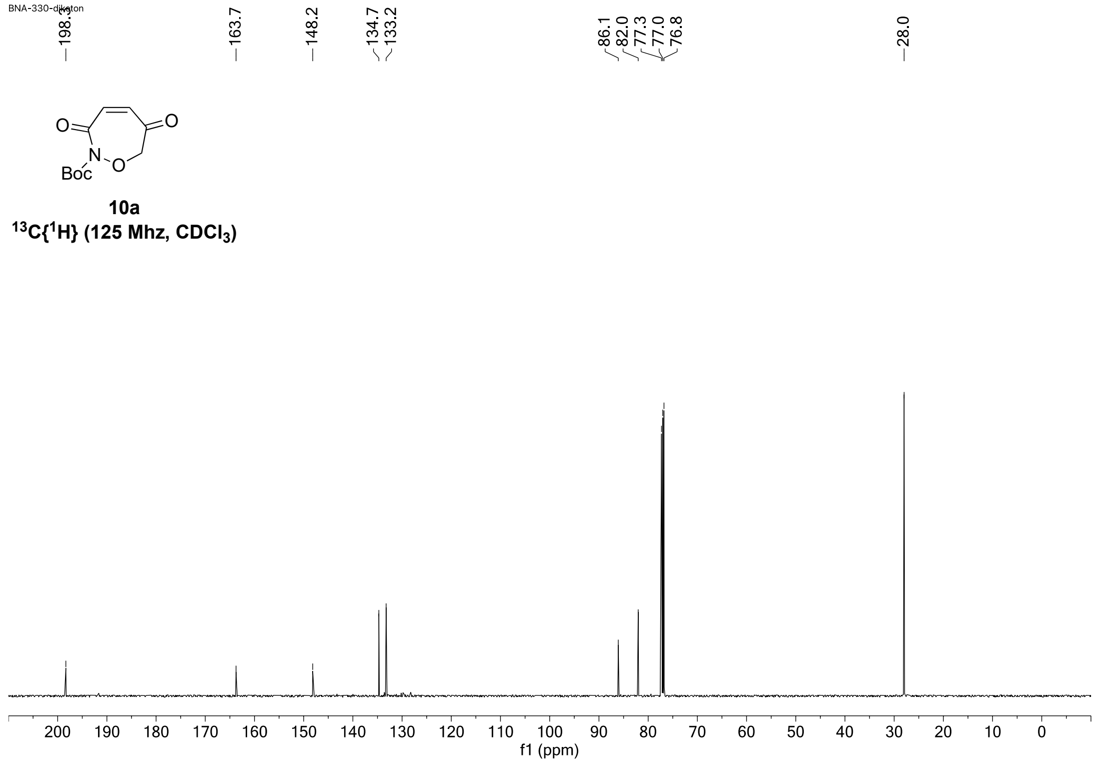

$^{13}C\{^1H\}$ NMR Spectrum of *tert*-butyl 3,6-dioxo-6,7-dihydro-1,2-oxazepine-2(*3H*)-carboxylate (**10a**)

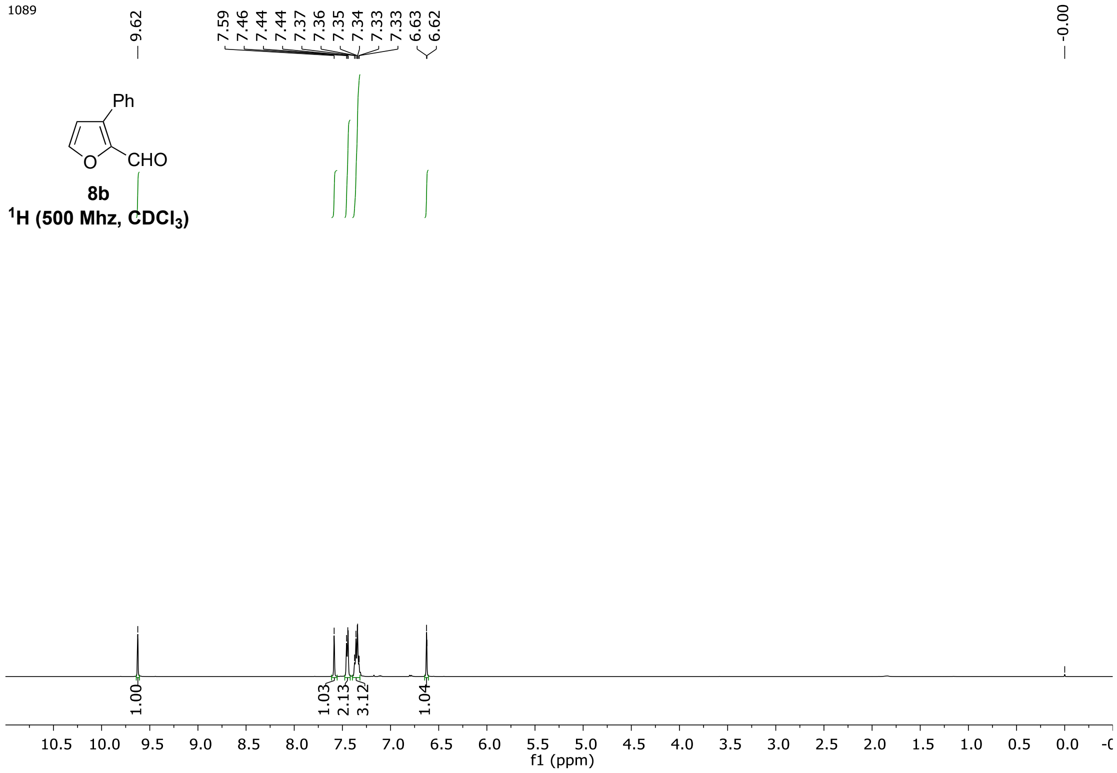

^1H NMR Spectrum of 3-phenylfuran-2-carbaldehyde (**8b**)

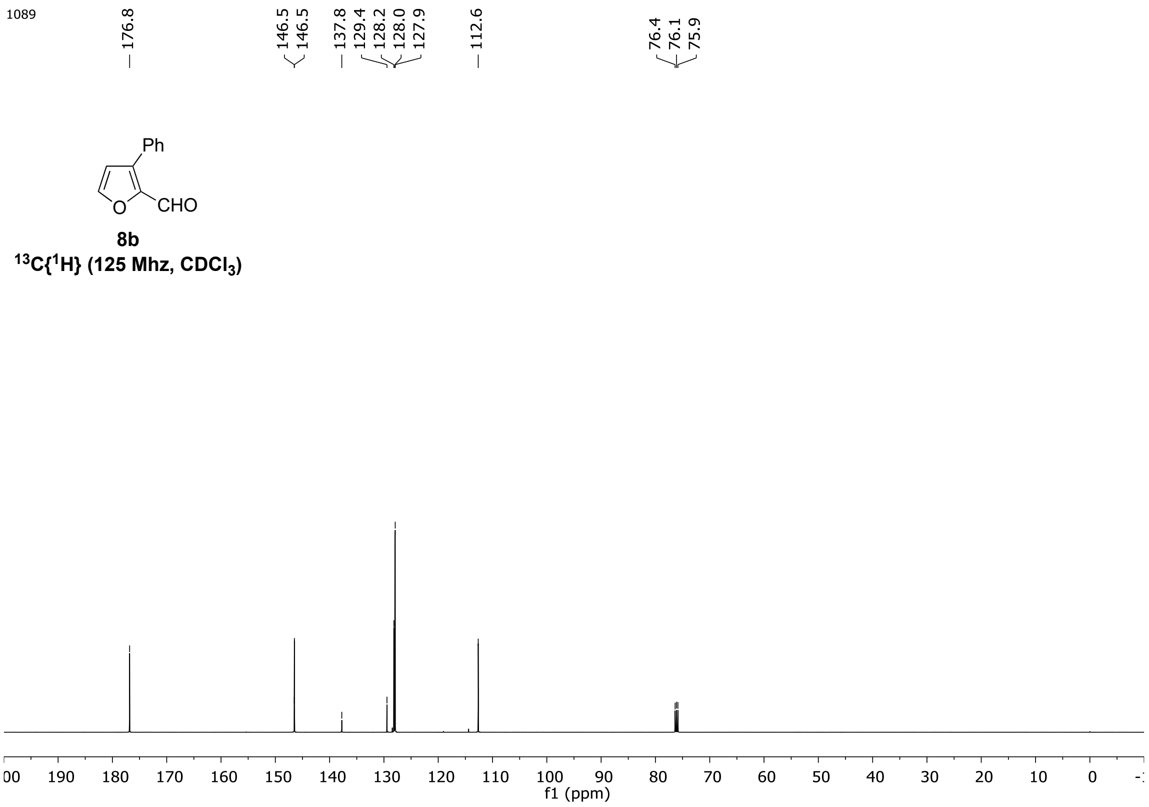

$^{13}C\{^1H\}$ NMR Spectrum of 3-phenylfuran-2-carbaldehyde (**8b**)

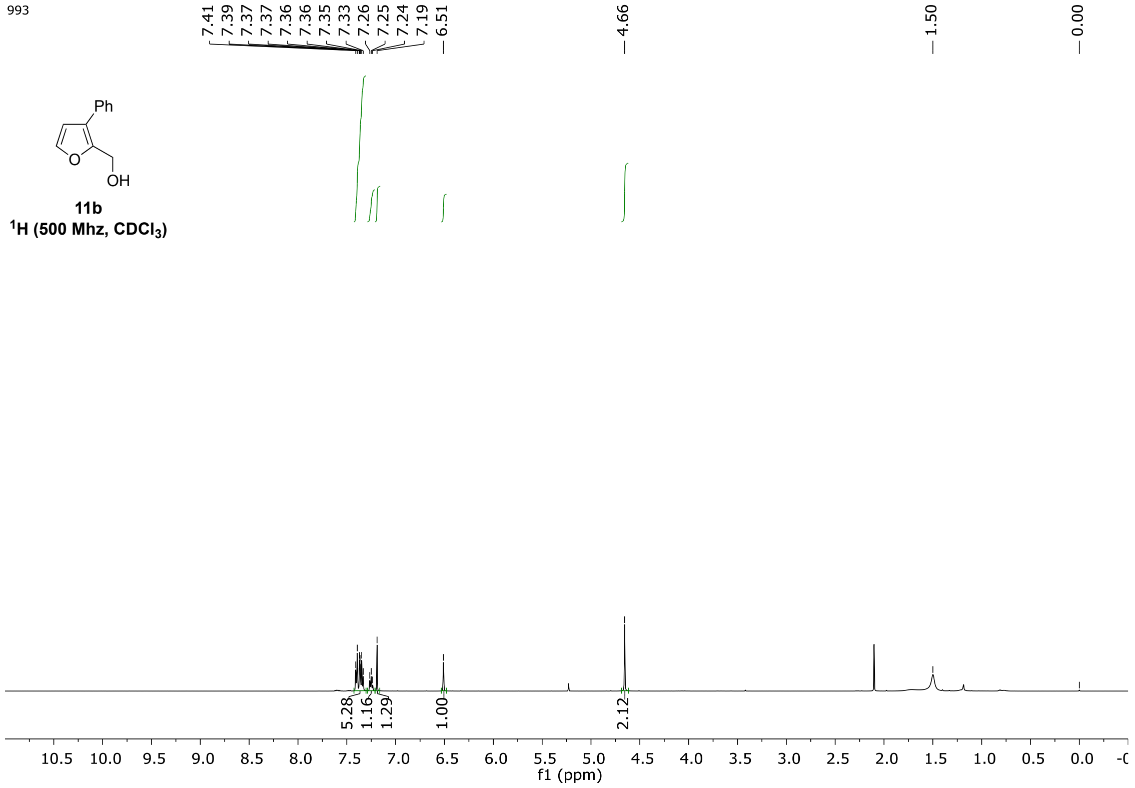

^1H NMR Spectrum of (3-phenylfuran-2-yl)methanol (**11b**)

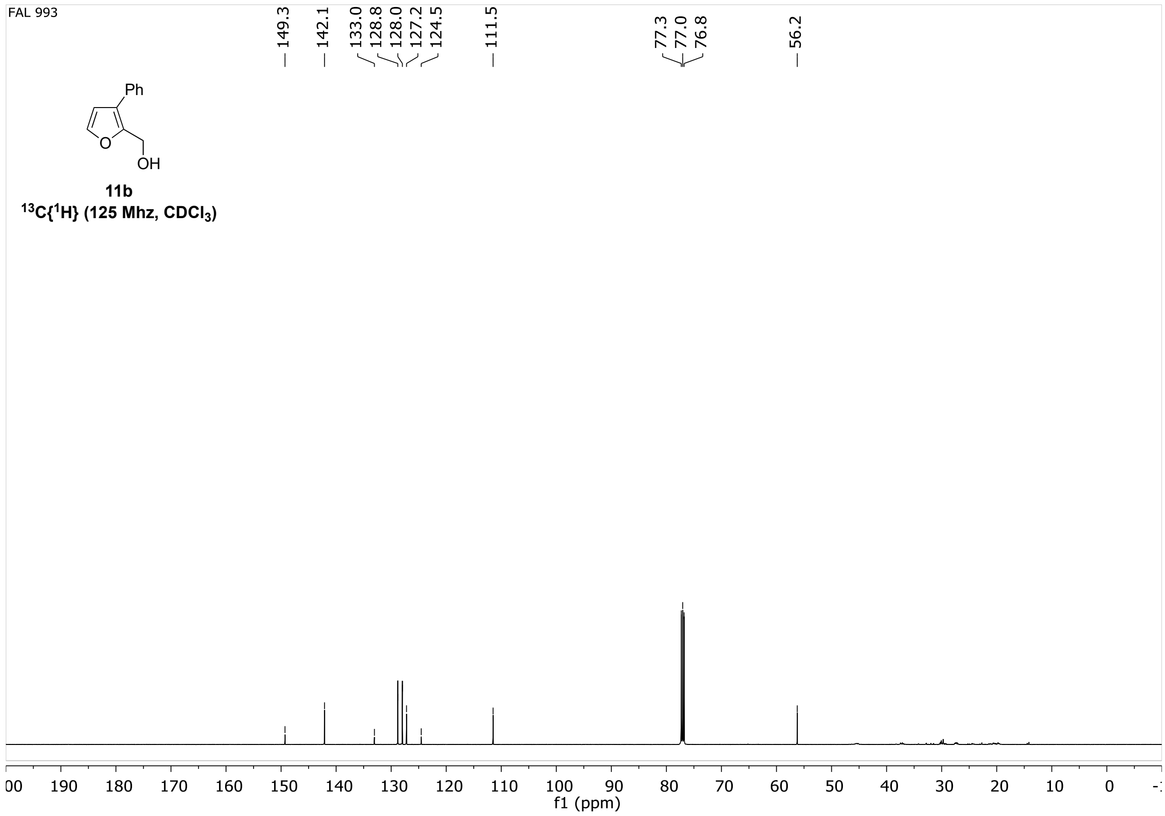

$^{13}C\{^1H\}$ NMR Spectrum of (3-phenylfuran-2-yl)methanol (**11b**)

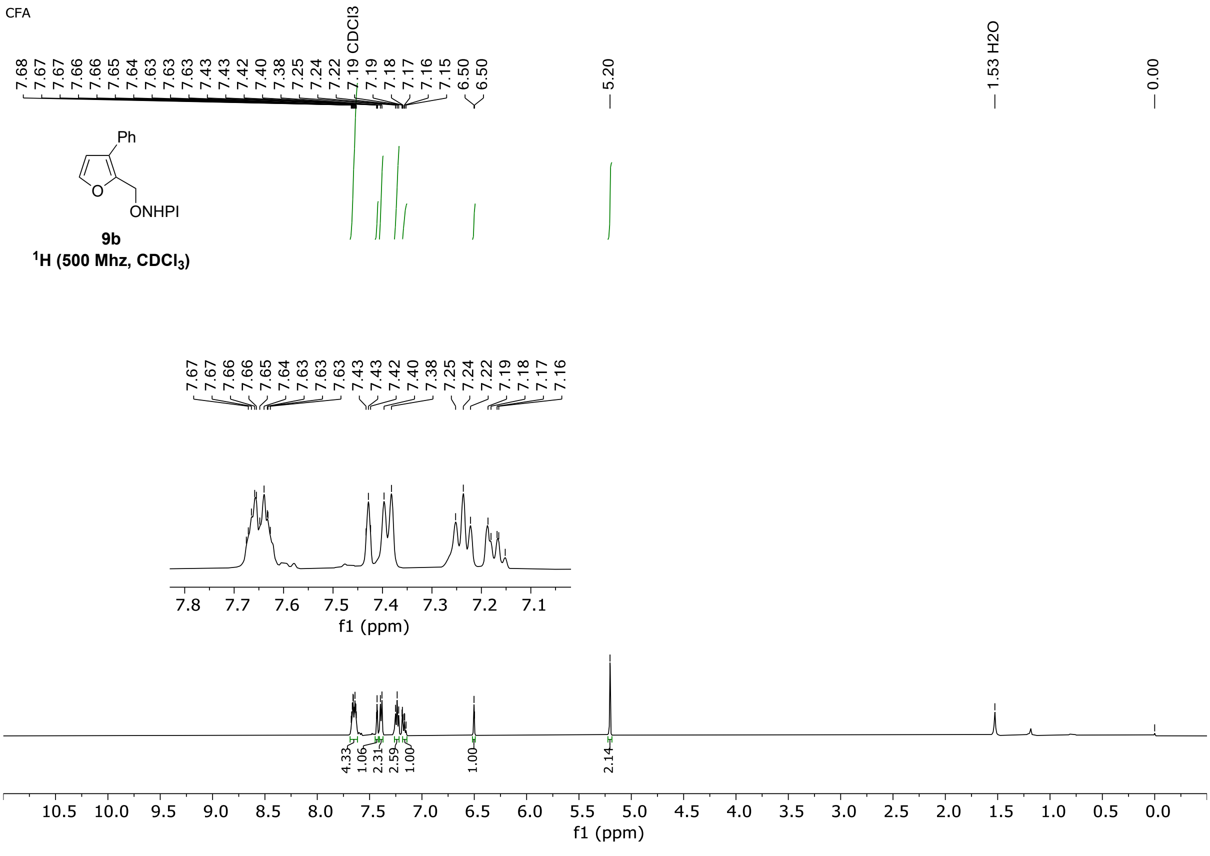

^1H NMR Spectrum of 2-((3-phenylfuran-2-yl)methoxy)isoindoline-1,3-dione (**9b**)

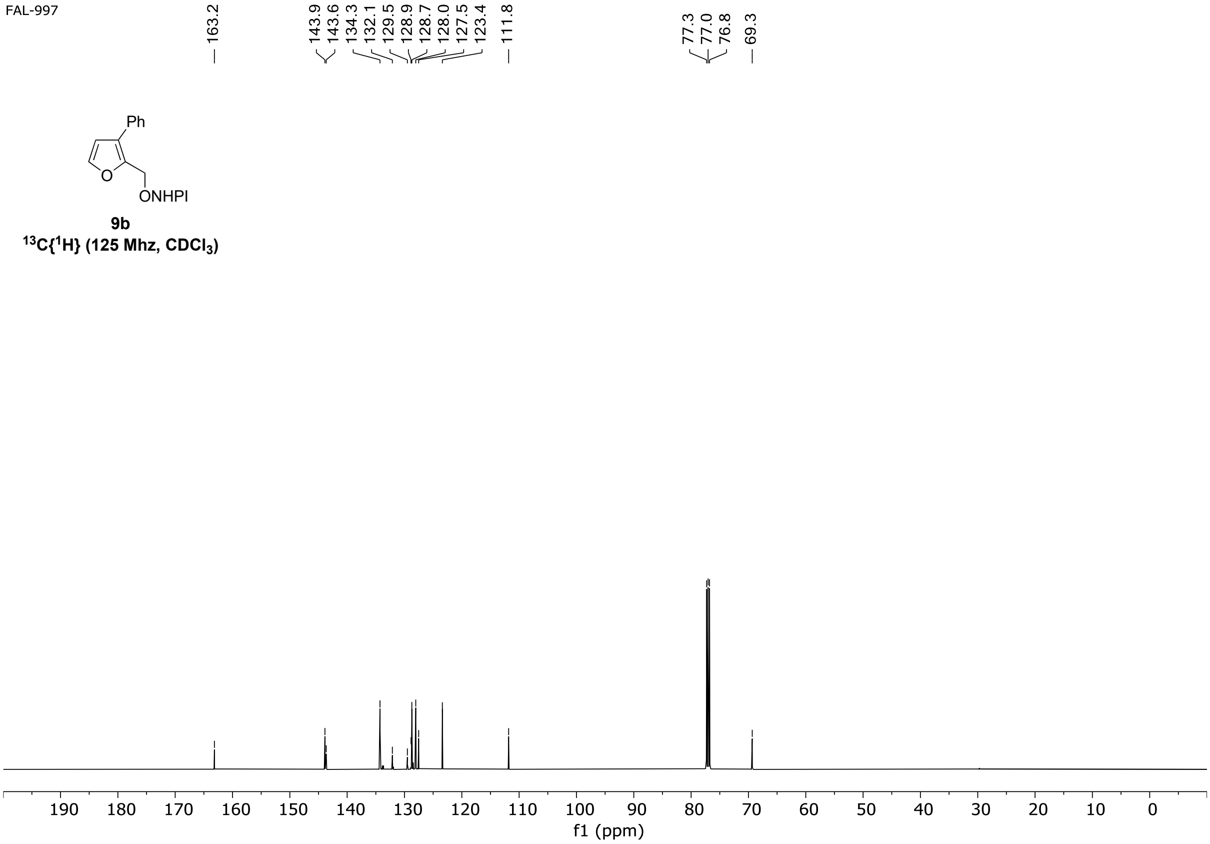

$^{13}C\{^{1}H\}$ NMR Spectrum of 2-((3-phenylfuran-2-yl)methoxy)isoindoline-1,3-dione (**9b**)

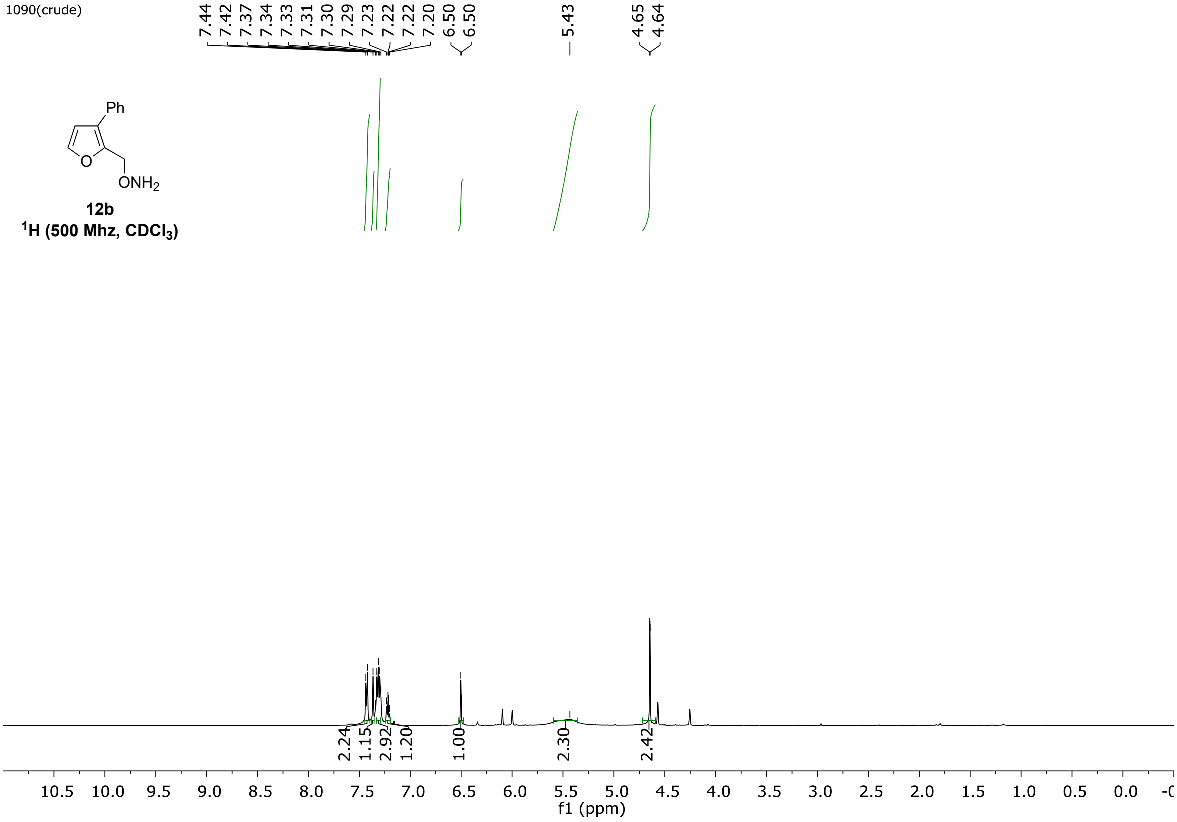

^{1}H NMR Spectrum of *O*-((3-phenylfuran-2-yl)methyl)hydroxylamine (**12b**)

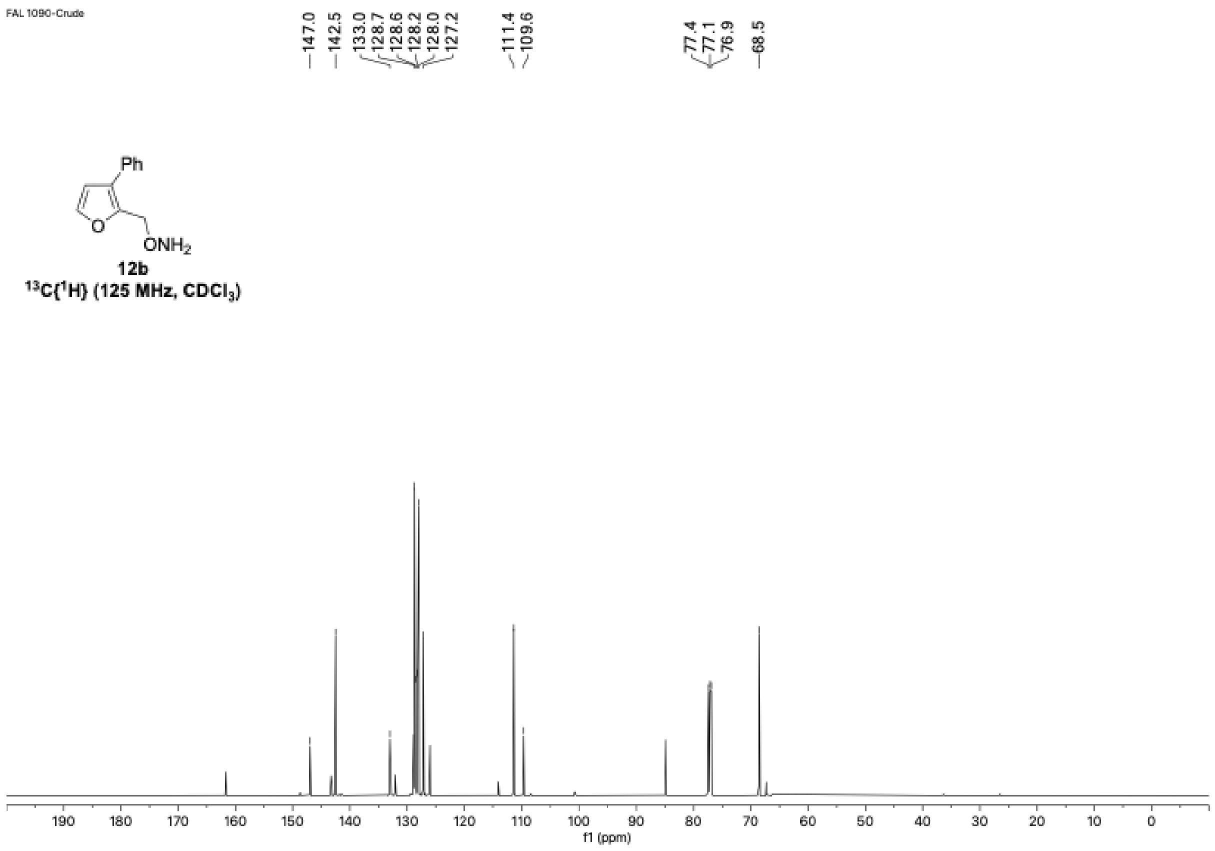

$^{13}C\{^{1}H\}$ NMR Spectrum of *O*-((3-phenylfuran-2-yl)methyl)hydroxylamine (**12b**)

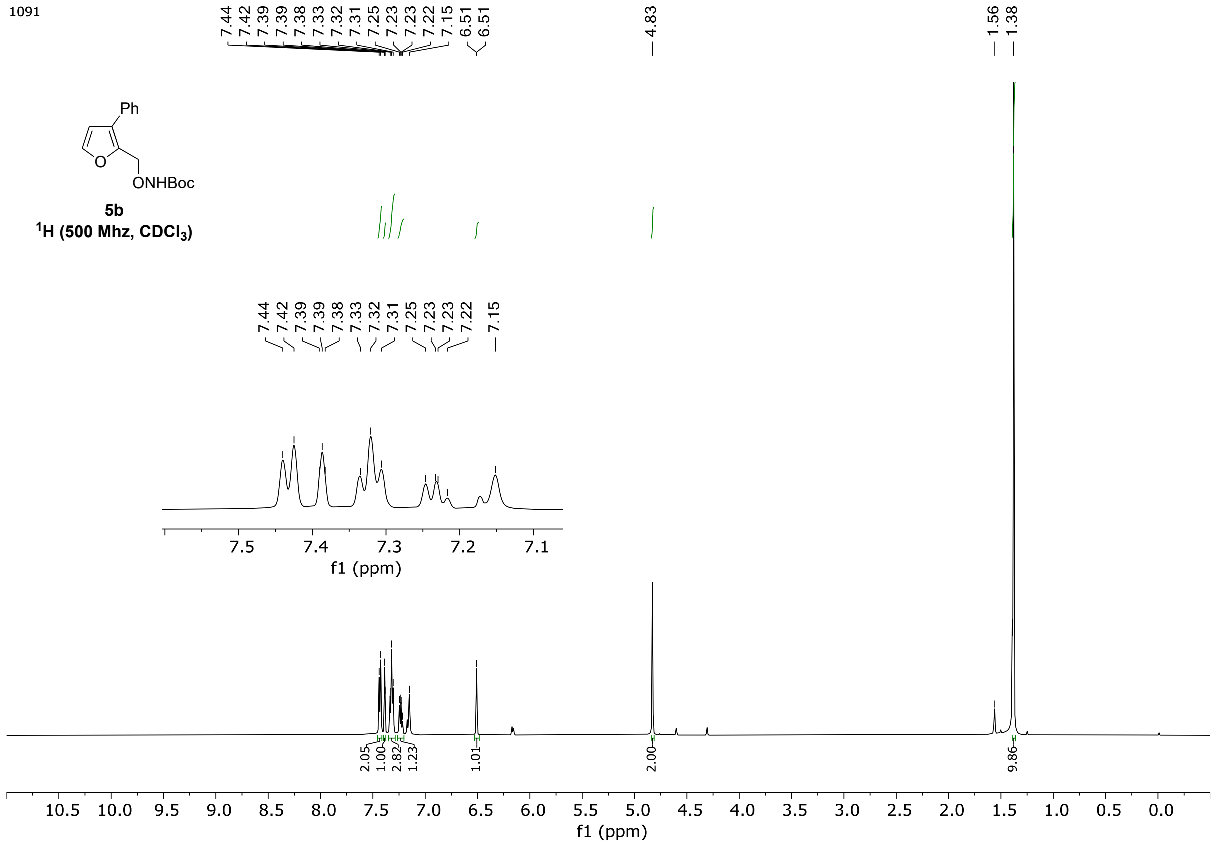

^{1}H NMR Spectrum of *tert*-butyl ((3-phenylfuran-2-yl)methoxy)carbamate (**5b**)

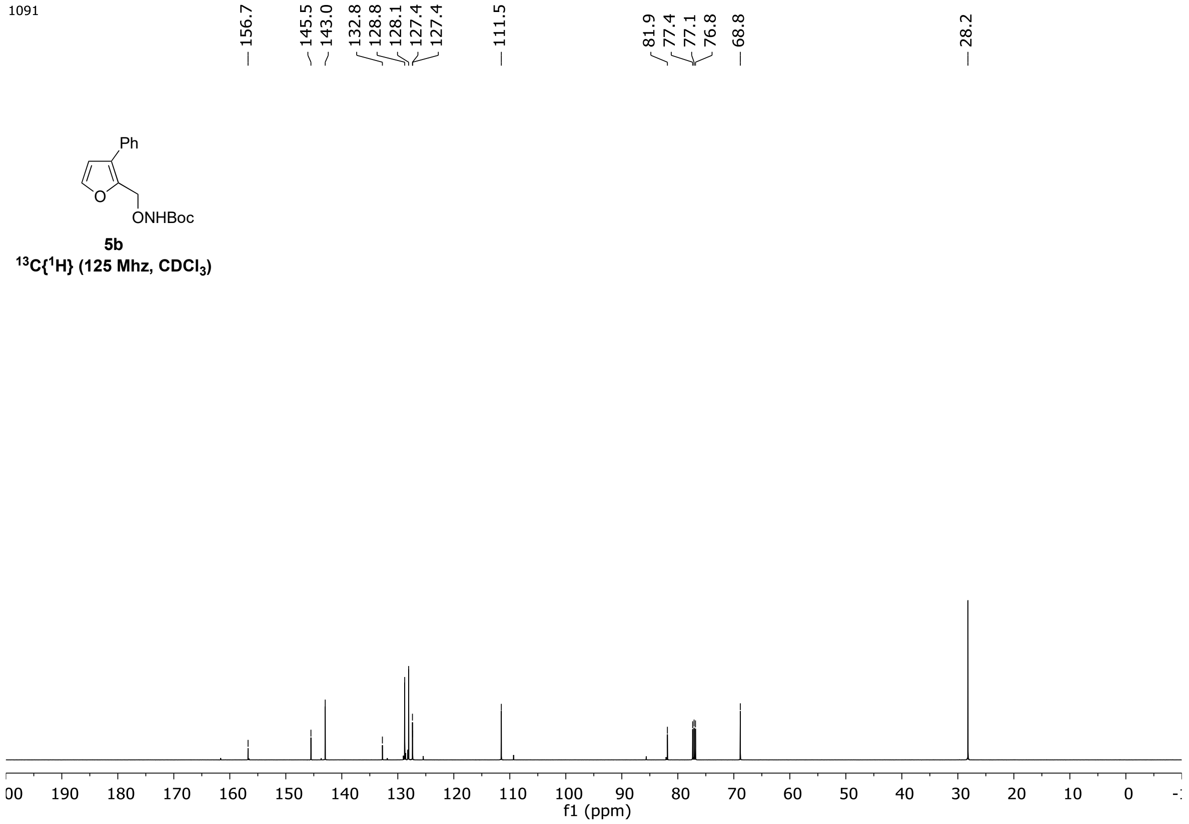

$^{13}C\{^{1}H\}$ NMR Spectrum of *tert*-butyl ((3-phenylfuran-2-yl)methoxy)carbamate (**5b**)

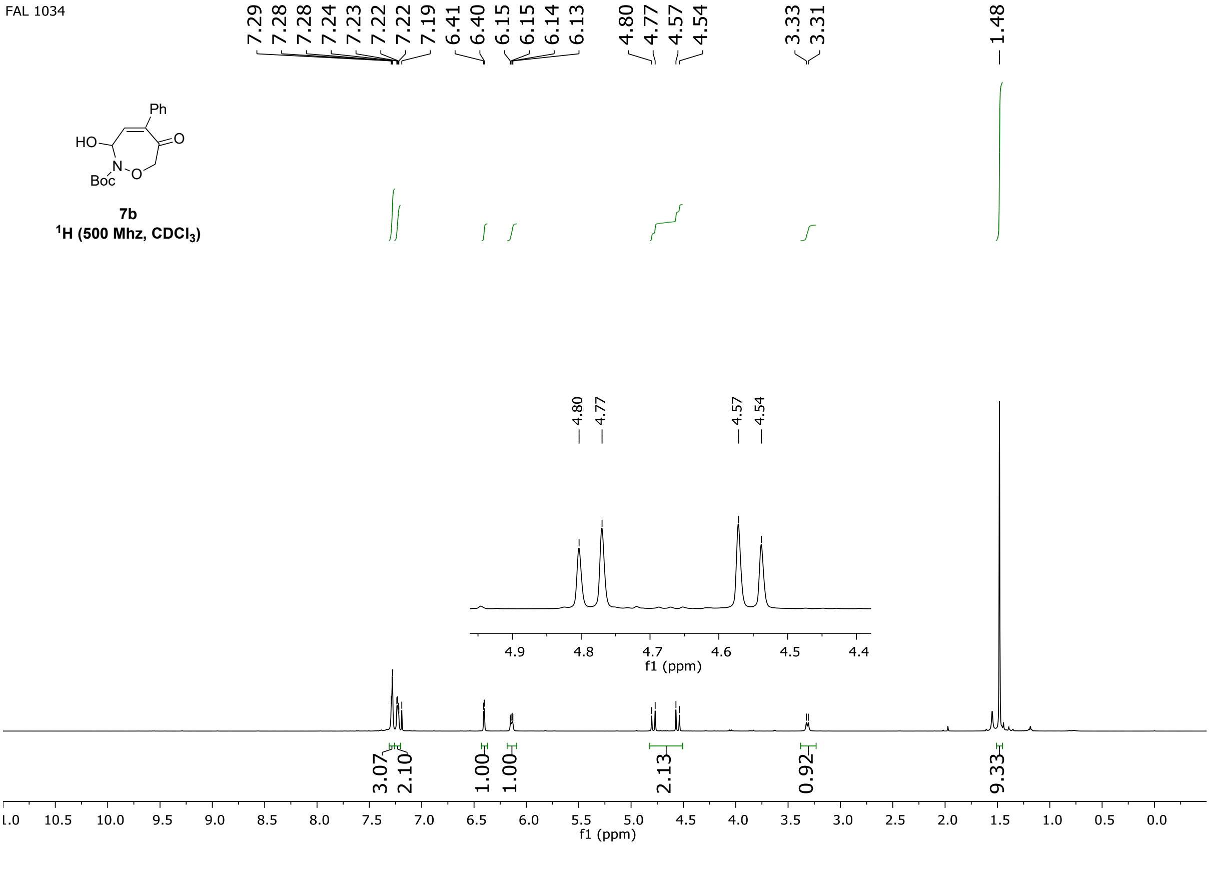

^{1}H NMR Spectrum of *tert*-butyl 3-hydroxy-6-oxo-5-phenyl-6,7-dihydro-1,2-oxazepine-2(*3H*)-carboxylate (**7b**)

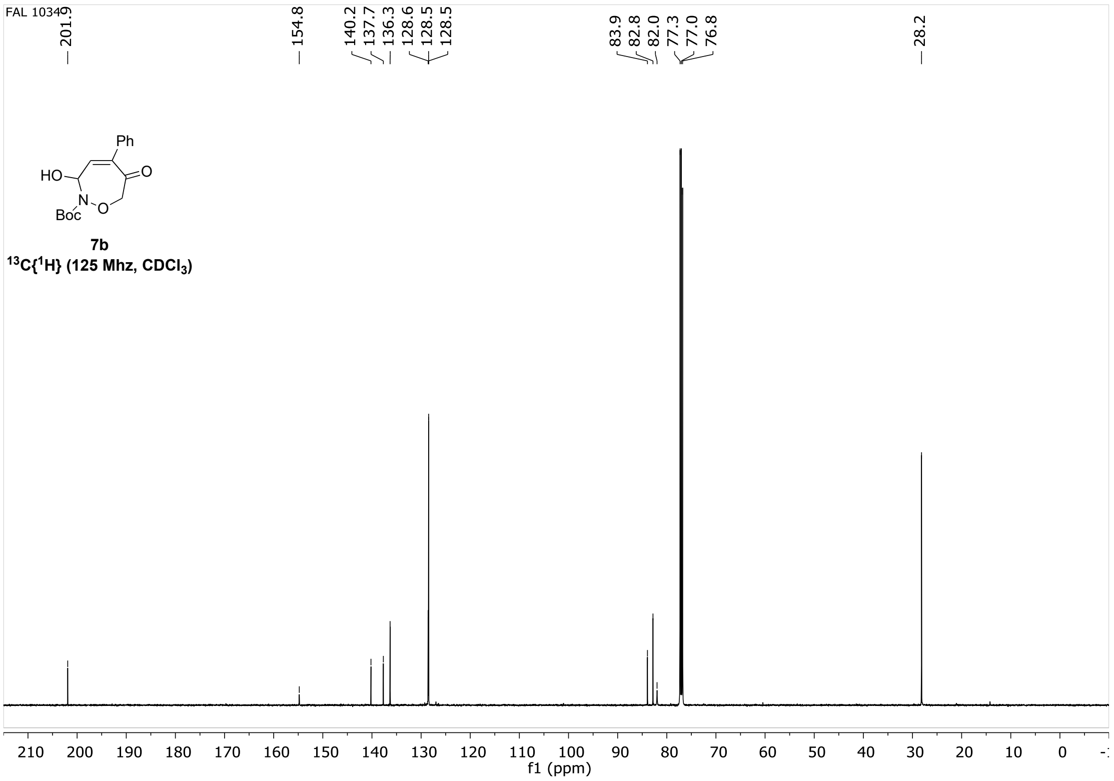

$^{13}C\{^1H\}$ NMR Spectrum of *tert*-butyl 3-hydroxy-6-oxo-5-phenyl-6,7-dihydro-1,2-oxazepine-2(*3H*)-carboxylate (**7b**)

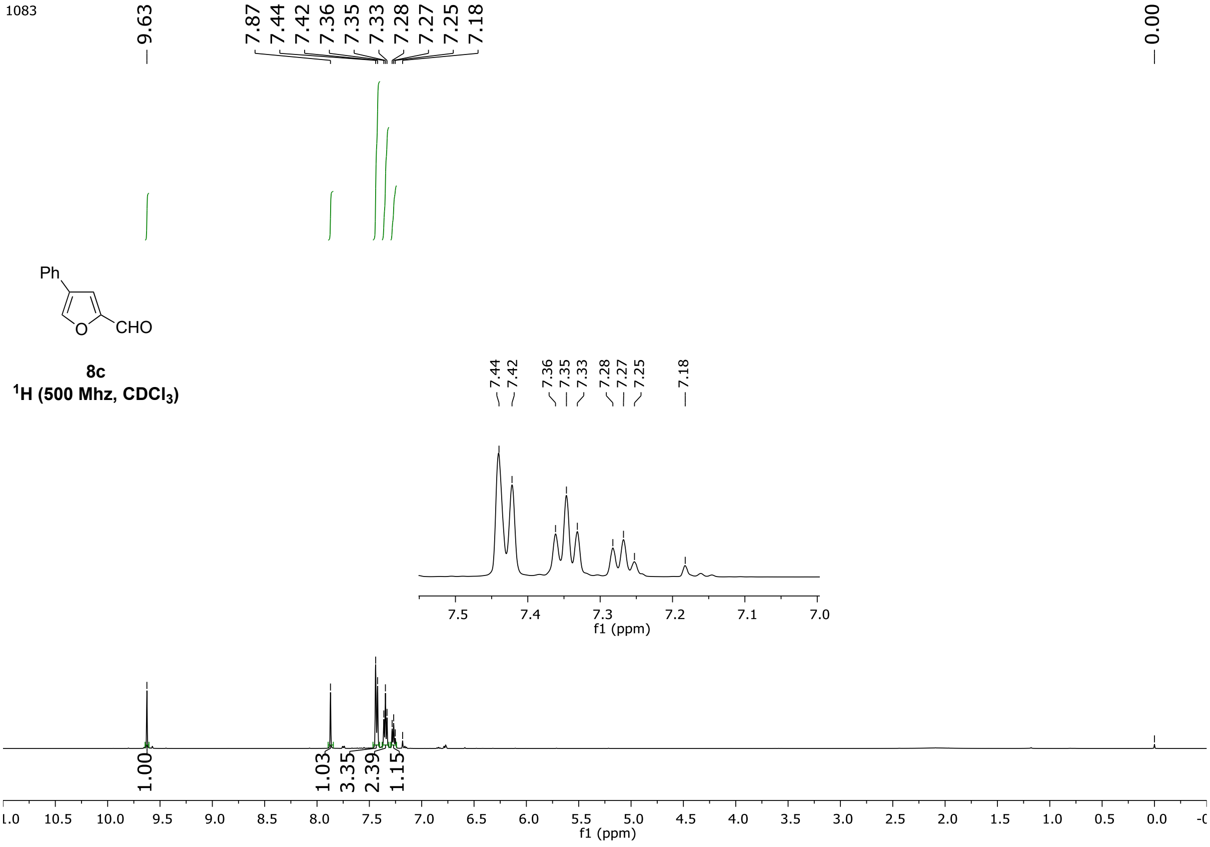

^1H NMR Spectrum of 4-phenylfuran-2-carbaldehyde (**8c**)

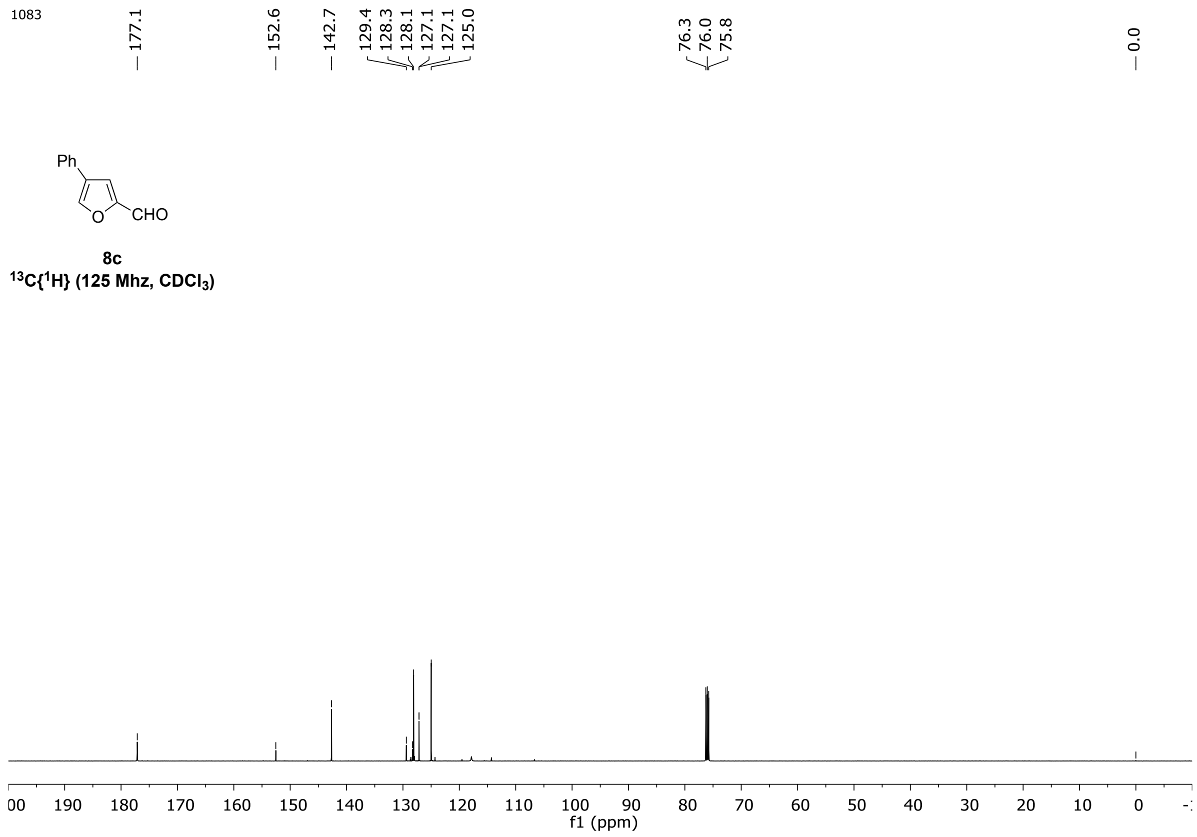

$^{13}C\{^{1}H\}$ NMR Spectrum of 4-phenylfuran-2-carbaldehyde (**8c**)

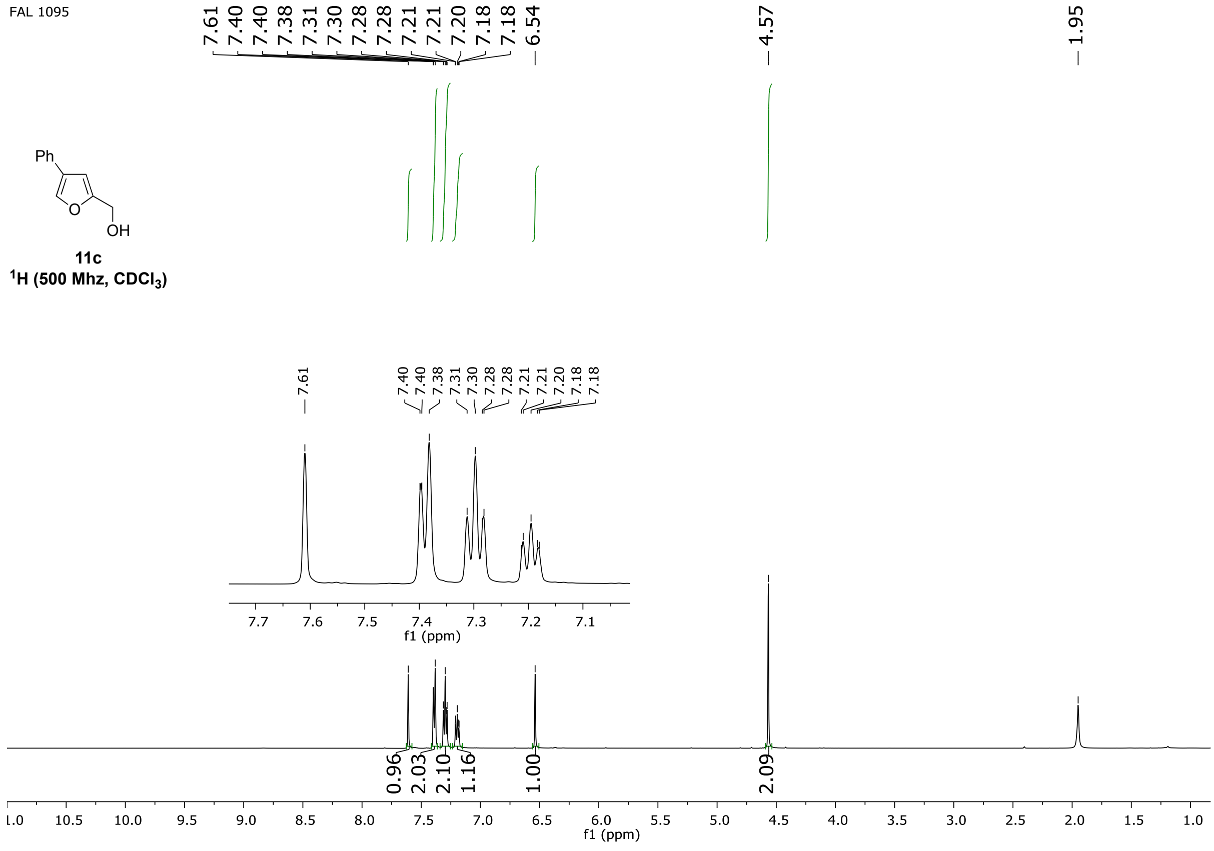

^{1}H NMR Spectrum of (4-phenylfuran-2-yl)methanol (**11c**)

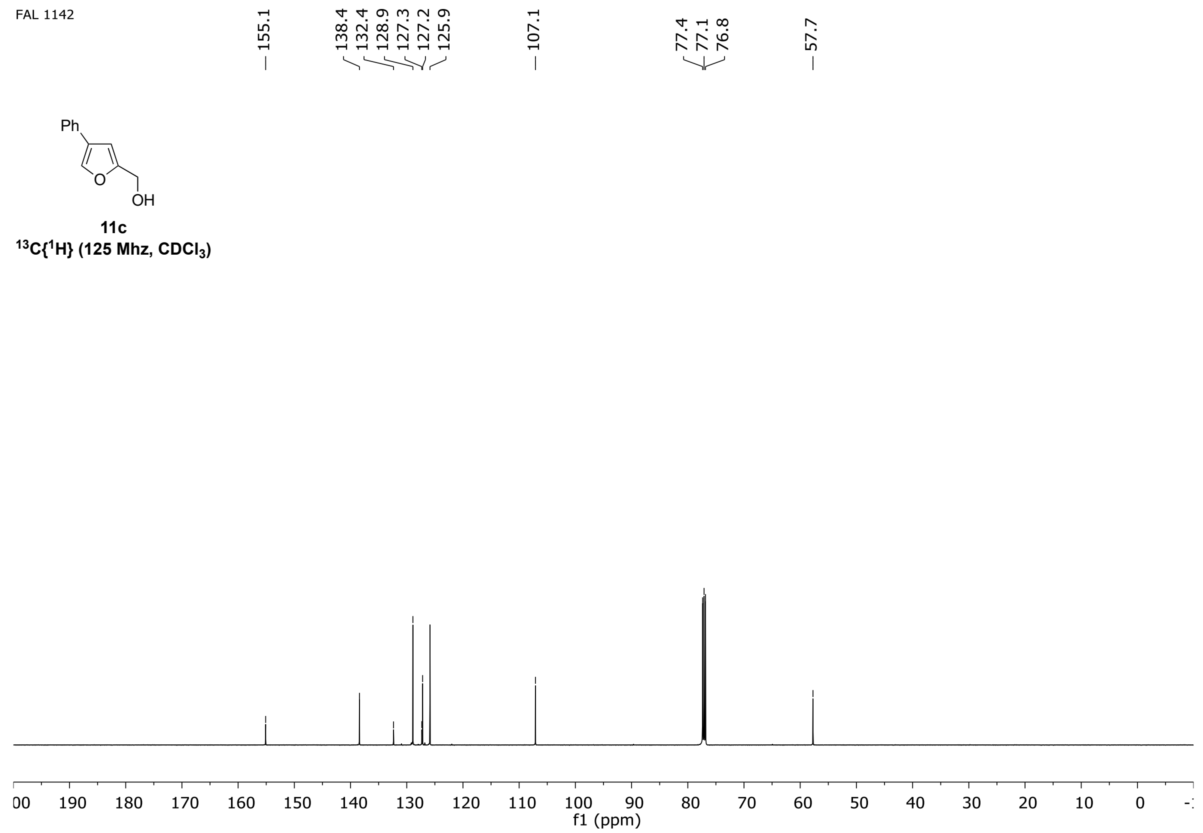

$^{13}C\{^1H\}$ NMR Spectrum of (4-phenylfuran-2-yl)methanol (**11c**)

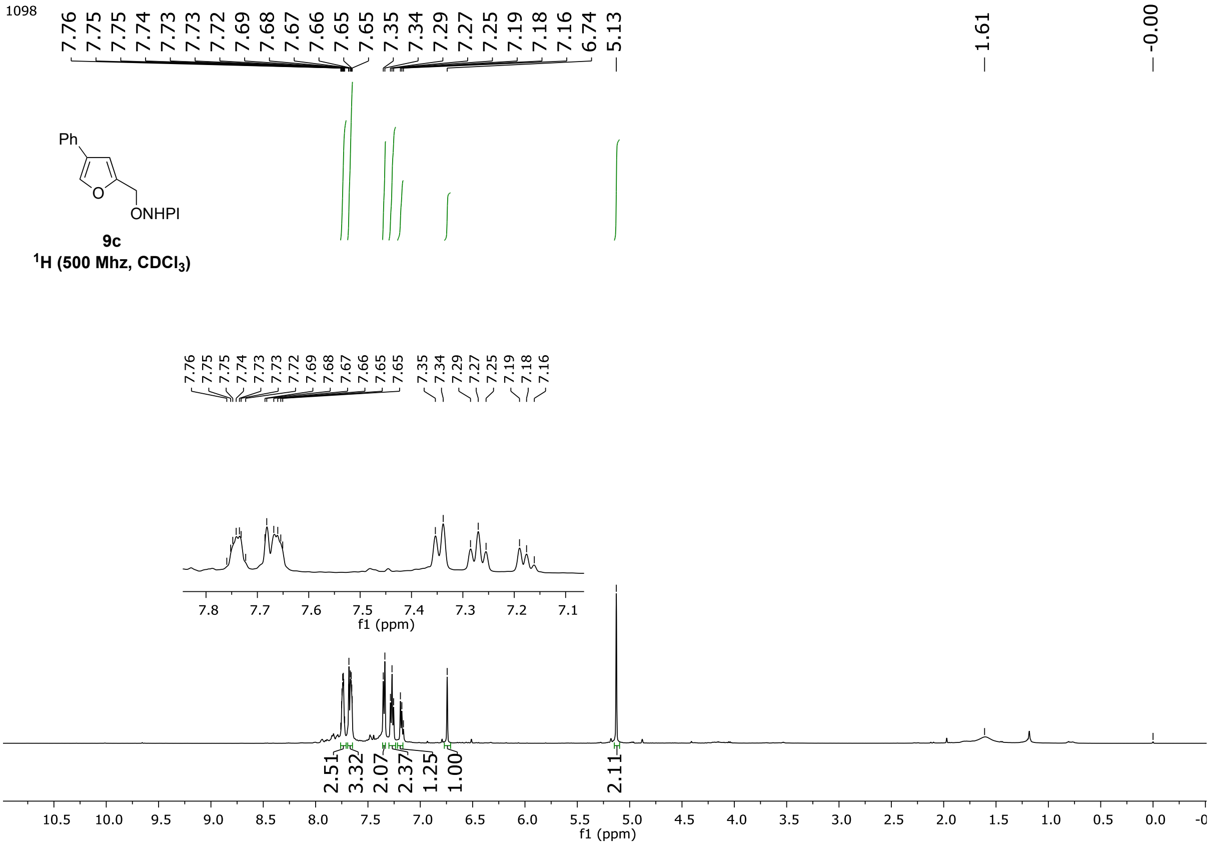

^1H NMR Spectrum of 2-((4-phenylfuran-2-yl)methoxy)isoindoline-1,3-dione (**9c**)

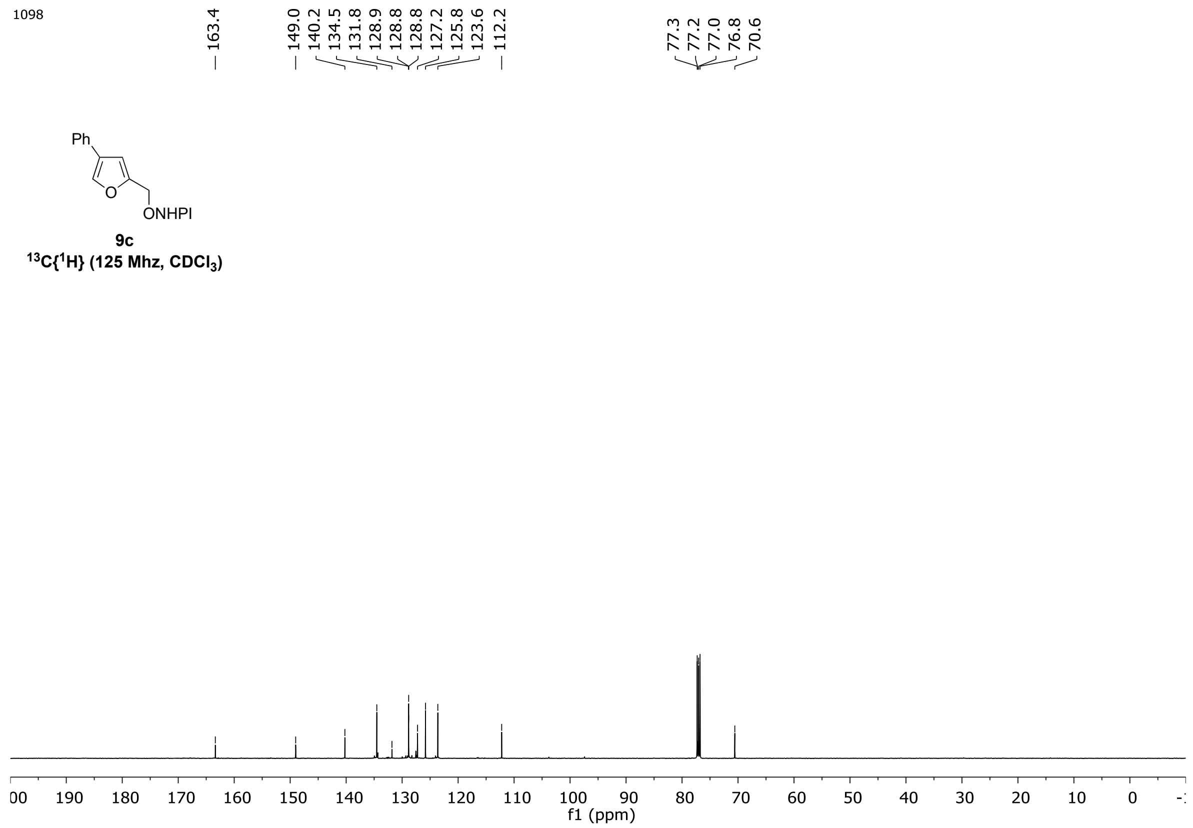

$^{13}C\{^{1}H\}$ NMR Spectrum of 2-((4-phenylfuran-2-yl)methoxy)isoindoline-1,3-dione (**9c**)

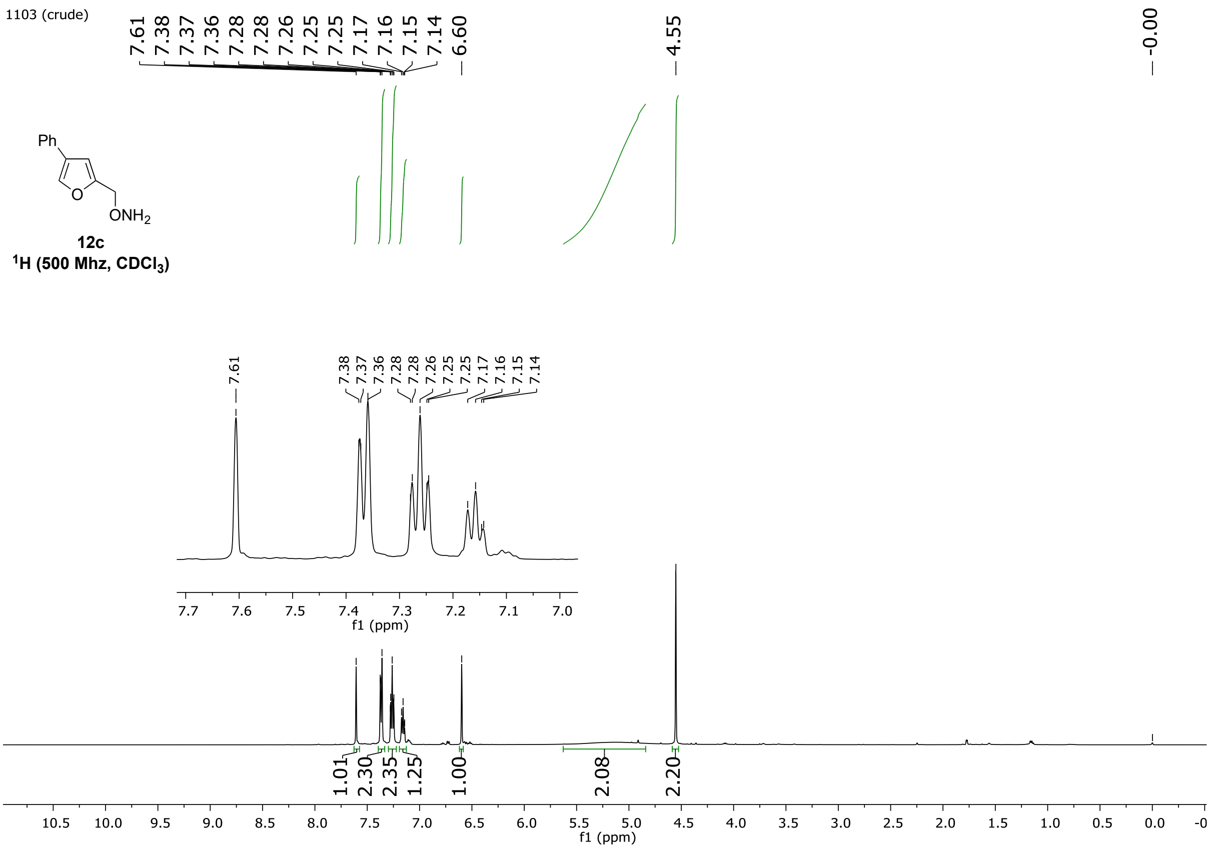

^{1}H NMR Spectrum of *O*-((4-phenylfuran-2-yl)methyl)hydroxylamine (**12c**)

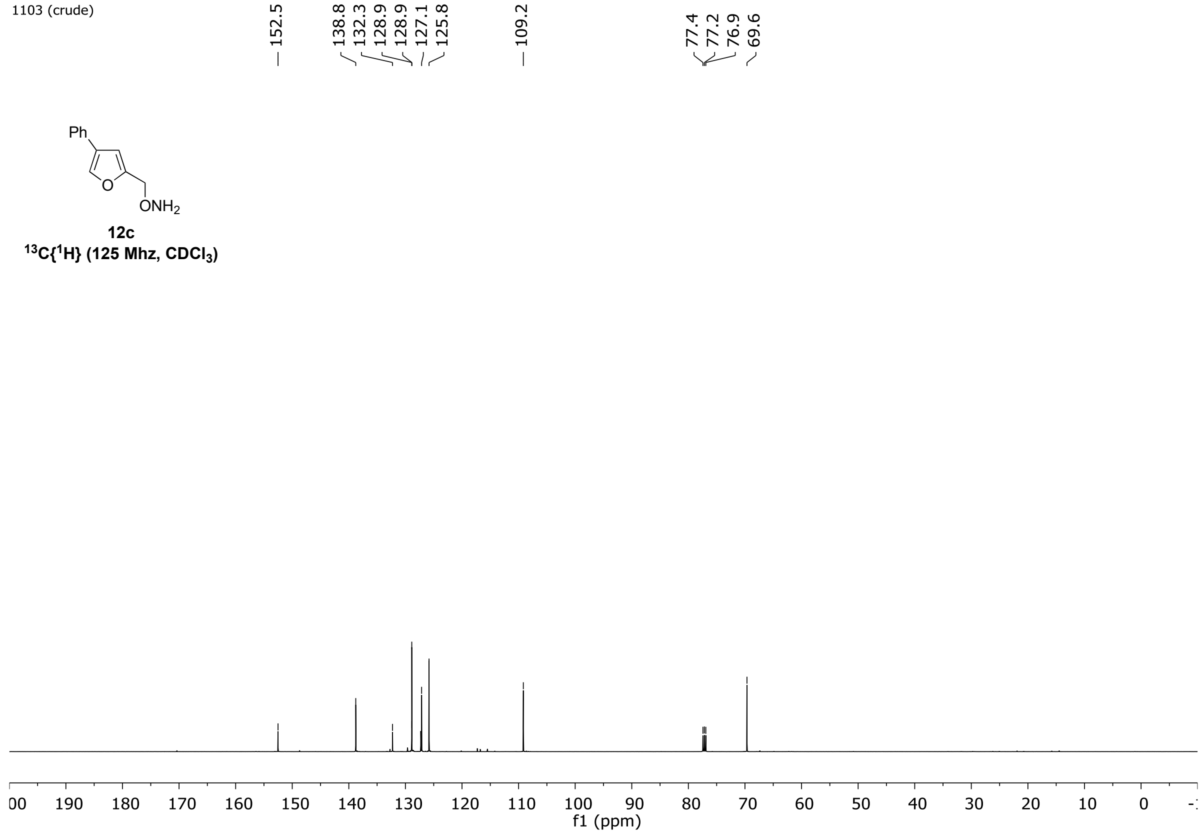

$^{13}C\{^{1}H\}$ NMR Spectrum of *O*-((4-phenylfuran-2-yl)methyl)hydroxylamine (**12c**)

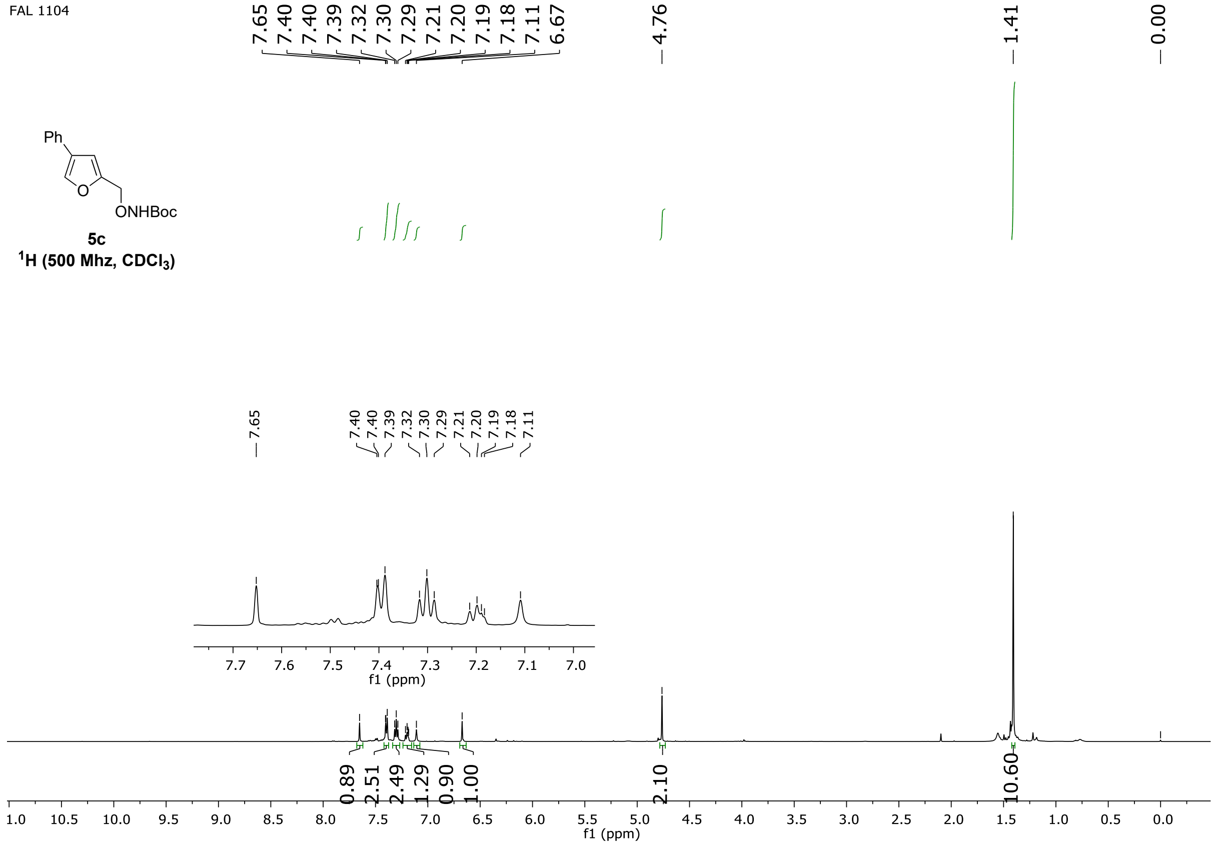

^{1}H NMR Spectrum of *tert*-butyl ((4-phenylfuran-2-yl)methoxy)carbamate (**5c**)

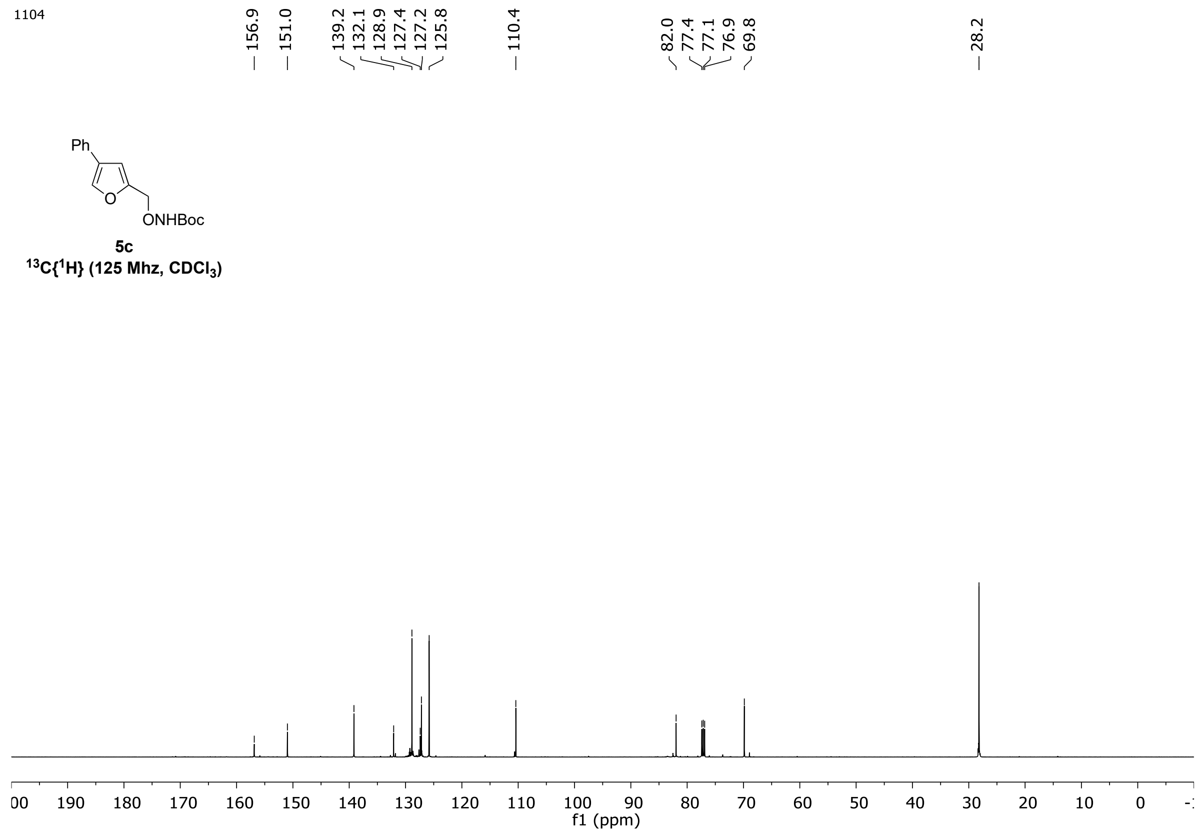

$^{13}C\{^1H\}$ NMR Spectrum of *tert*-butyl ((4-phenylfuran-2-yl)methoxy)carbamate (**5c**)

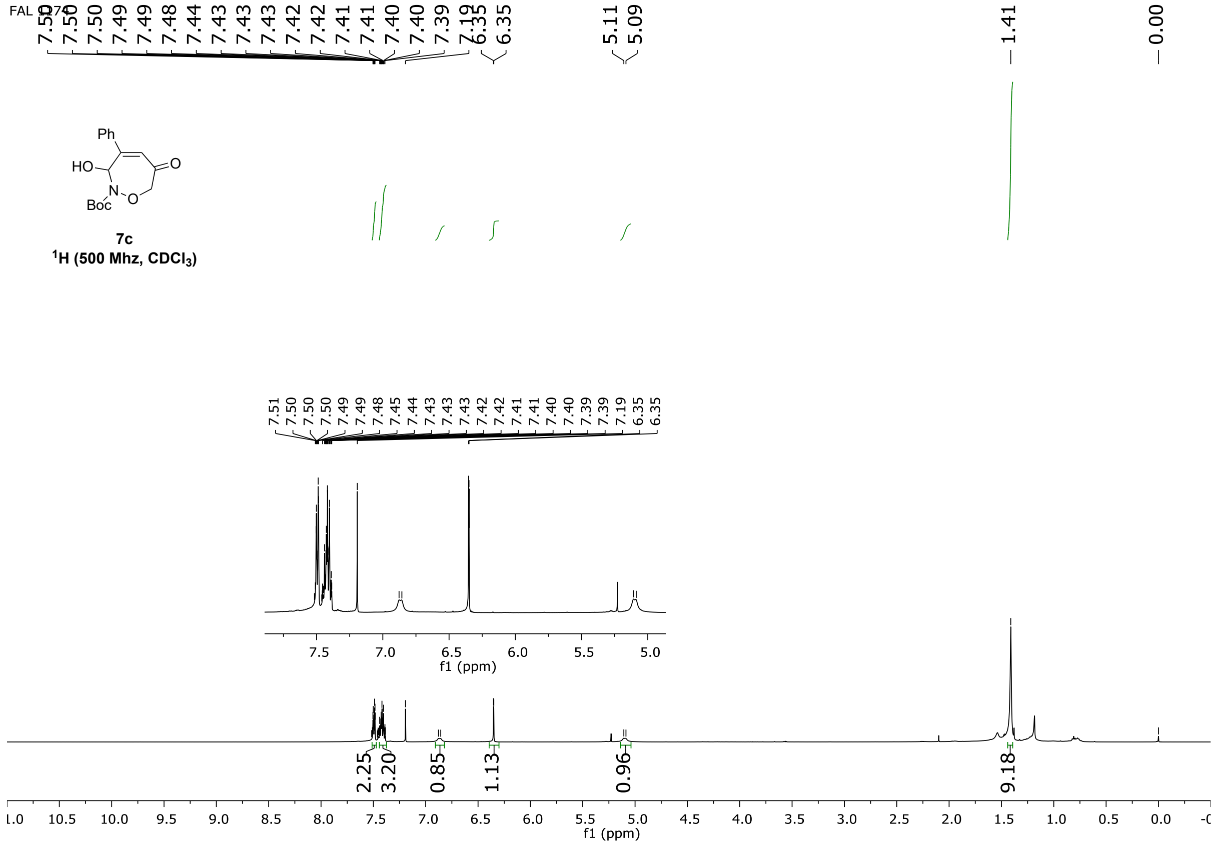

^1H NMR Spectrum of *tert*-butyl 3-hydroxy-6-oxo-4-phenyl-6,7-dihydro-1,2-oxazepine-2(*3H*)-carboxylate (**7c**)

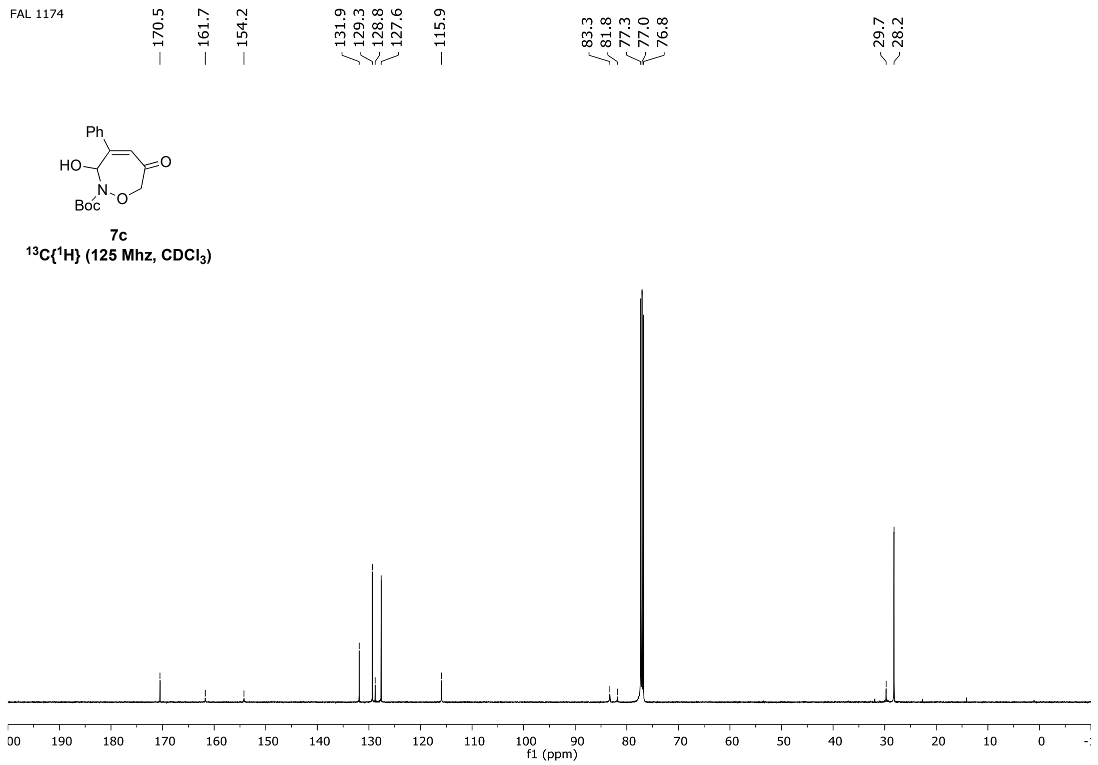

$^{13}C\{^1H\}$ NMR Spectrum of *tert*-butyl 3-hydroxy-6-oxo-4-phenyl-6,7-dihydro-1,2-oxazepine-2(*3H*)-carboxylate (**7c**)

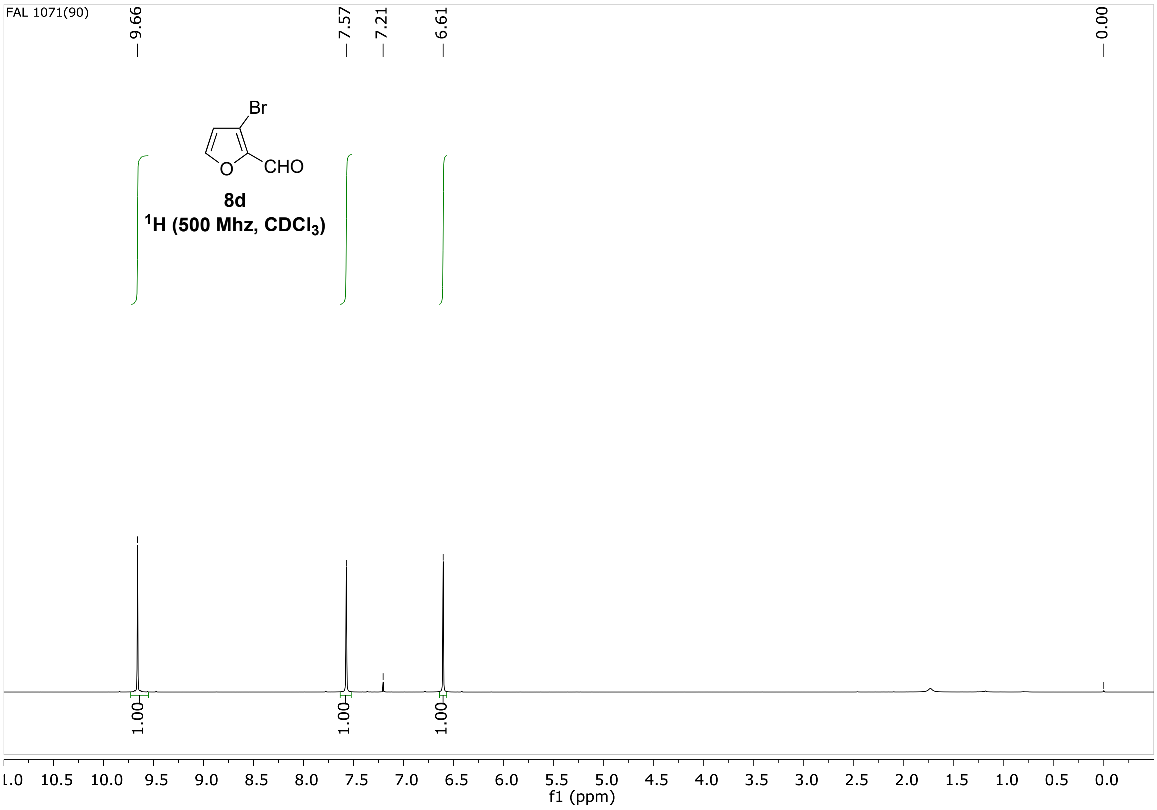

^1H NMR Spectrum of 3-bromofuran-2-carbaldehyde (**8d**)

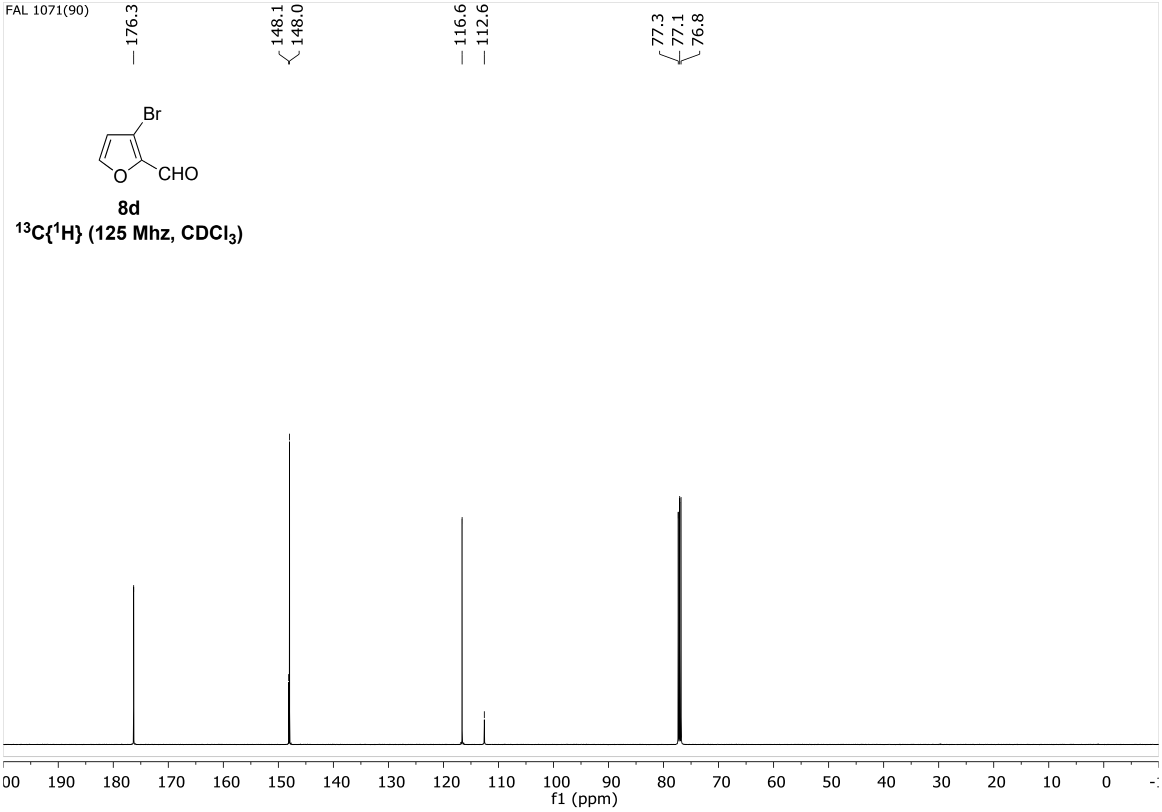

$^{13}C\{^1H\}$ NMR Spectrum of 3-bromofuran-2-carbaldehyde (**8d**)

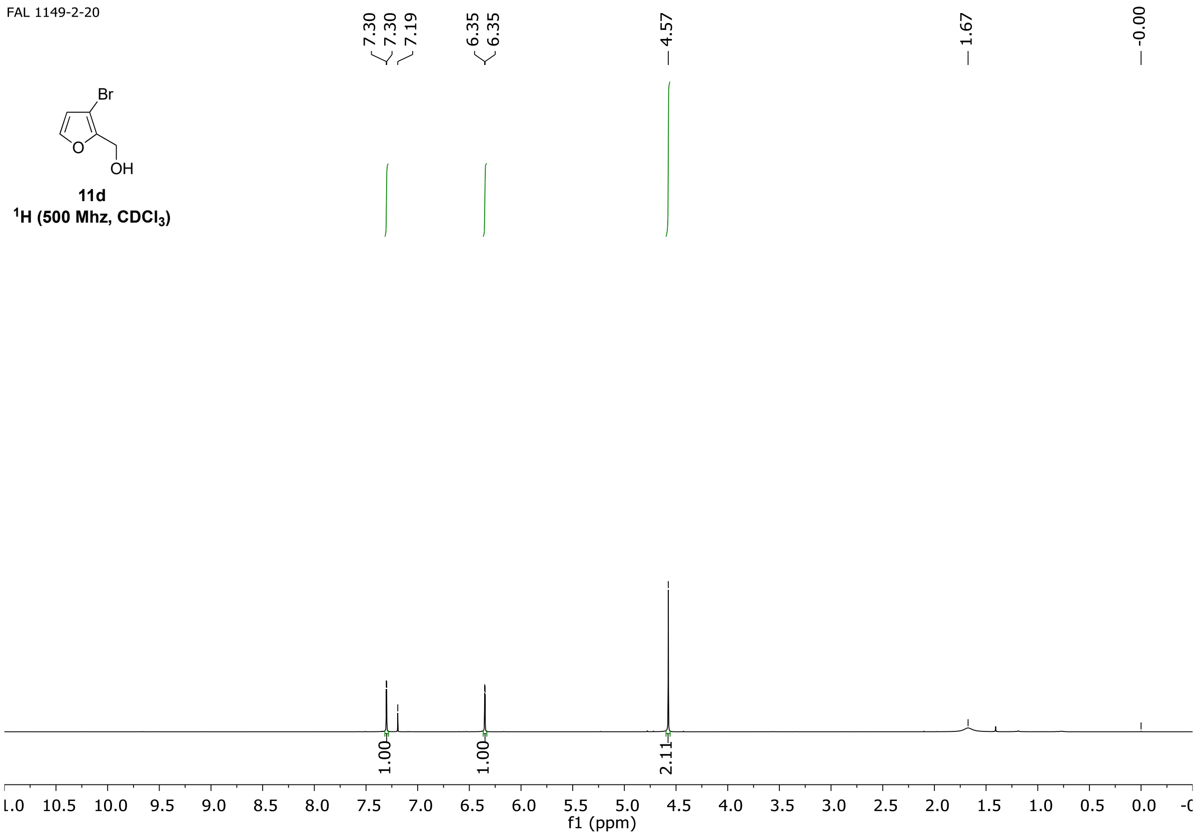

^1H NMR Spectrum of (3-bromofuran-2-yl)methanol (**11d**)

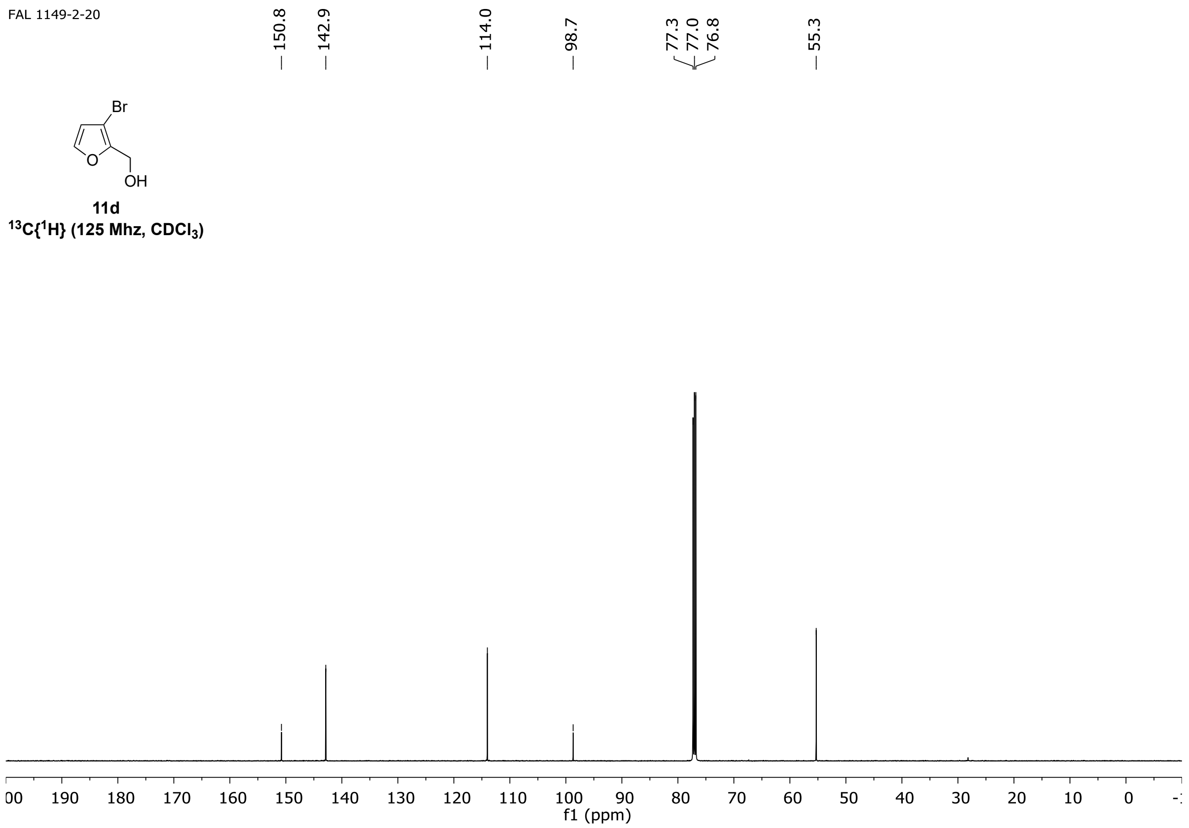

$^{13}C\{^{1}H\}$ NMR Spectrum of (3-bromofuran-2-yl)methanol (**11d**)

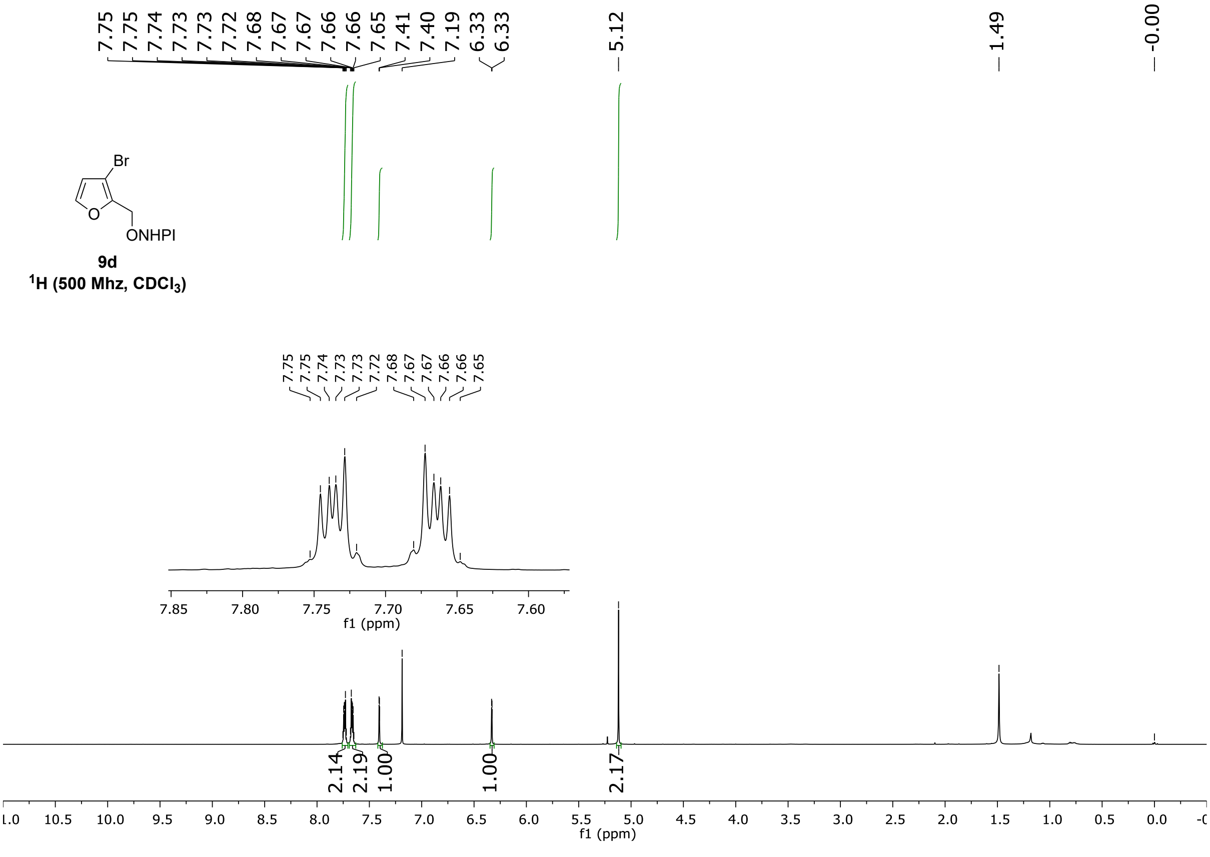

^{1}H NMR Spectrum of 2-((3-bromofuran-2-yl)methoxy)isoindoline-1,3-dione (**9d**)

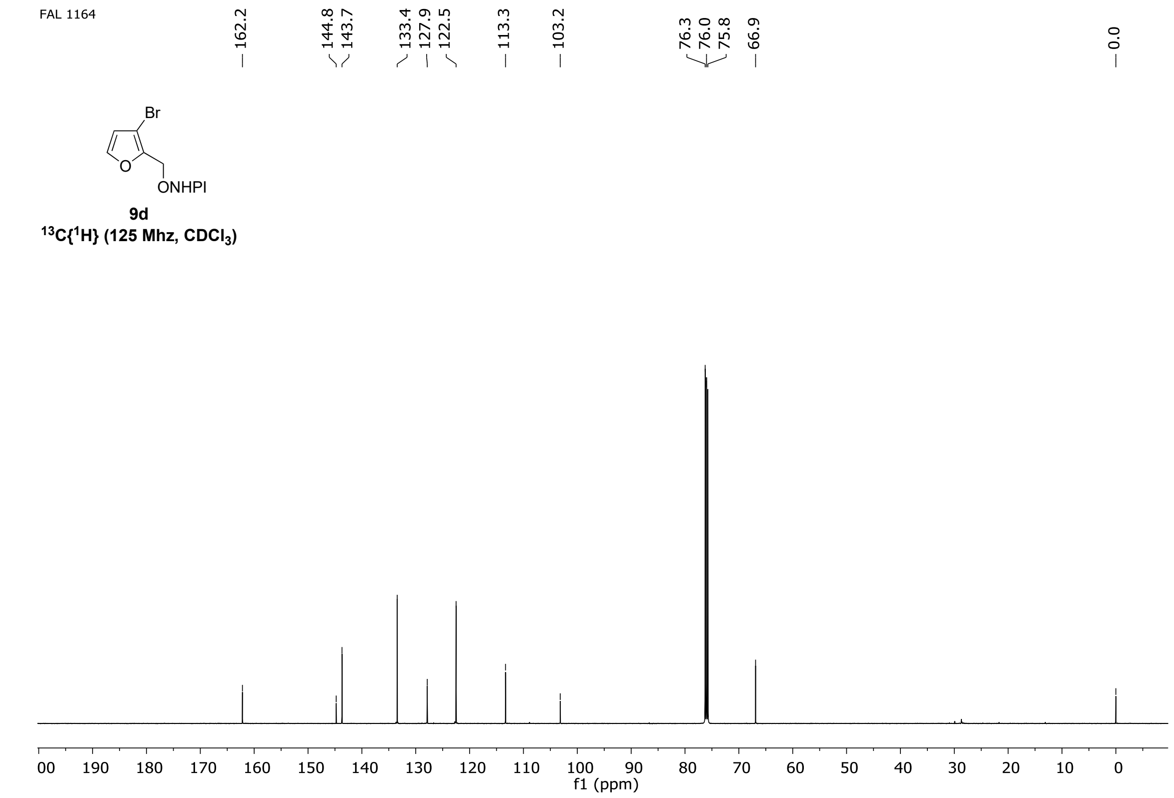

$^{13}C\{^1H\}$ NMR Spectrum of 2-((3-bromofuran-2-yl)methoxy)isoindoline-1,3-dione (**9d**)

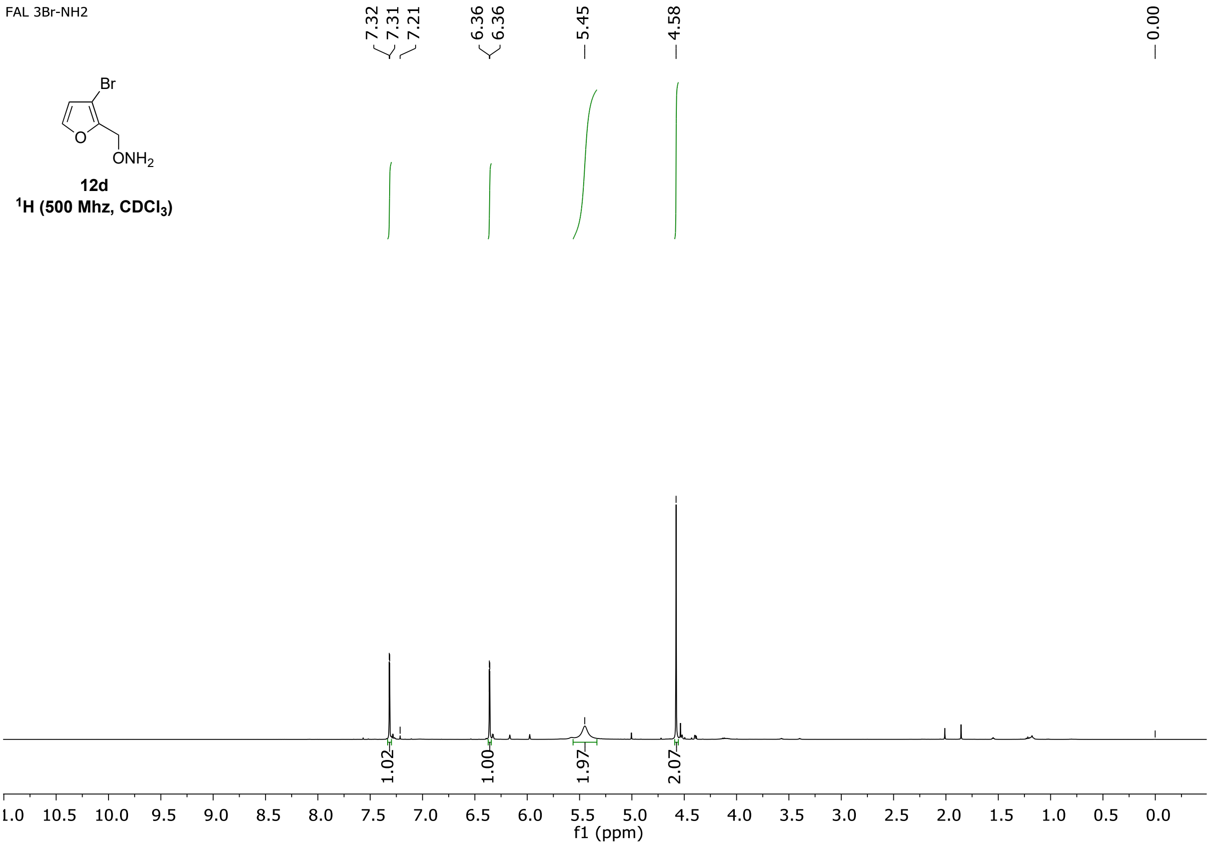

^1H NMR Spectrum of *O*-((3-bromofuran-2-yl)methyl)hydroxylamine (**12d**)

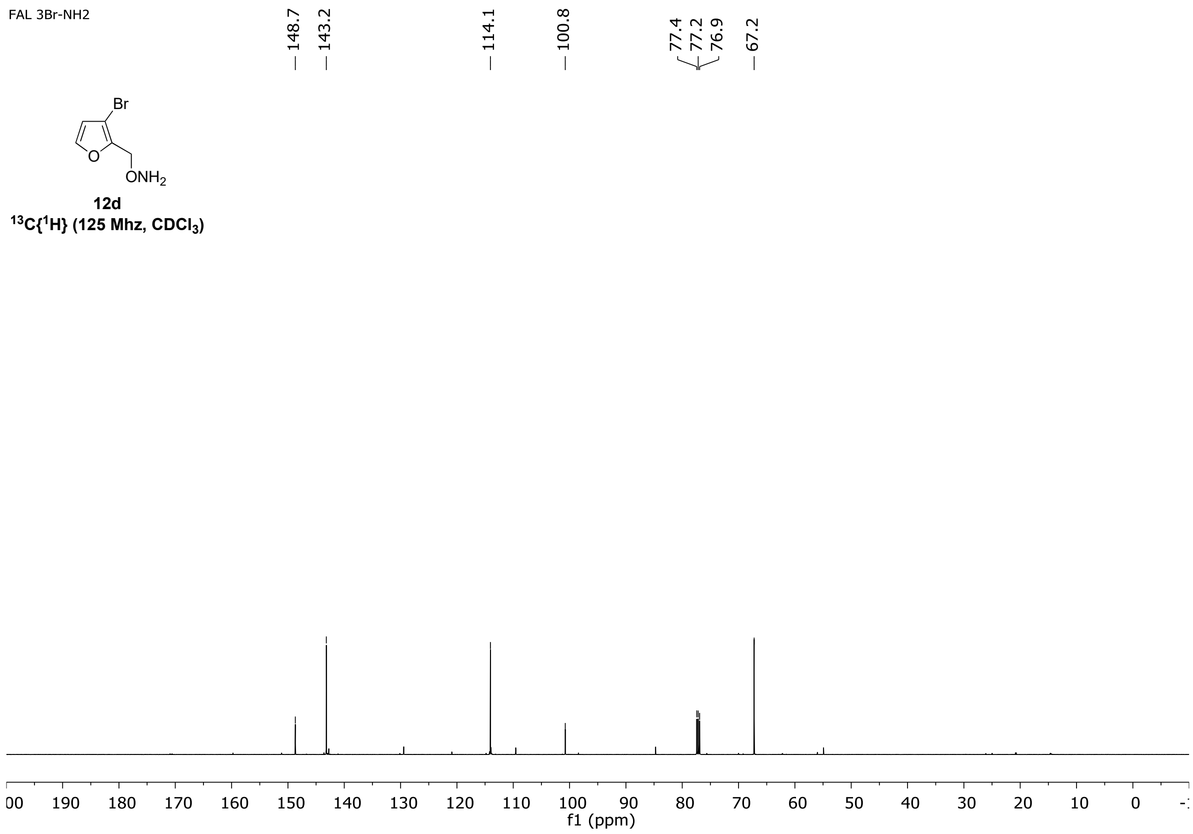

$^{13}C\{^1H\}$ NMR Spectrum of *O*-((3-bromofuran-2-yl)methyl)hydroxylamine (**12d**)

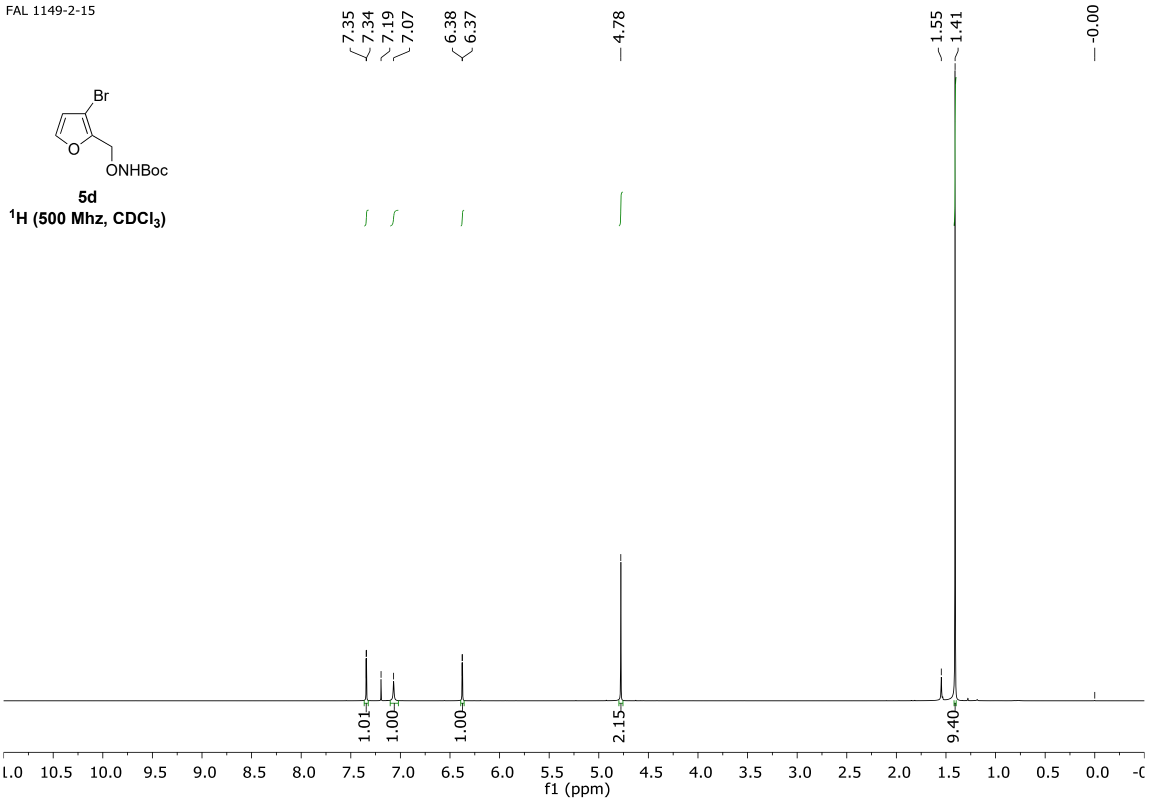

^1H NMR Spectrum of *tert*-butyl ((3-bromofuran-2-yl)methoxy)carbamate (**5d**)

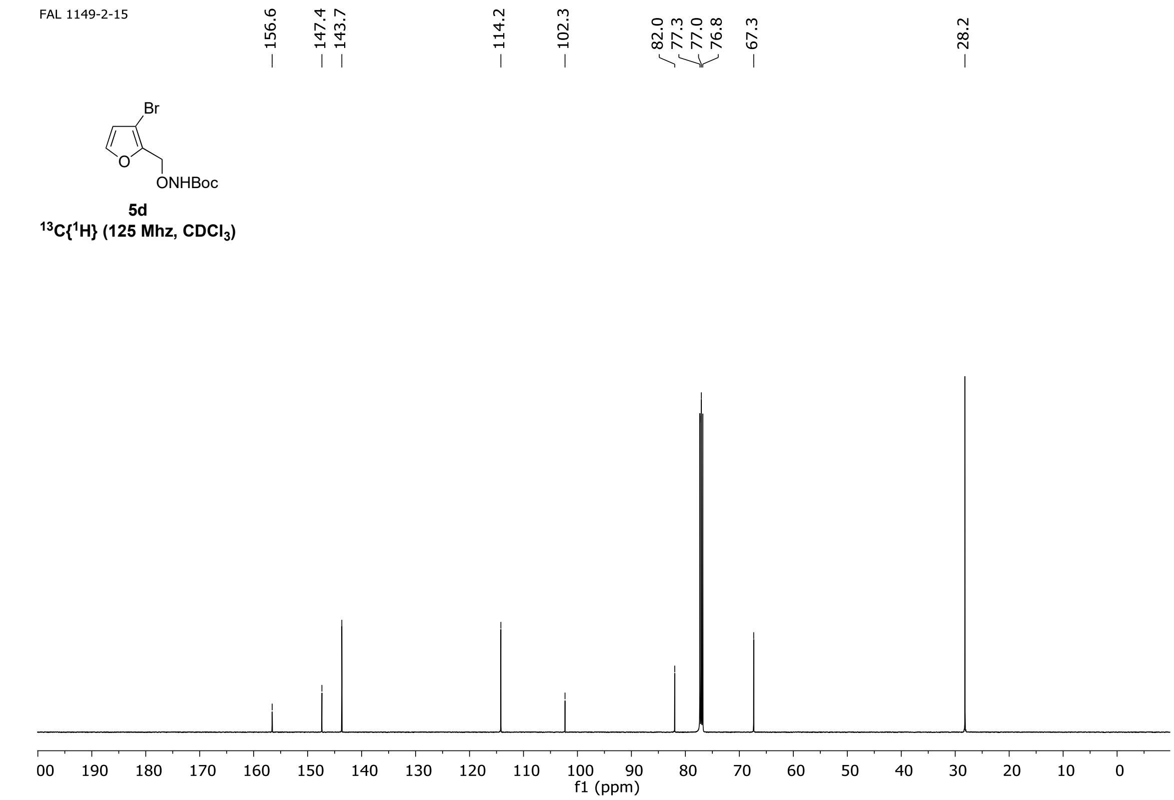

$^{13}C\{^1H\}$ NMR Spectrum of *tert*-butyl ((3-bromofuran-2-yl)methoxy)carbamate (**5d**)

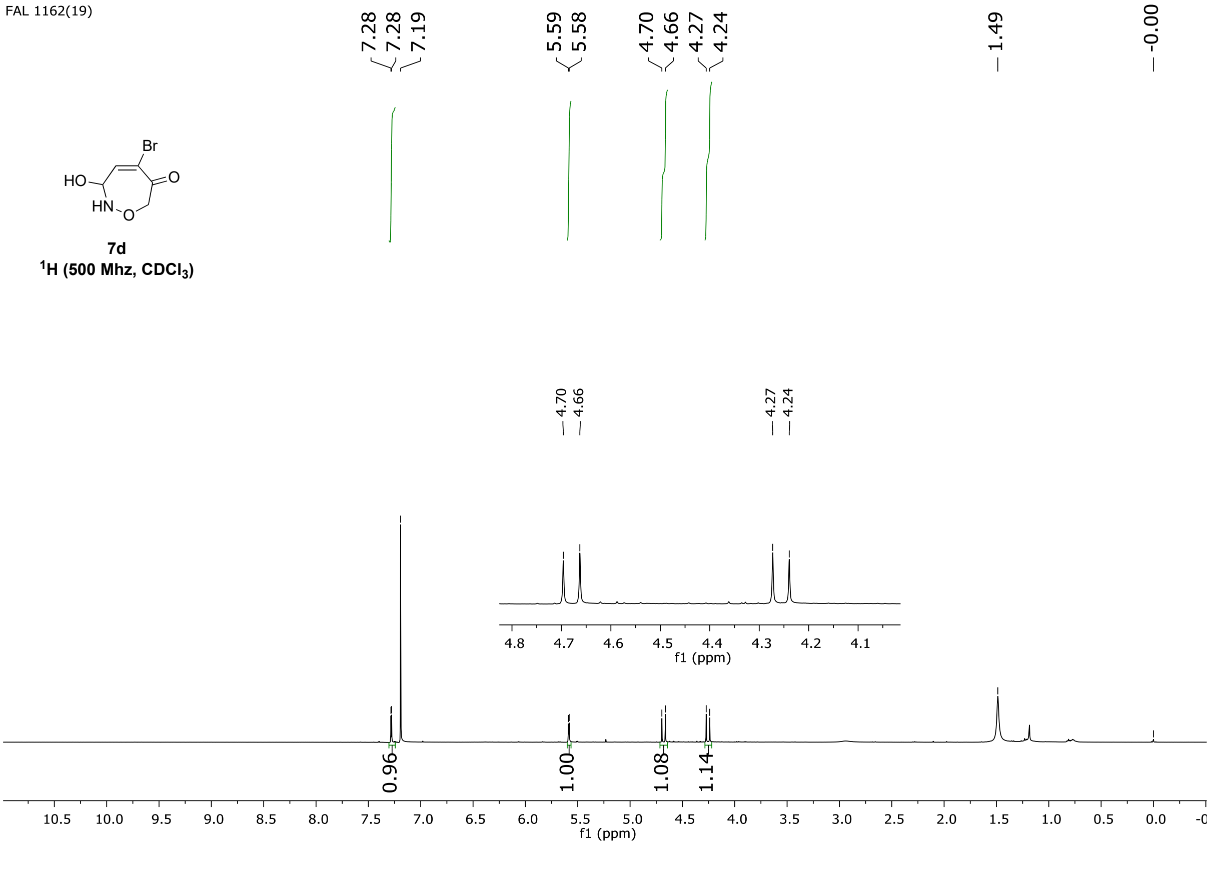

^1H NMR Spectrum of 5-bromo-3-hydroxy-2,3-dihydro-1,2-oxazepin-6(*7H*)-one (**7d**)

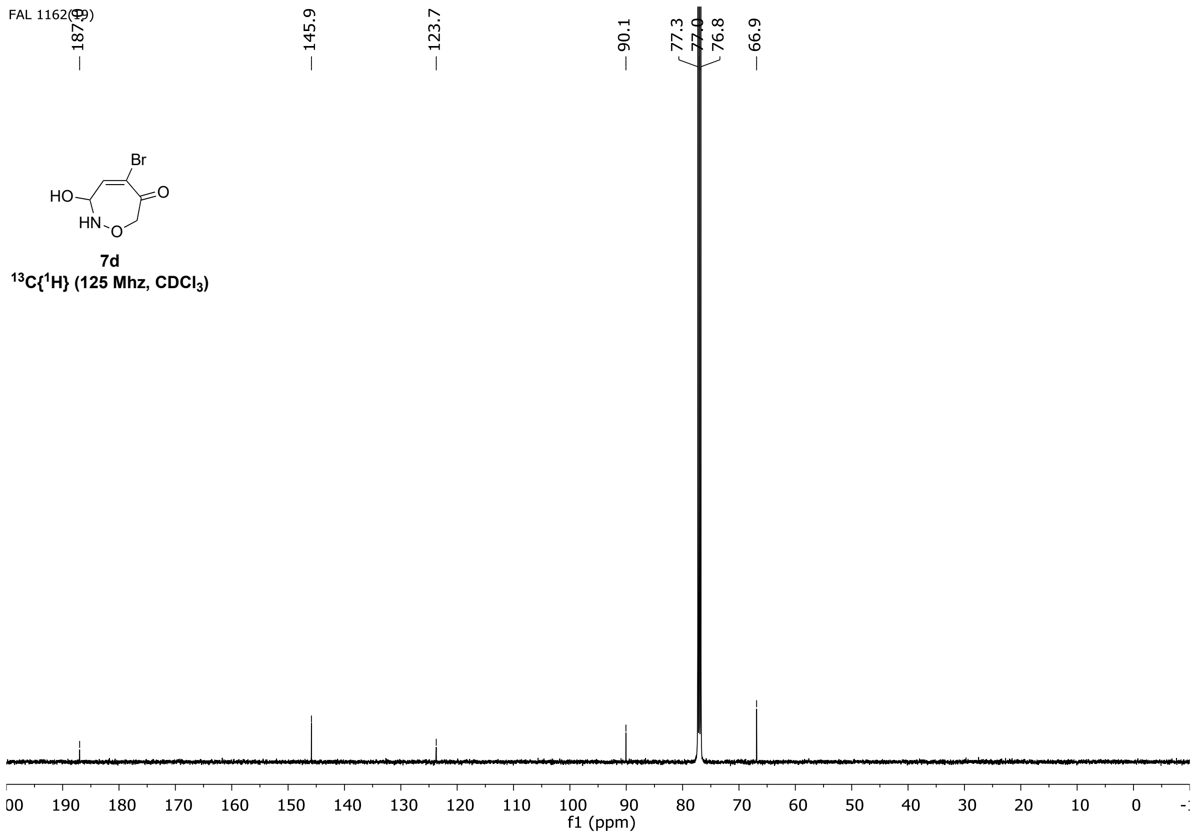

$^{13}C\{^{1}H\}$ NMR Spectrum of 5-bromo-3-hydroxy-2,3-dihydro-1,2-oxazepin-6(*7H*)-one (**7d**)

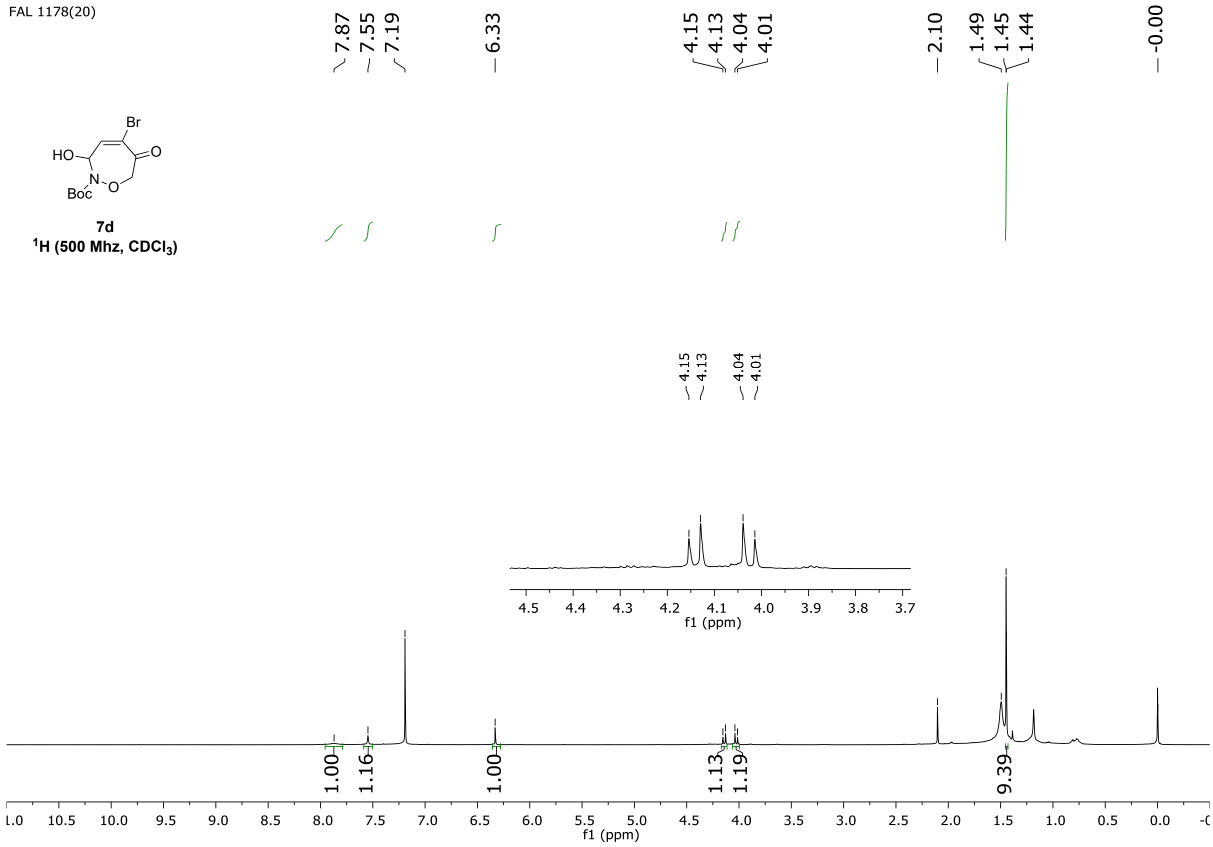

^{1}H NMR Spectrum of *tert*-butyl 5-bromo-3-hydroxy-6-oxo-6,7-dihydro-1,2-oxazepine-2(*3H*)-carboxylate (**7d**)

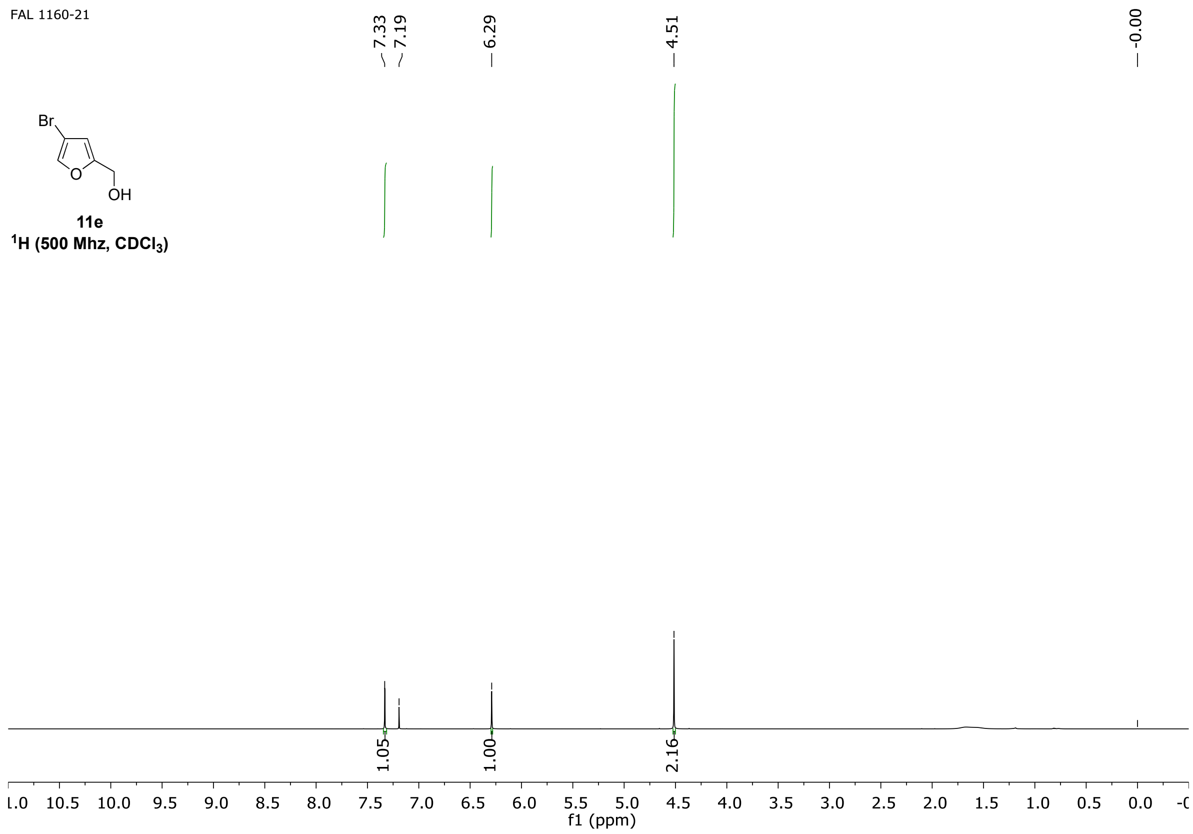

^{1}H NMR Spectrum of (4-bromofuran-2-yl)methanol (**11e**)

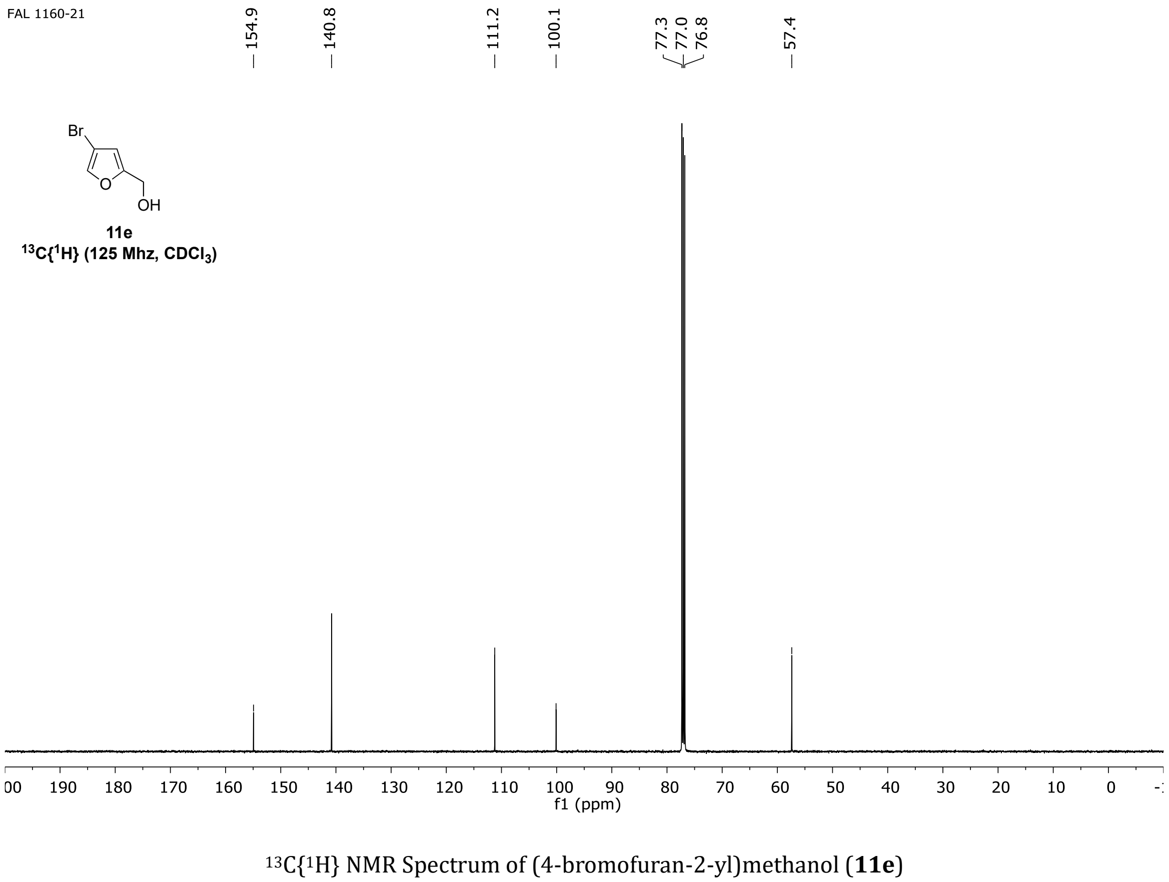

^{13}C{^{1}H} NMR Spectrum of (4-bromofuran-2-yl)methanol (**11e**)

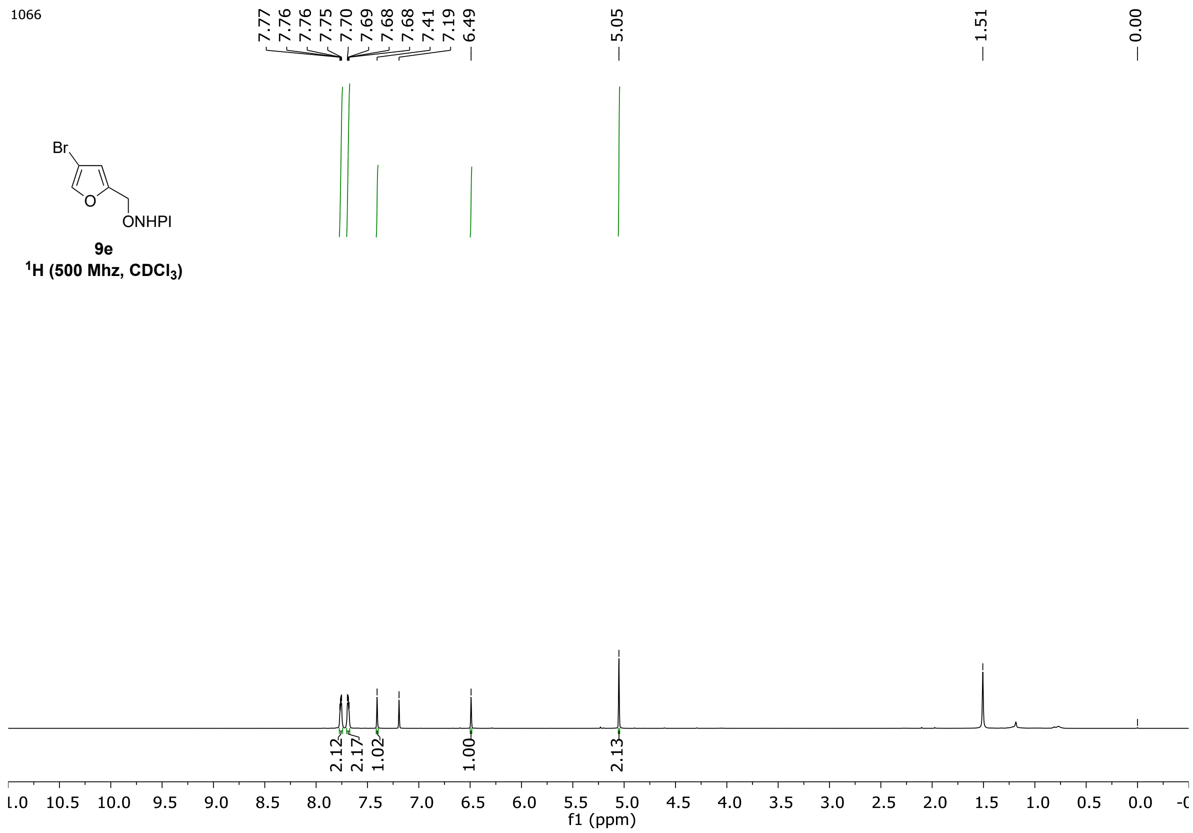

^{1}H NMR Spectrum of 2-((4-bromofuran-2-yl)methoxy)isoindoline-1,3-dione (**9e**)

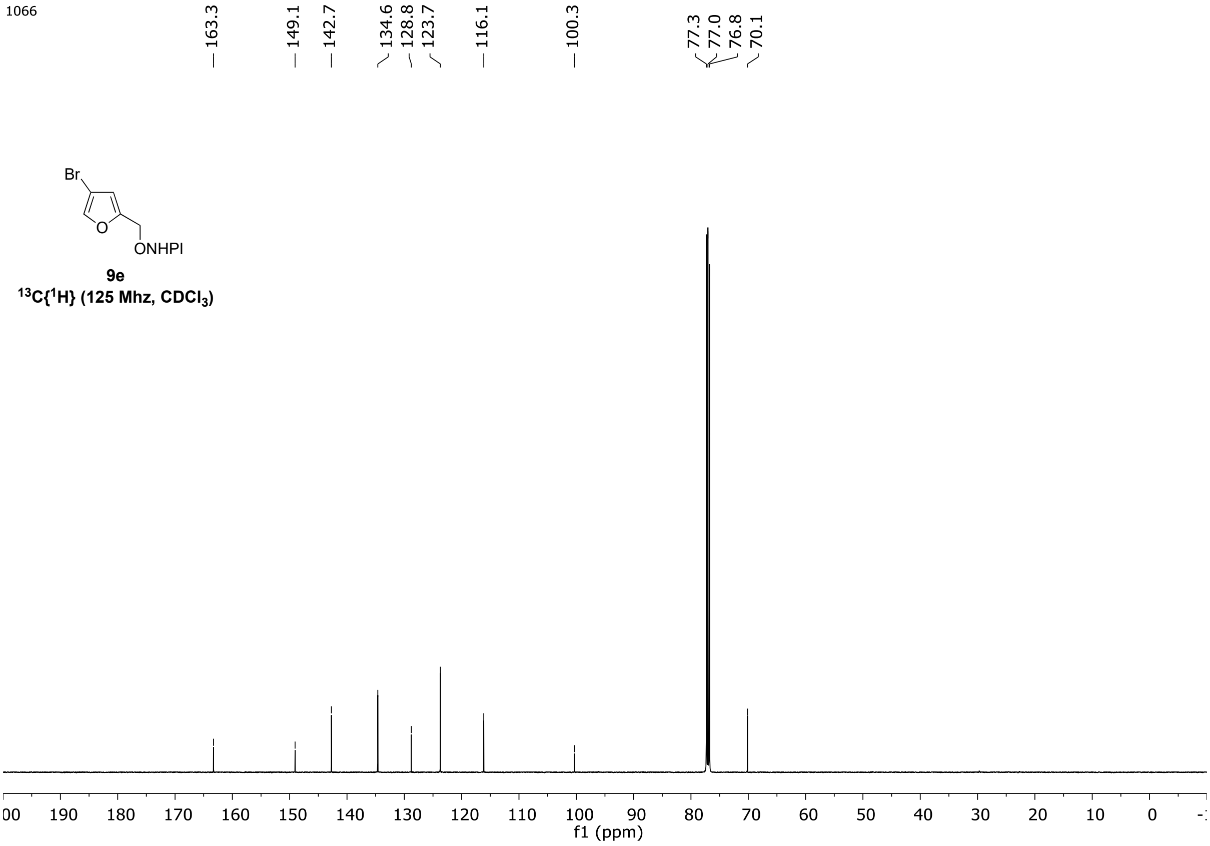

$^{13}C\{^{1}H\}$ NMR Spectrum of 2-((4-bromofuran-2-yl)methoxy)isoindoline-1,3-dione (**9e**)

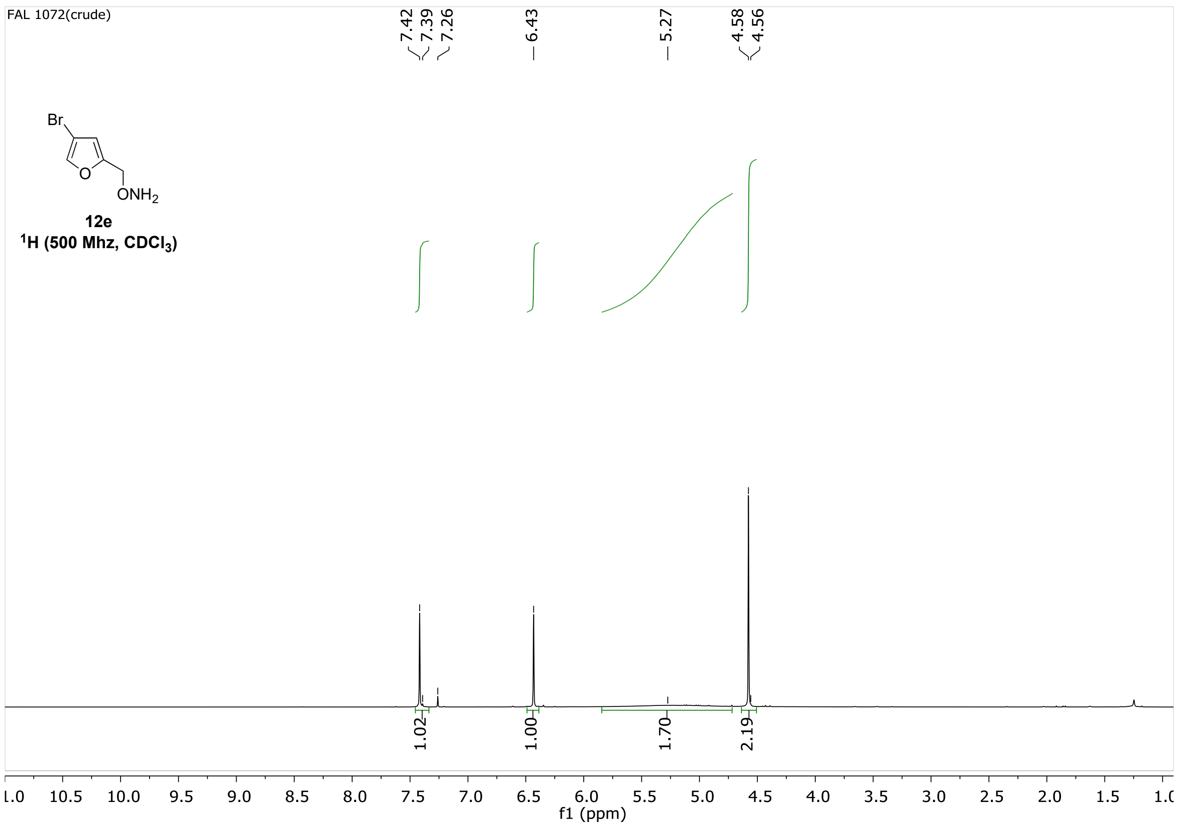

^{1}H NMR Spectrum of *O*-((4-bromofuran-2-yl)methyl)hydroxylamine (**12e**)

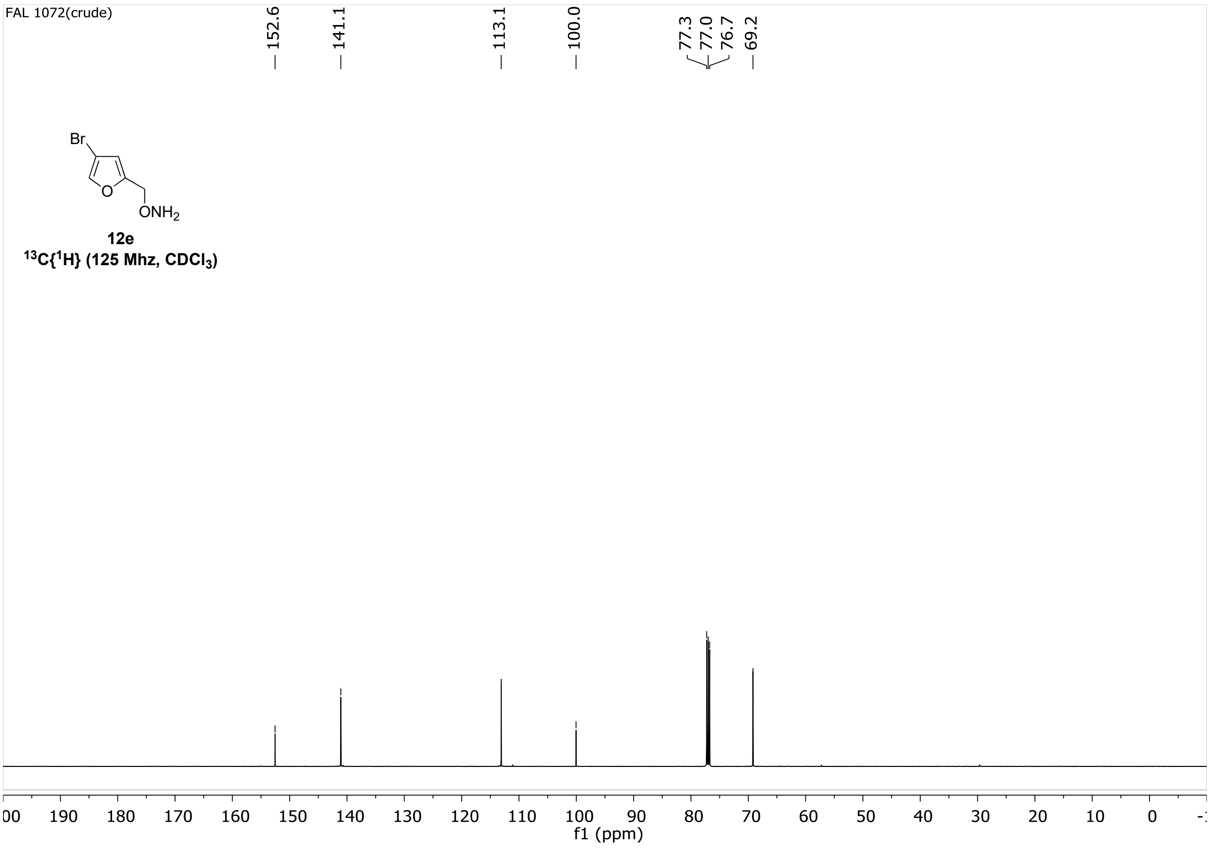

^{13}C{^{1}H} NMR Spectrum of *O*-((4-bromofuran-2-yl)methyl)hydroxylamine (**12e**)

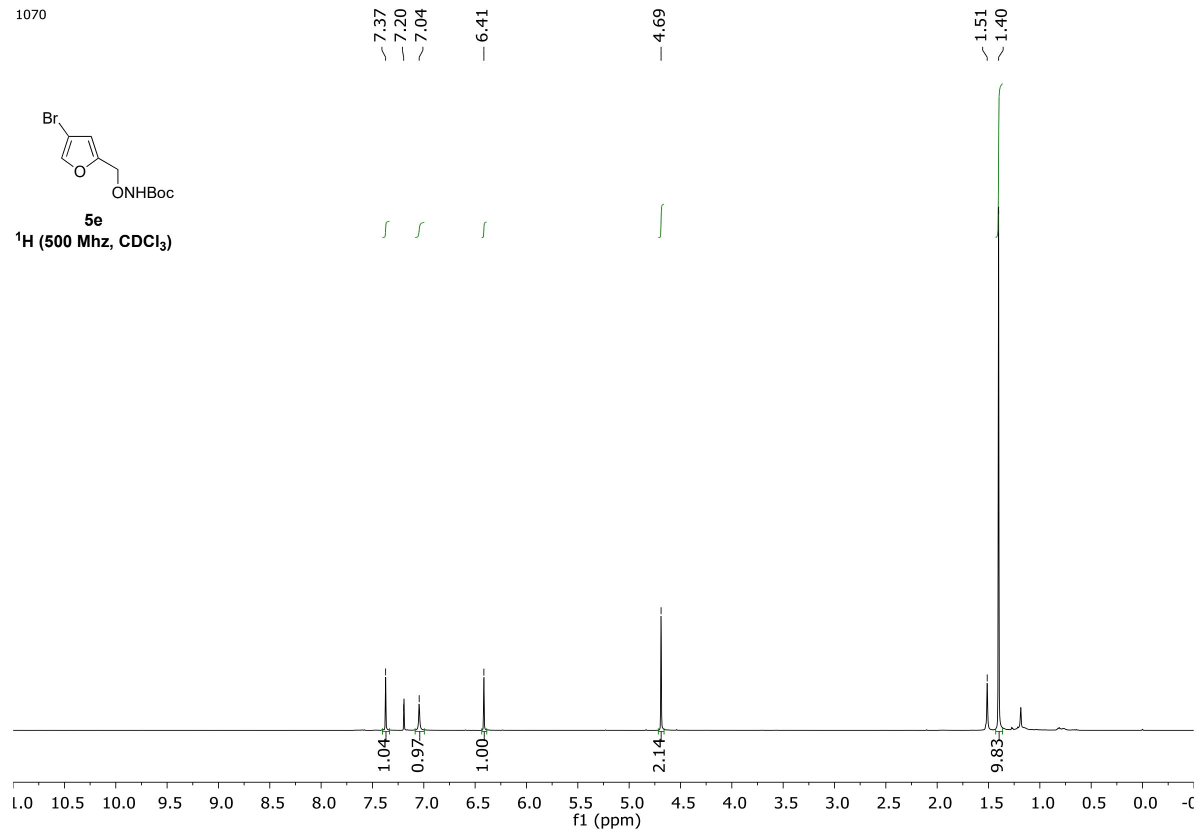

^{1}H NMR Spectrum of *tert*-butyl ((4-bromofuran-2-yl)methoxy)carbamate (**5e**)

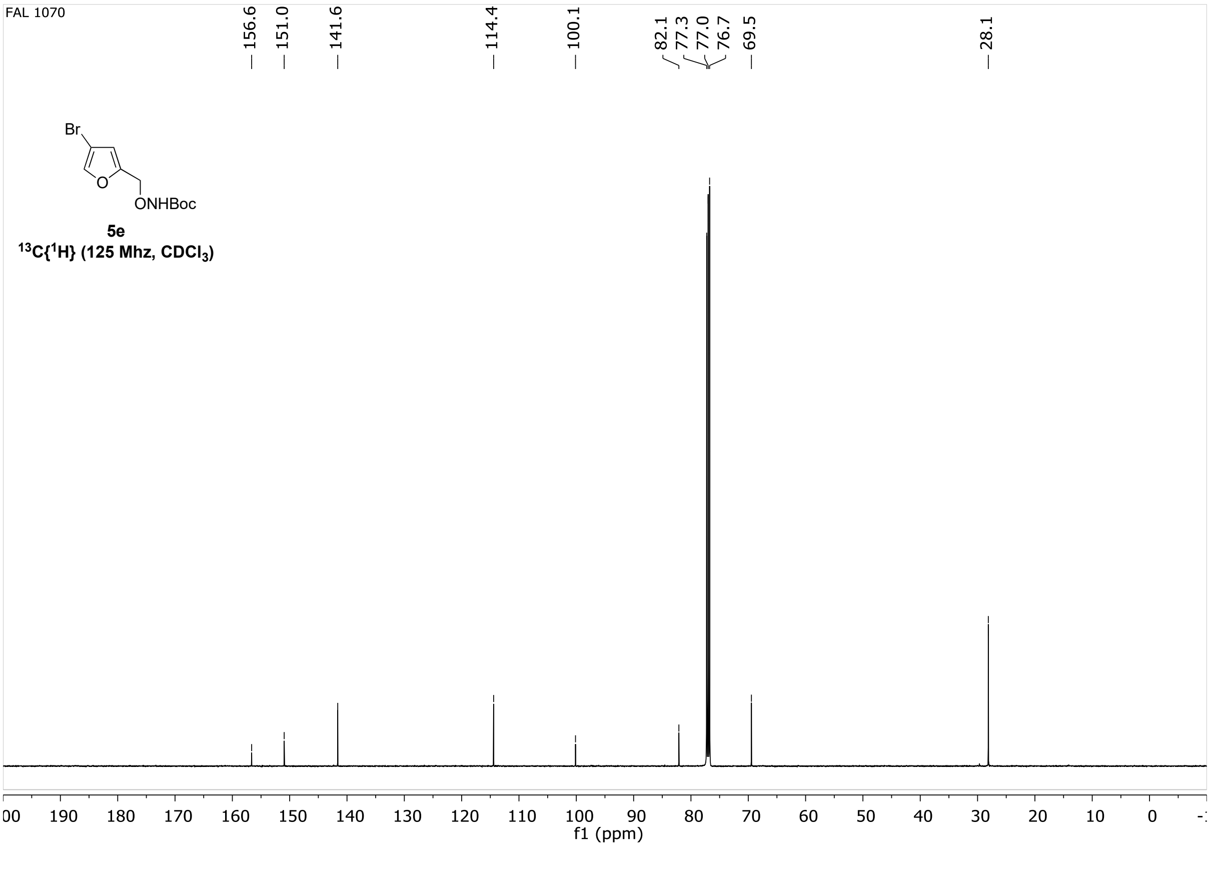

^{13}C{^{1}H} NMR Spectrum of *tert*-butyl ((4-bromofuran-2-yl)methoxy)carbamate (**5e**)

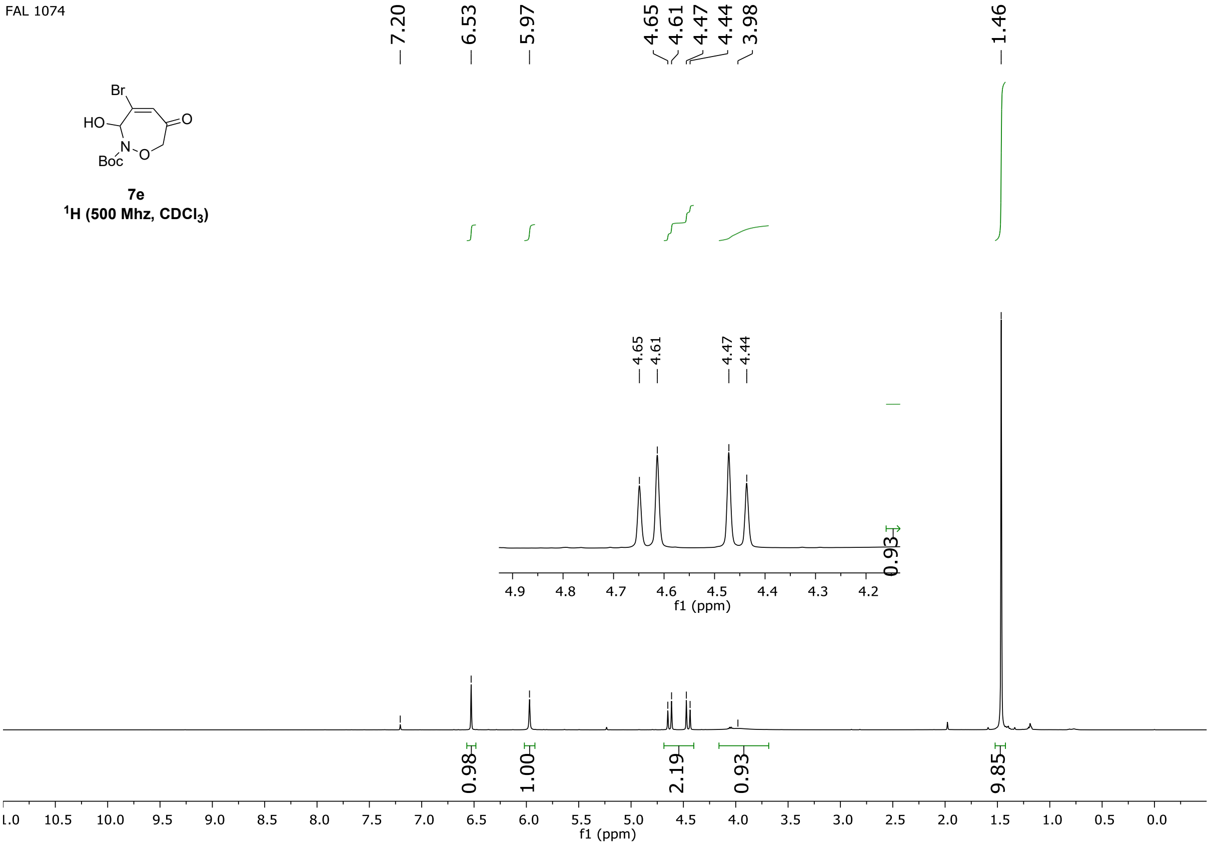

^{1}H NMR Spectrum of *tert*-butyl 4-bromo-3-hydroxy-6-oxo-6,7-dihydro-1,2-oxazepine-2(*3H*)-carboxylate (**7e**)

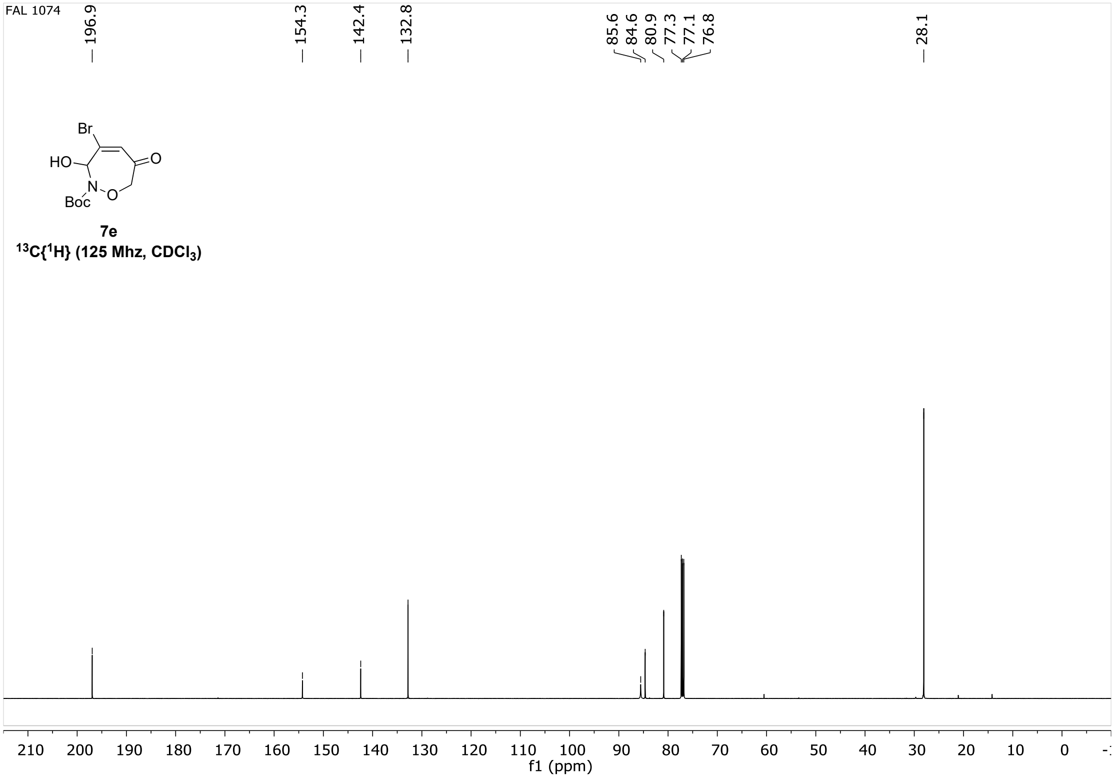

^{13}C{^{1}H} NMR Spectrum of *tert*-butyl 4-bromo-3-hydroxy-6-oxo-6,7-dihydro-1,2-oxazepine-2(*3H*)-carboxylate (**7e**)

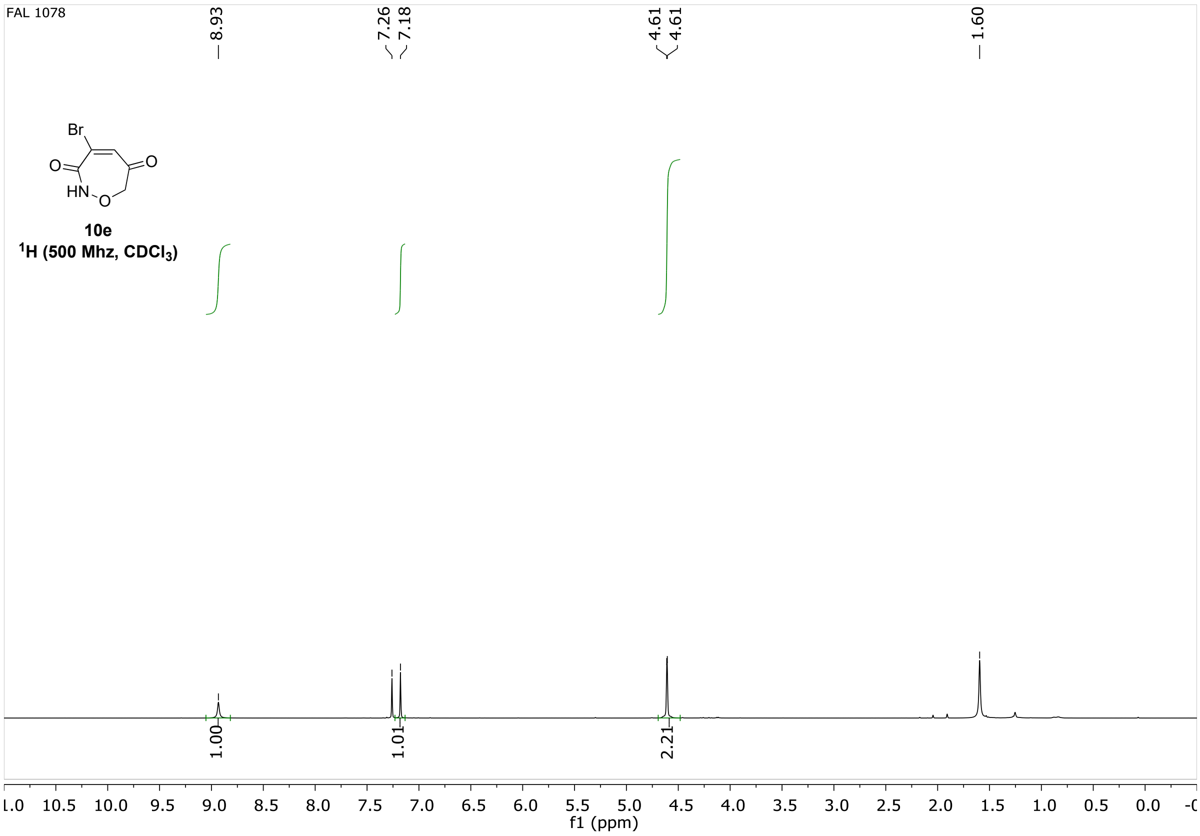

^{1}H NMR Spectrum of 4-bromo-1,2-oxazepine-3,6(*2H,7H*)-dione (**10e**)

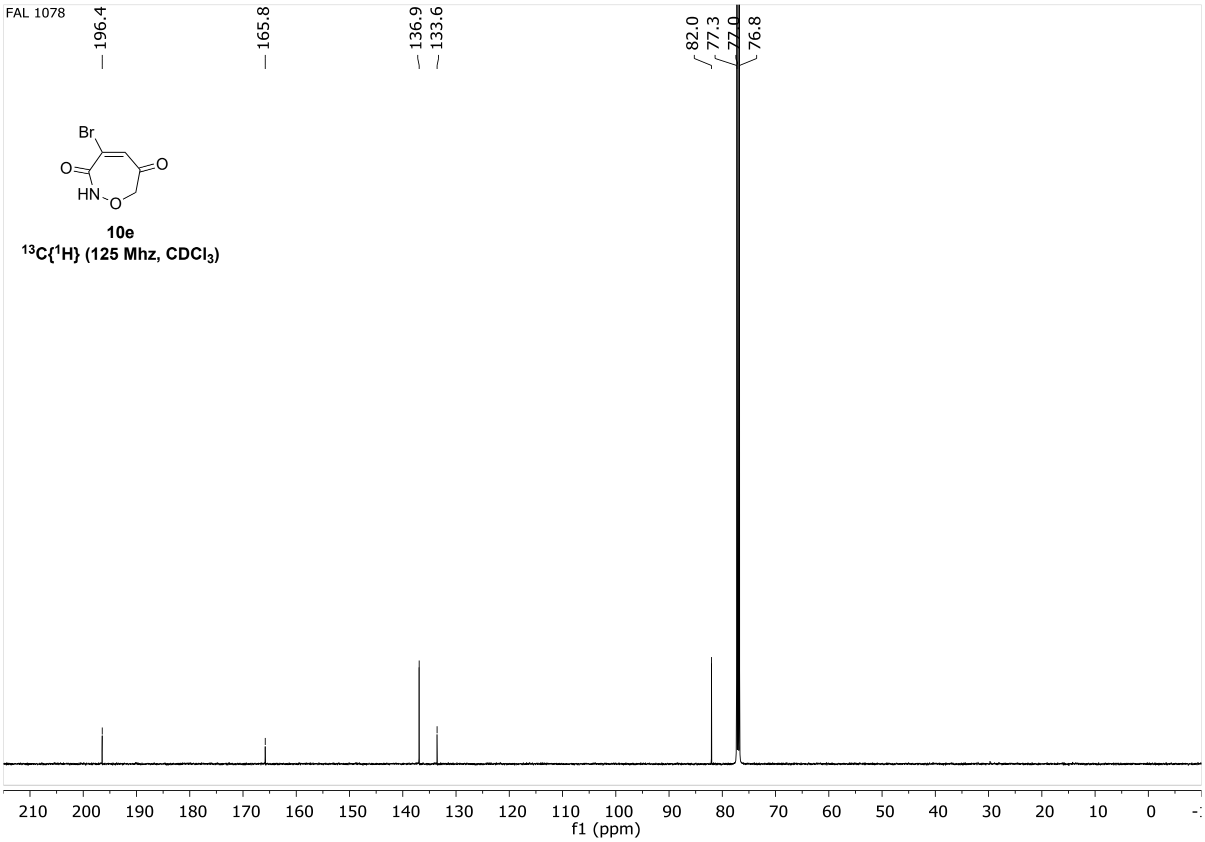

^{13}C{^{1}H} NMR Spectrum of 4-bromo-1,2-oxazepine-3,6(*2H,7H*)-dione (**10e**)

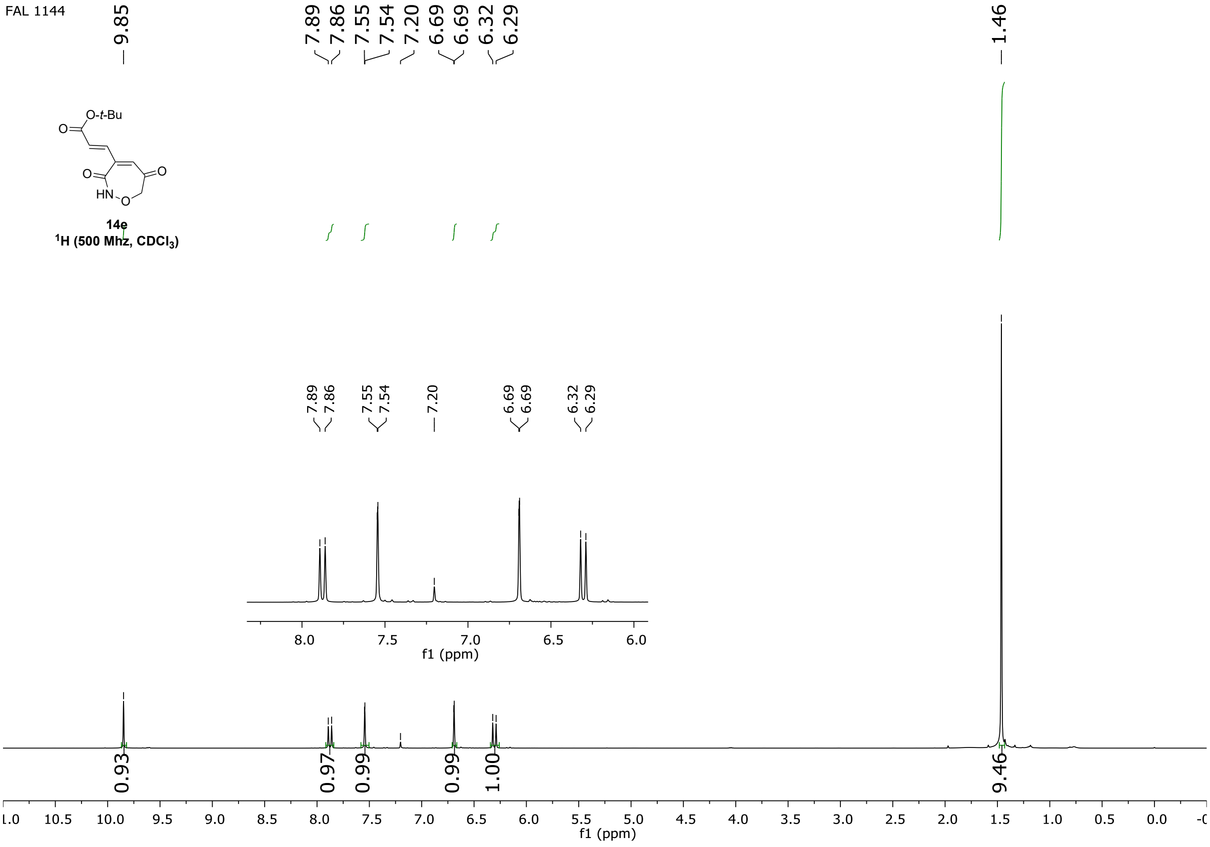

^{1}H NMR Spectrum of *tert*-butyl (*E*)-3-(6-hydroxy-3-oxo-2,3-dihydro-1,2-oxazepin-4-yl)acrylate (**14e**)

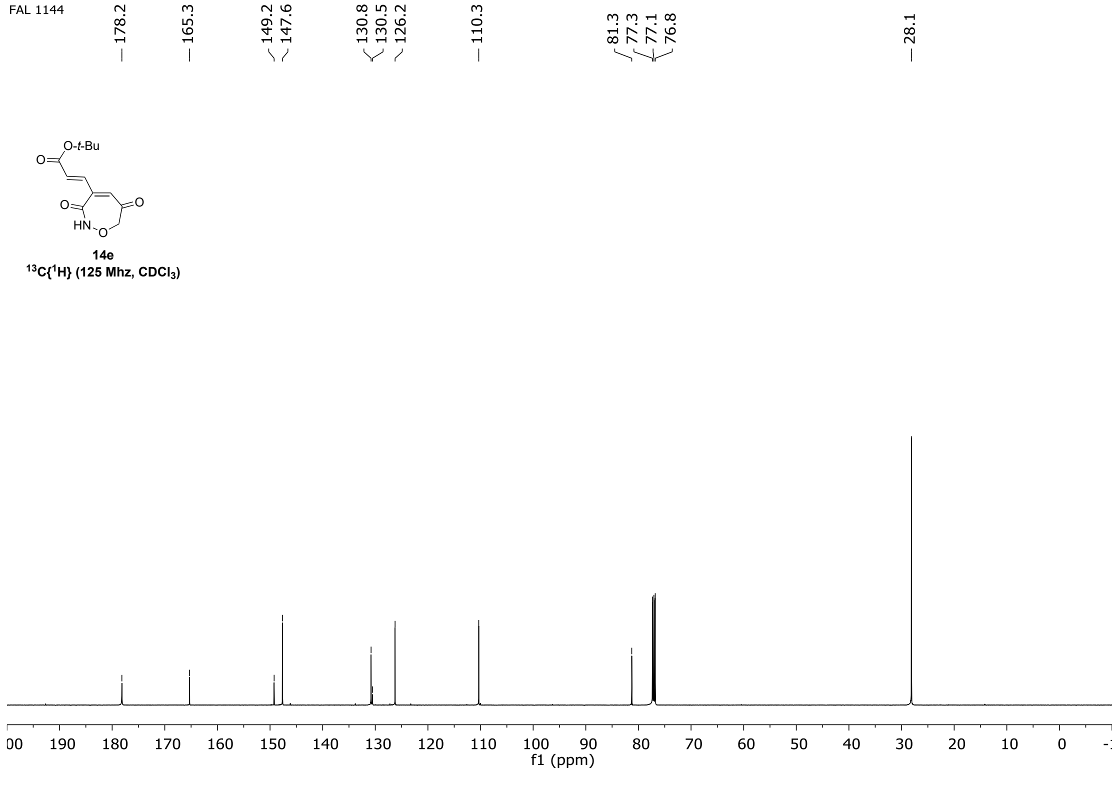

^{13}C NMR Spectrum of *tert*-butyl (*E*)-3-(6-hydroxy-3-oxo-2,3-dihydro-1,2-oxazepin-4-yl)acrylate (**14e**)

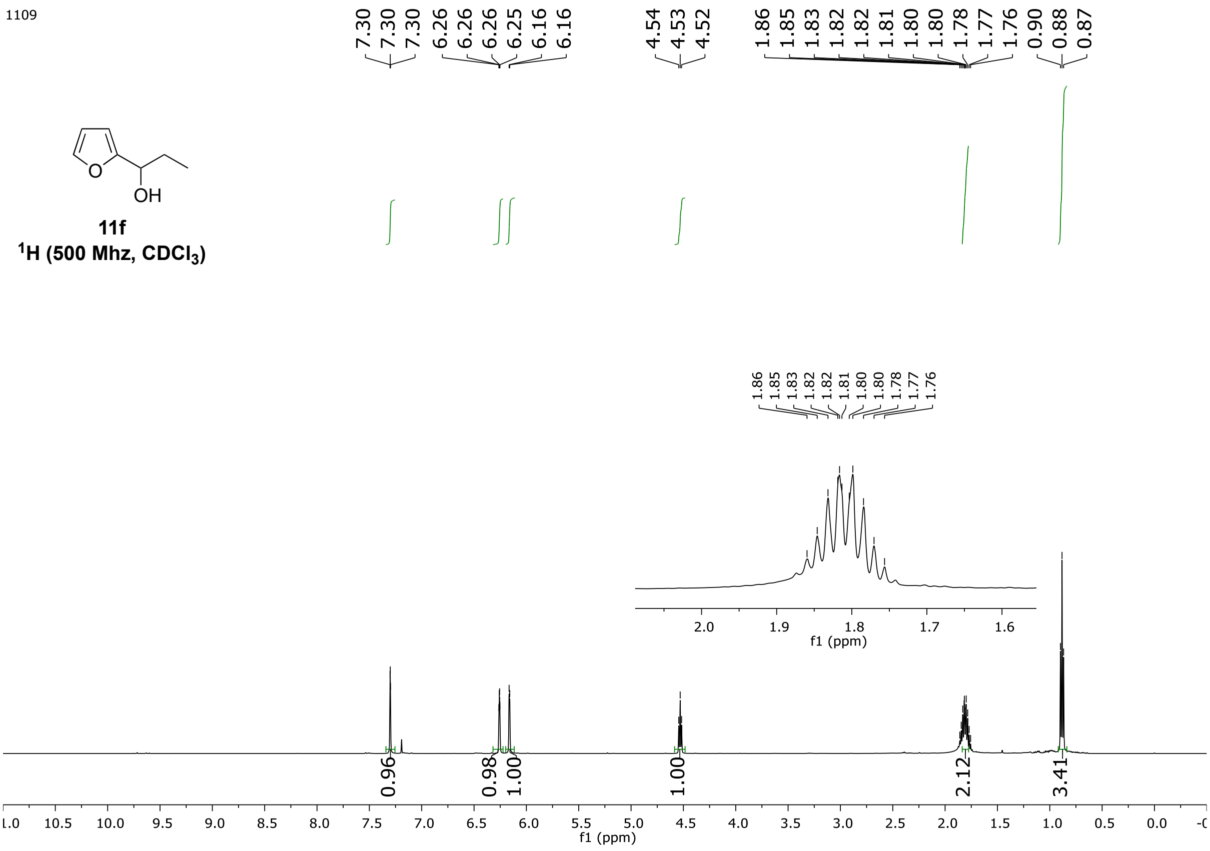

^{1}H NMR Spectrum of 1-(furan-2-yl)propan-1-ol (**11f**)

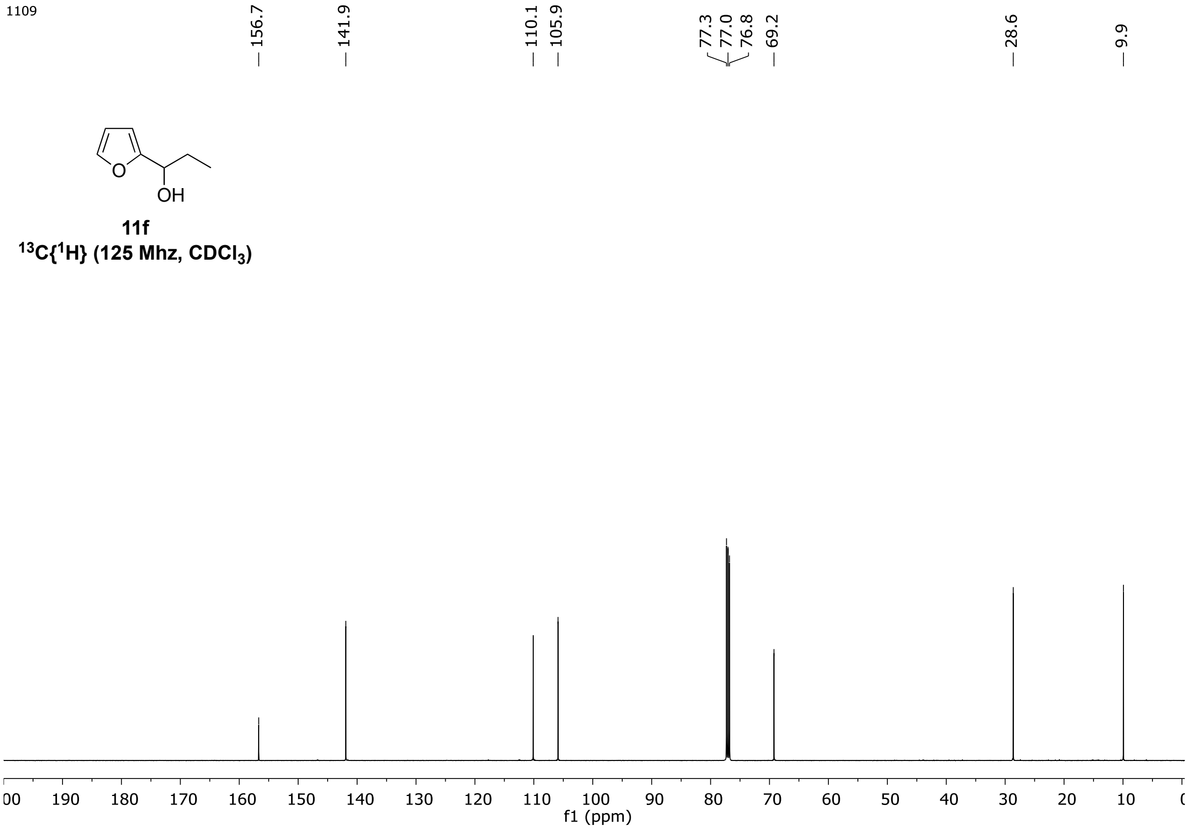

$^{13}C\{^{1}H\}$ NMR Spectrum of 1-(furan-2-yl)propan-1-ol (**11f**)

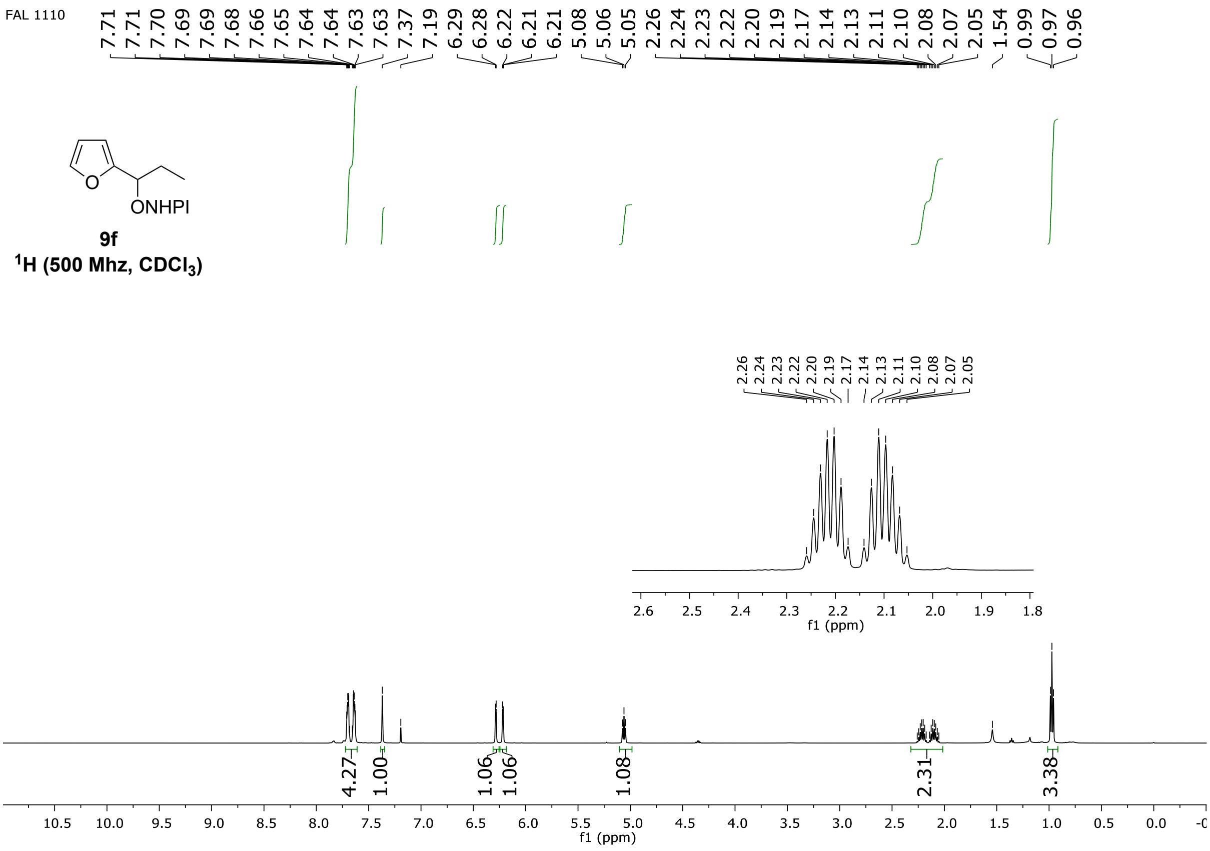

^{1}H NMR Spectrum of 2-(1-(furan-2-yl)propoxy)isoindoline-1,3-dione (**9f**)

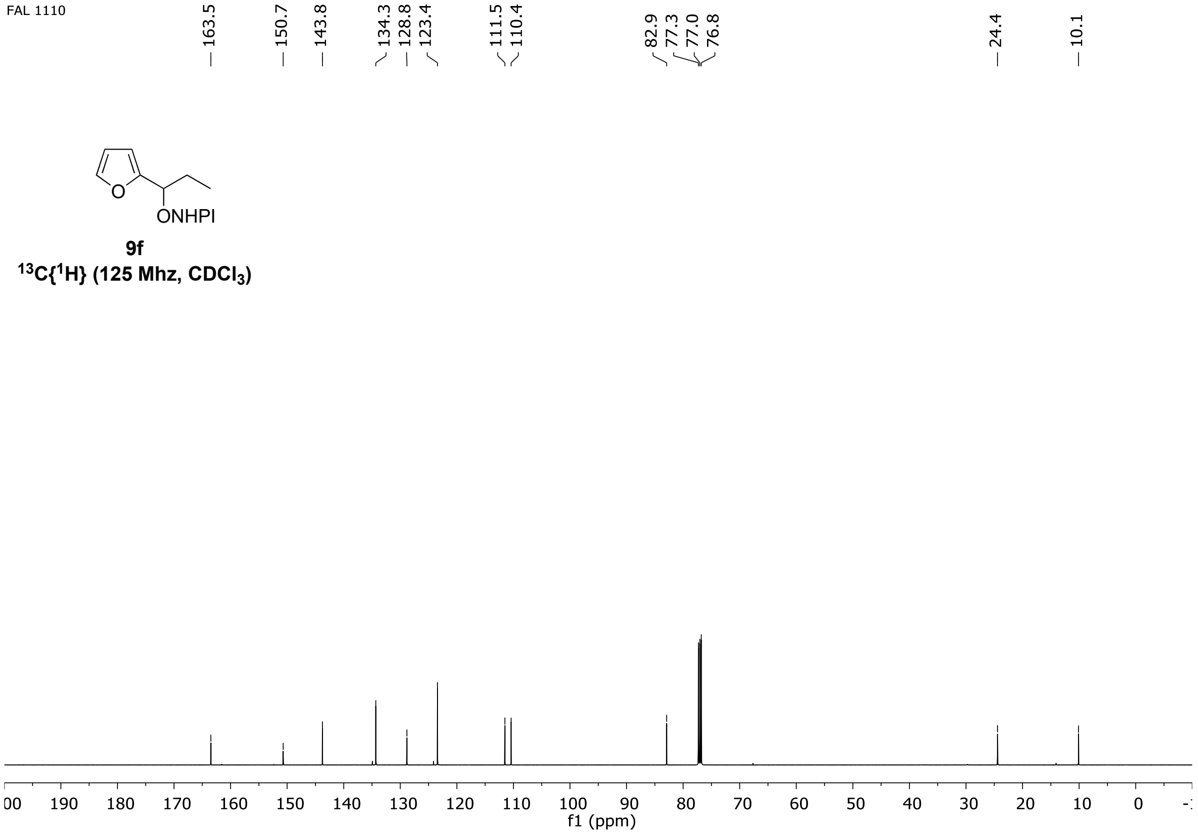

^{13}C{^{1}H} NMR Spectrum of 2-(1-(furan-2-yl)propoxy)isoindoline-1,3-dione (**9f**)

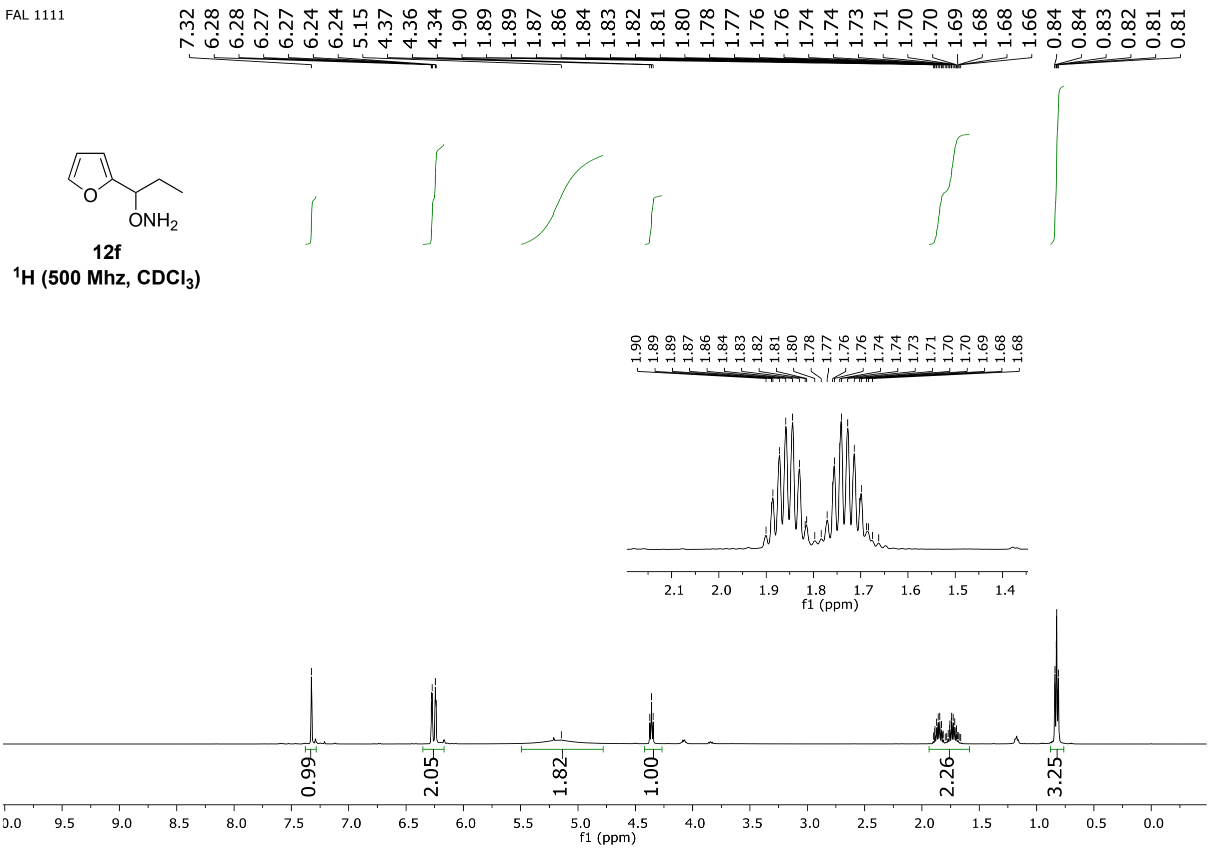

^{1}H NMR Spectrum of *O*-(1-(furan-2-yl)propyl)hydroxylamine (**12f**)

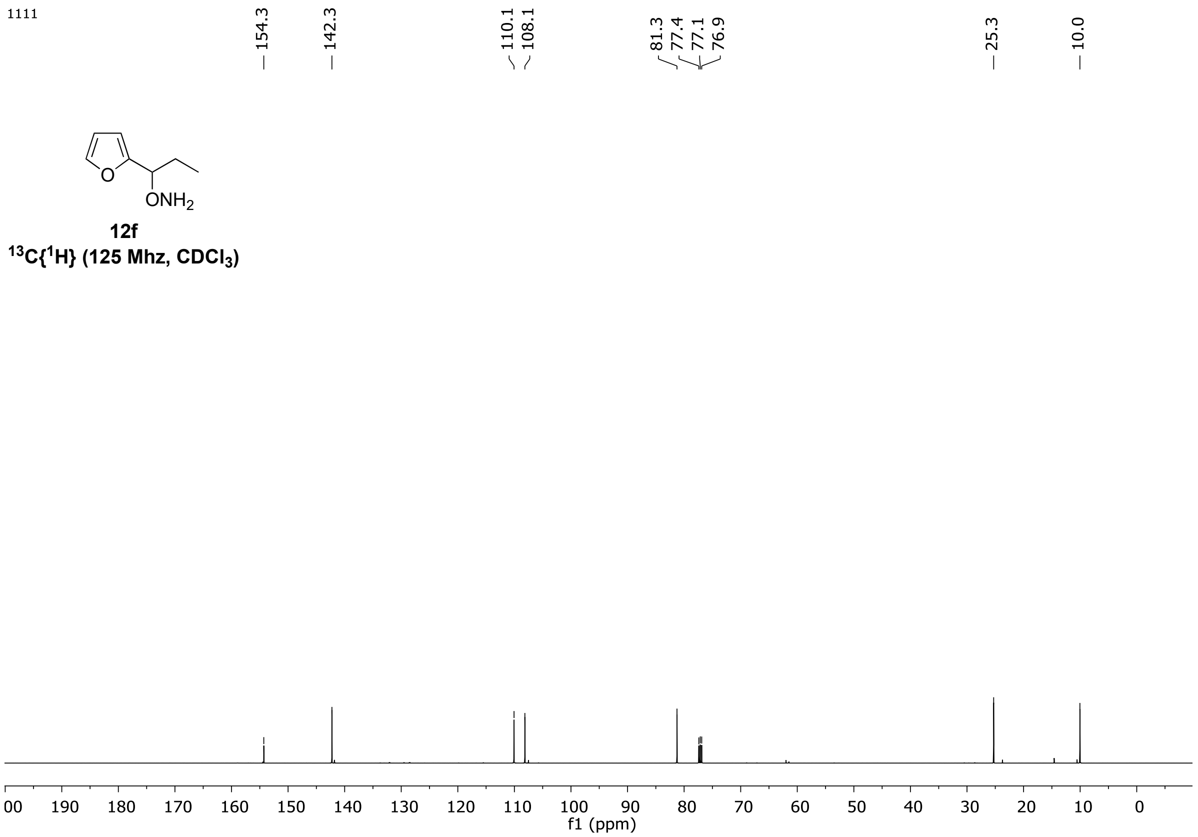

^{13}C{^{1}H} NMR Spectrum of *O*-(1-(furan-2-yl)propyl)hydroxylamine (**12f**)

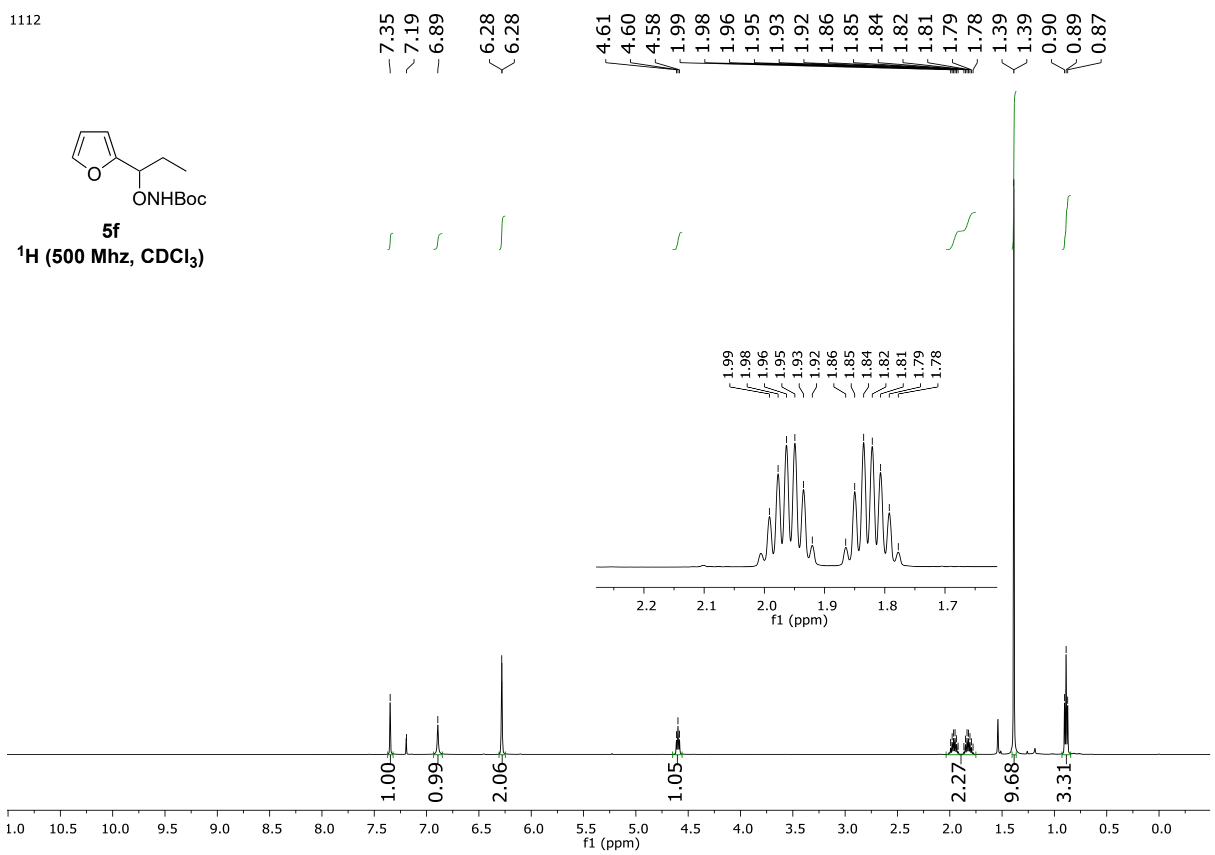

^{1}H NMR Spectrum of *tert*-butyl (1-(furan-2-yl)propoxy)carbamate (**5f**)

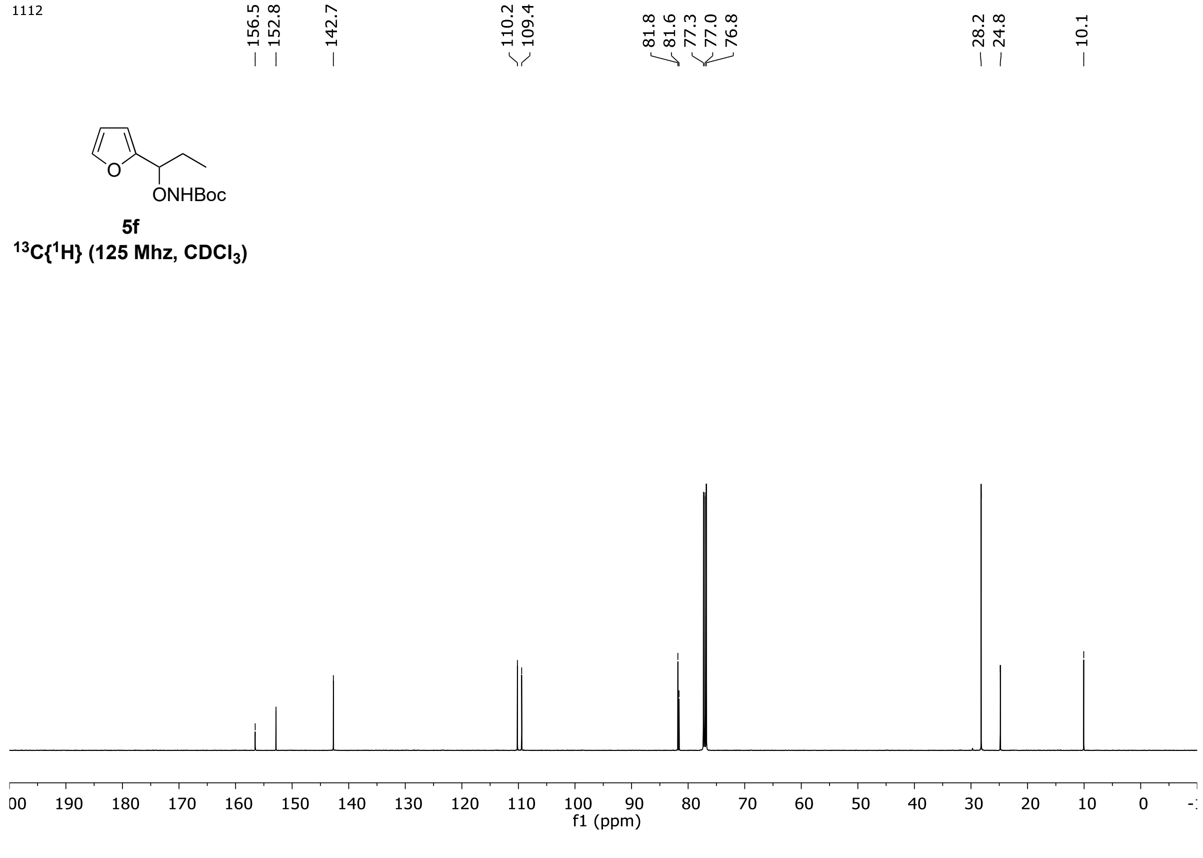

^{13}C{^{1}H} NMR Spectrum of *tert*-butyl (1-(furan-2-yl)propoxy)carbamate (**5f**)

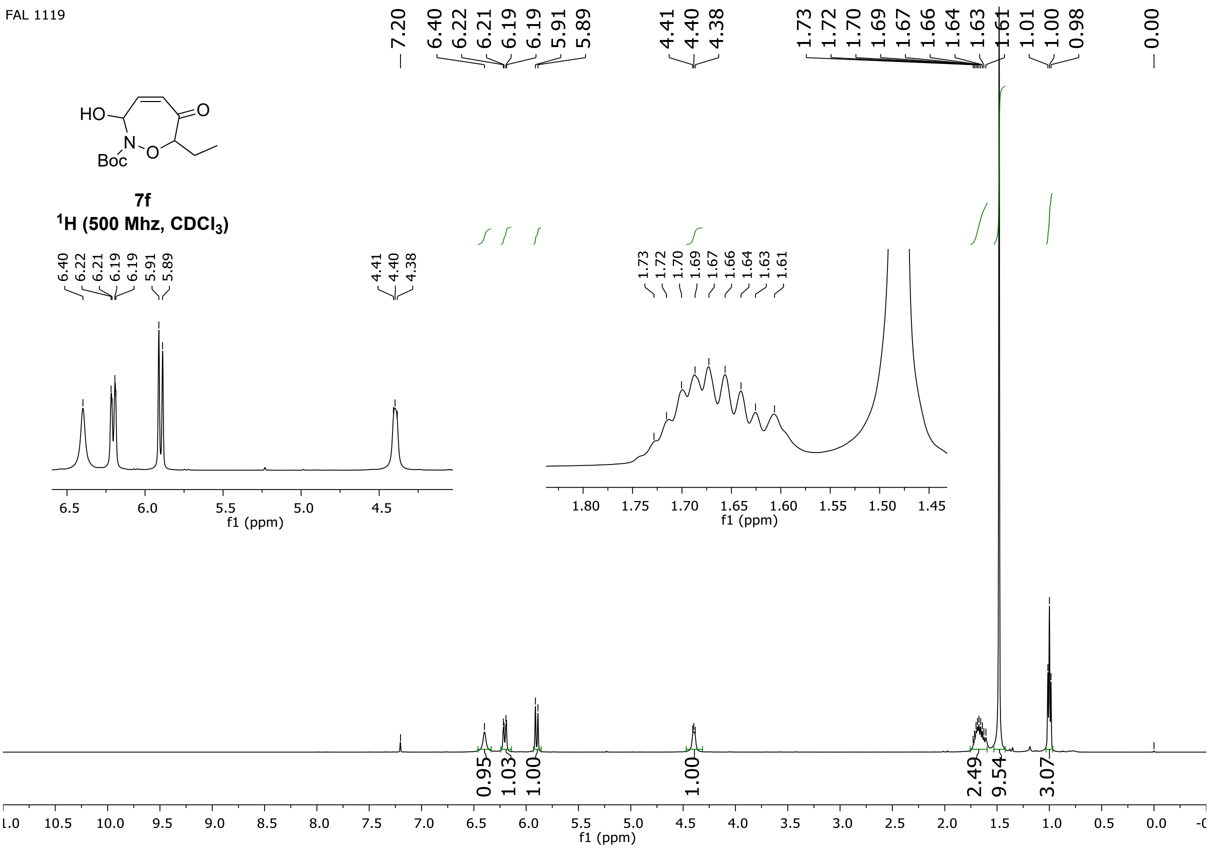

^{1}H NMR Spectrum of *tert*-butyl 7-ethyl-3-hydroxy-6-oxo-6,7-dihydro-1,2-oxazepine-2(*3H*)-carboxylate (**7f**)

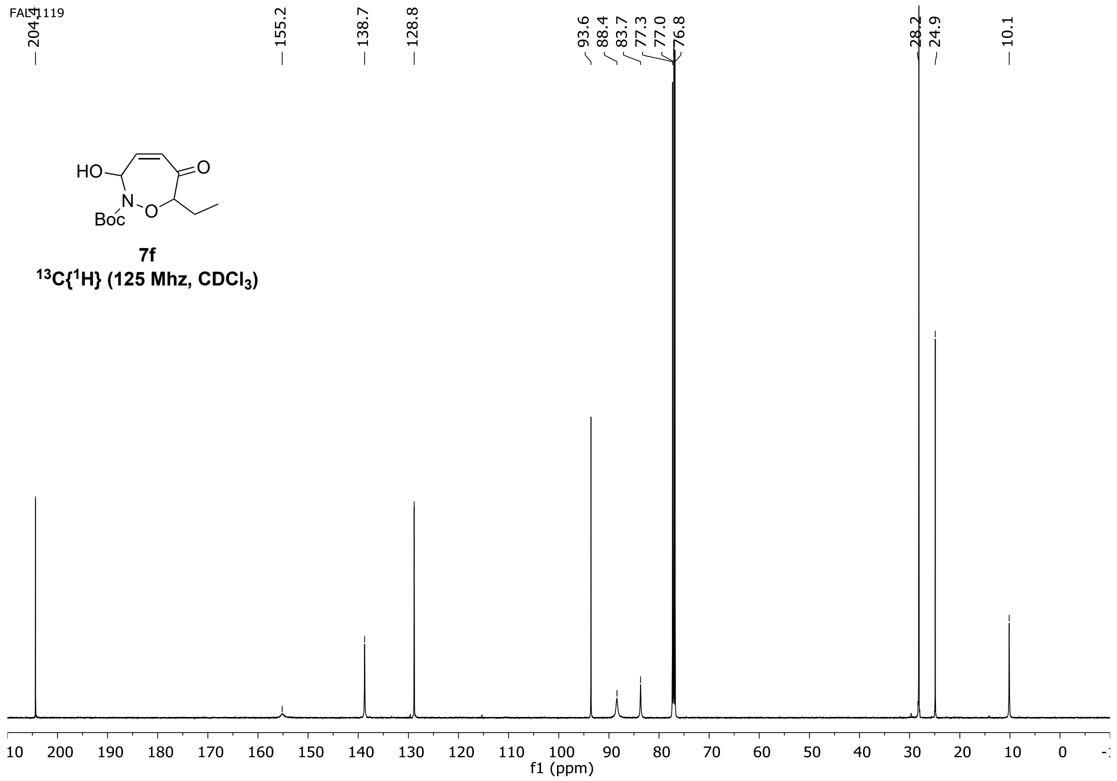

^{13}C{^{1}H} NMR Spectrum of *tert*-butyl 7-ethyl-3-hydroxy-6-oxo-6,7-dihydro-1,2-oxazepine-2(*3H*)-carboxylate (**7f**)

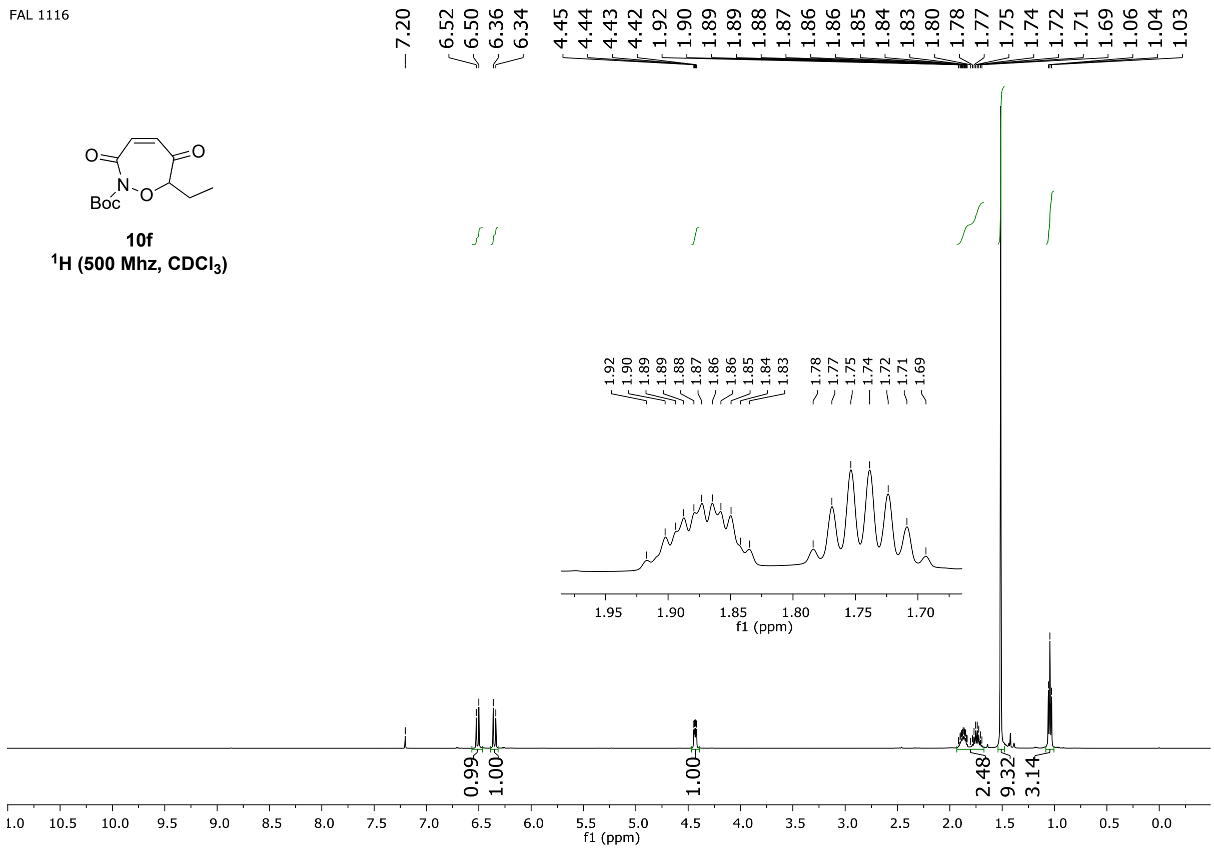

^{1}H NMR Spectrum of *tert*-butyl 7-ethyl-3,6-dioxo-6,7-dihydro-1,2-oxazepine-2(*3H*)-carboxylate (**10f**)

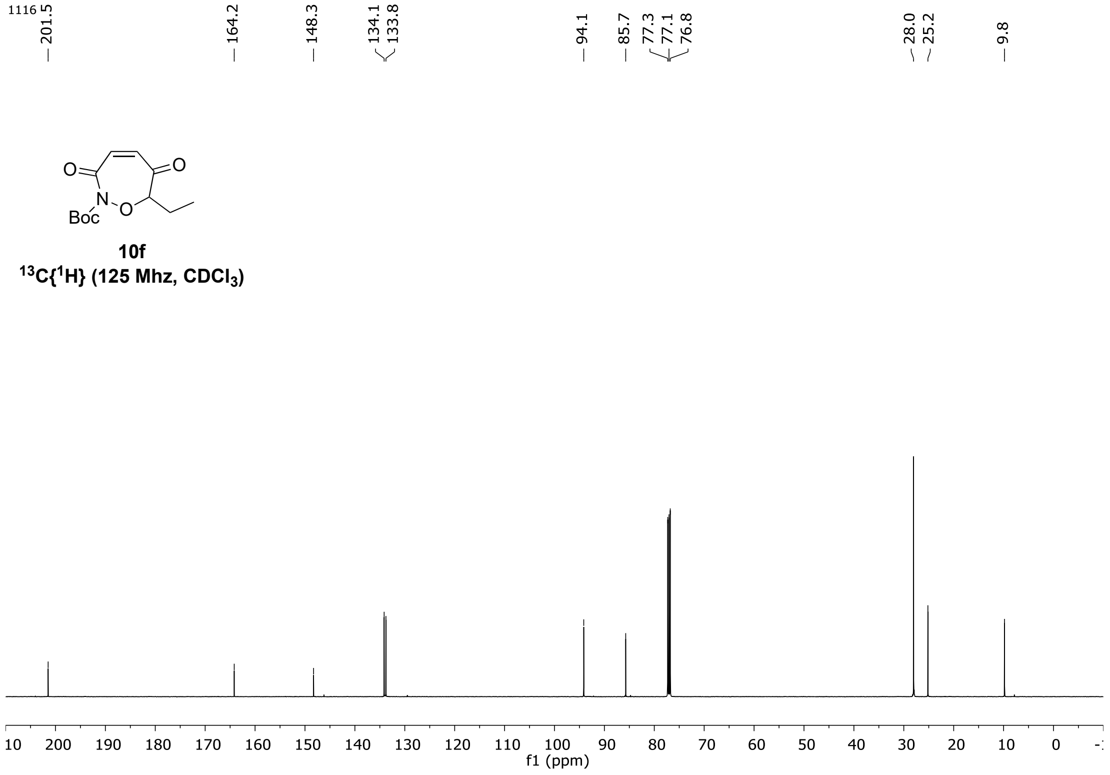

^{13}C{^{1}H} NMR Spectrum of *tert*-butyl 7-ethyl-3,6-dioxo-6,7-dihydro-1,2-oxazepine-2(*3H*)-carboxylate (**10f**)

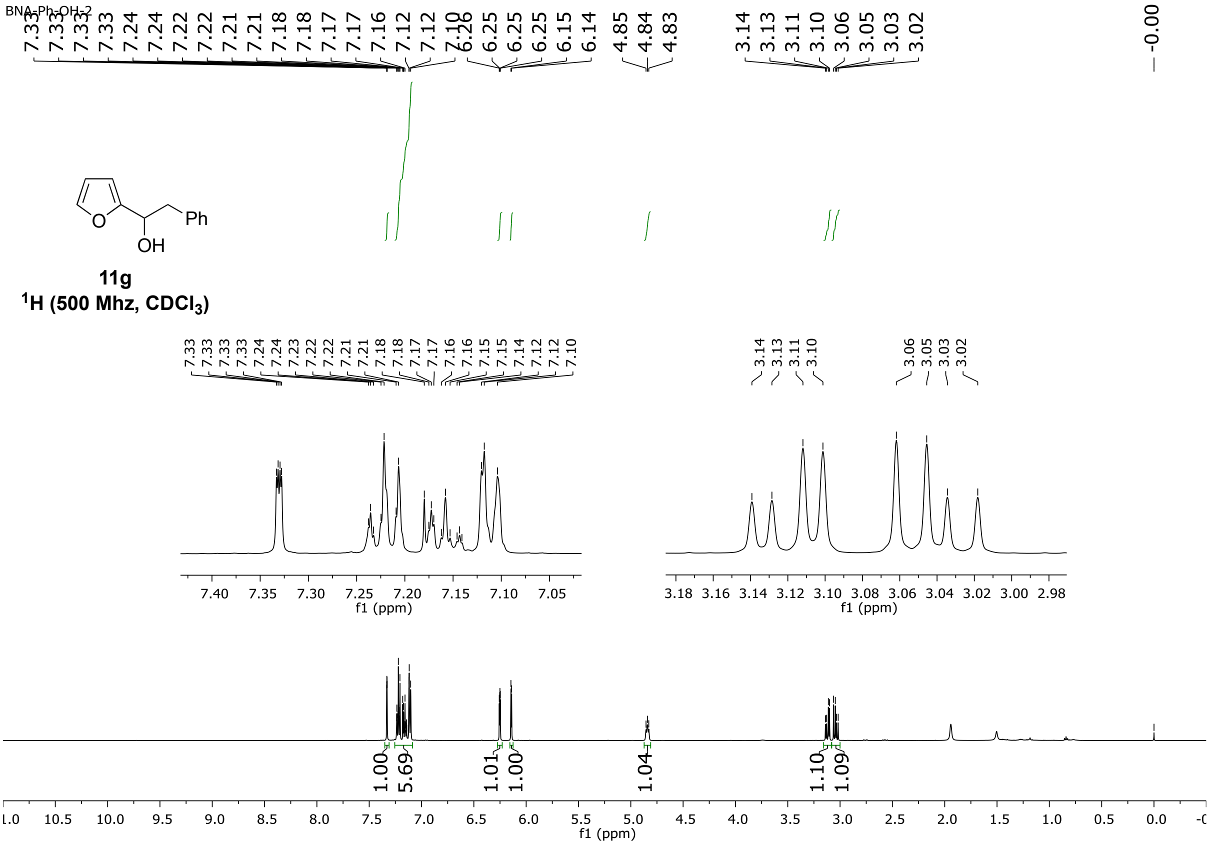

¹H NMR Spectrum of 1-(furan-2-yl)-2-phenylethanol (**11g**)

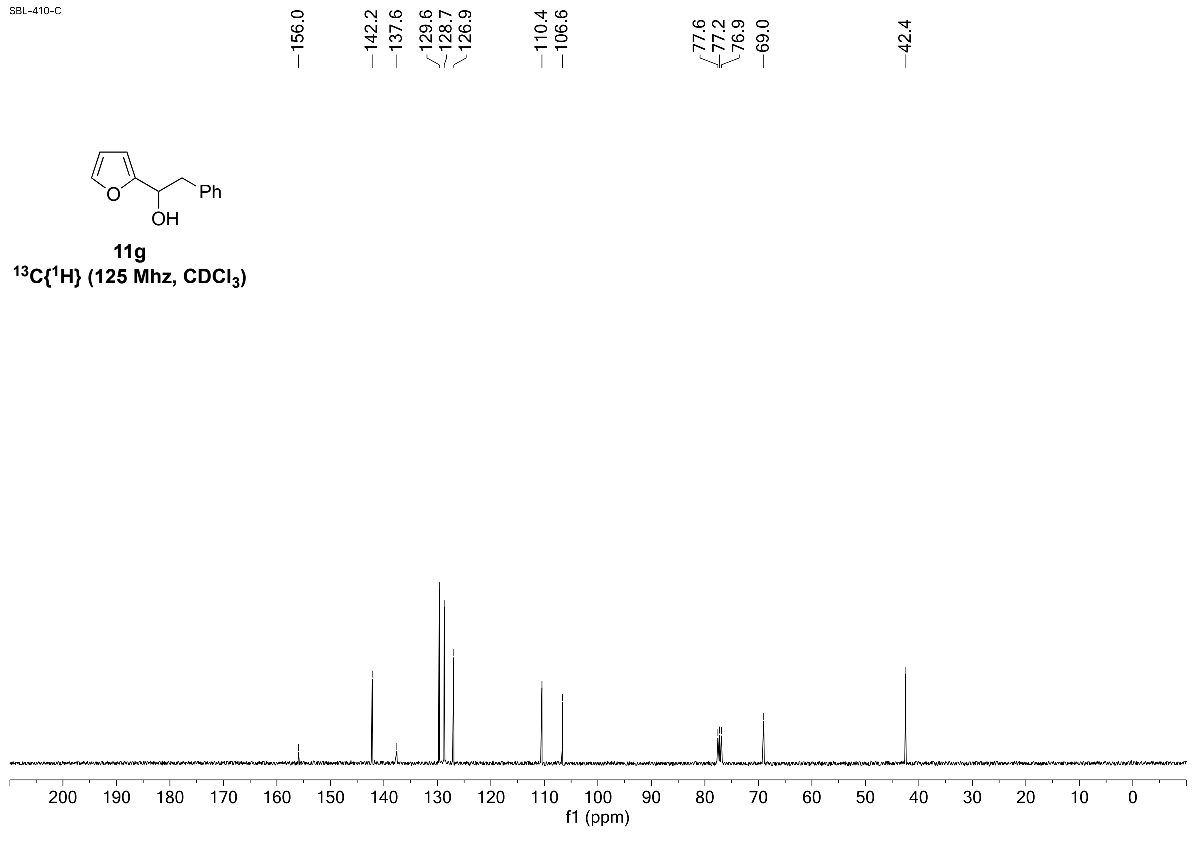

¹³C{¹H} NMR Spectrum of 1-(furan-2-yl)-2-phenylethanol (**11g**)

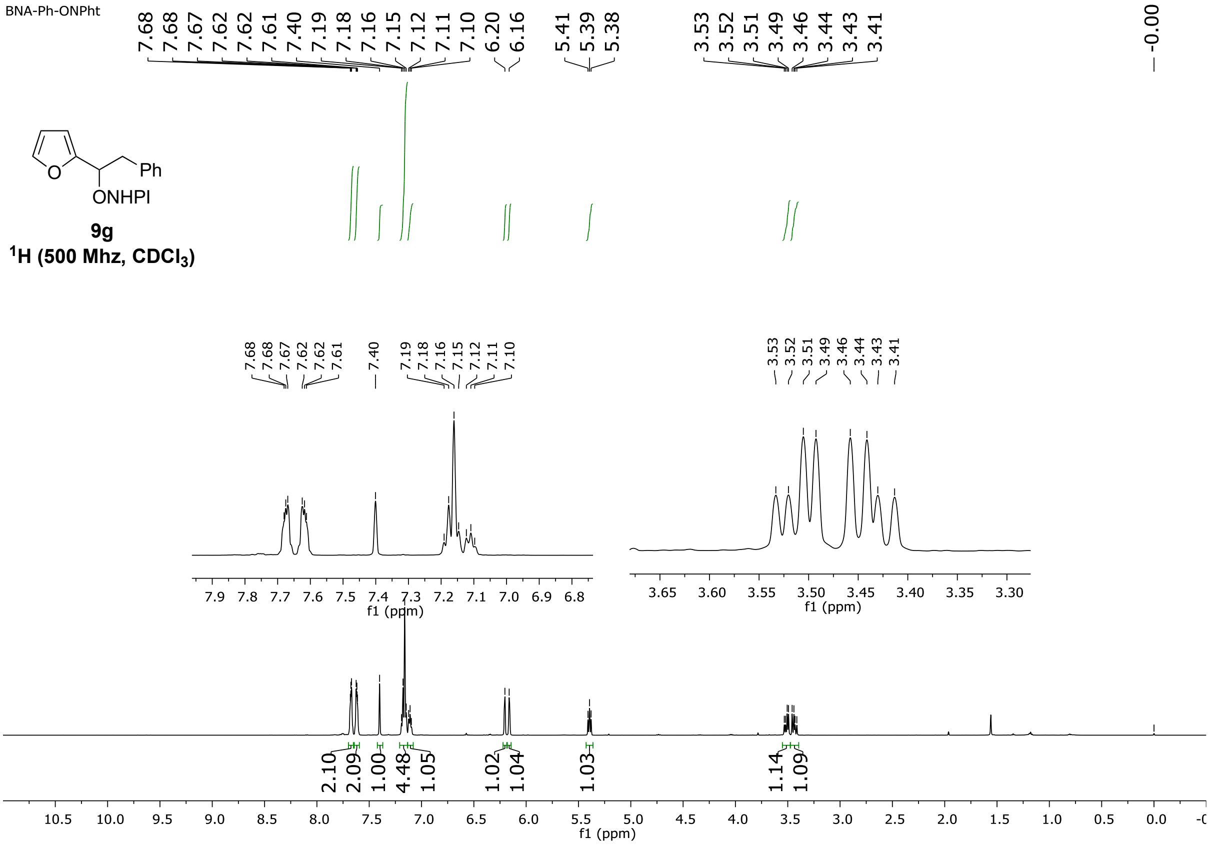

^{1}H NMR Spectrum of 2-(1-(furan-2-yl)-2-phenylethoxy)isoindoline-1,3-dione (**9g**)

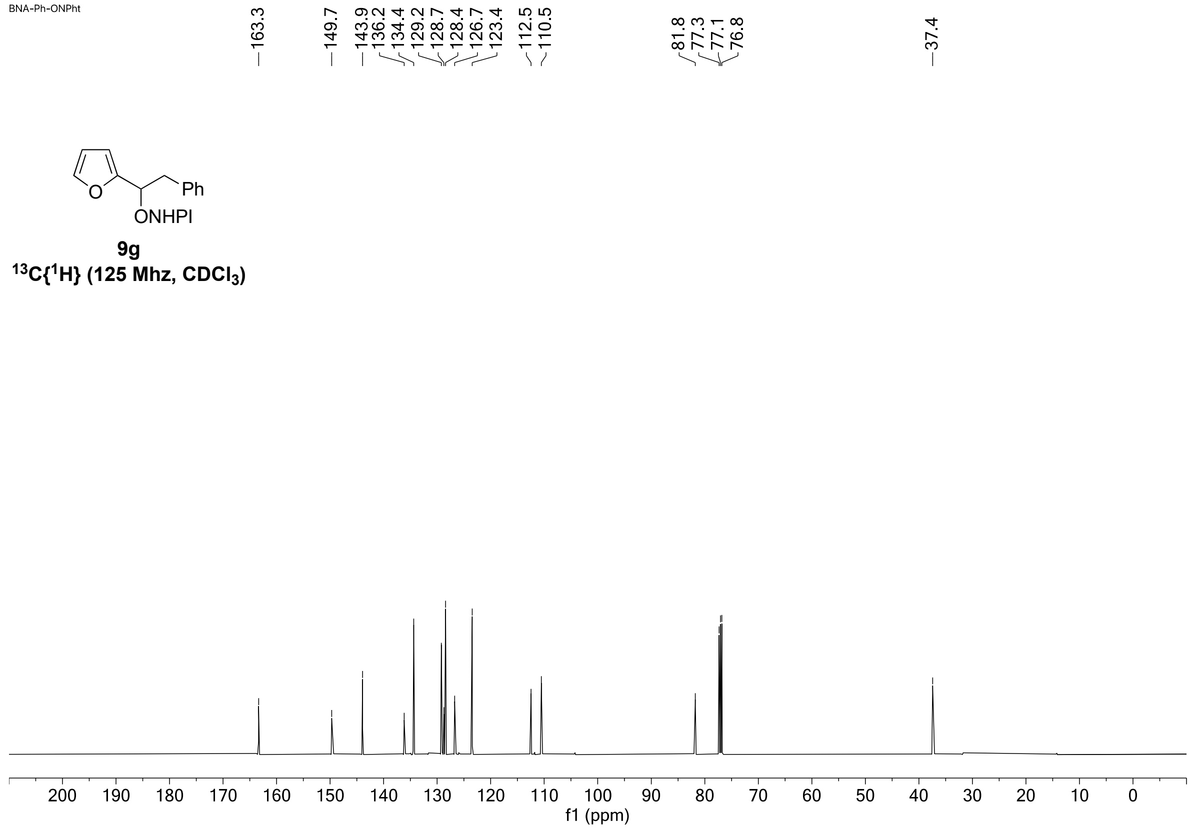

^{13}C{^{1}H} NMR Spectrum of 2-(1-(furan-2-yl)-2-phenylethoxy)isoindoline-1,3-dione (**9g**)

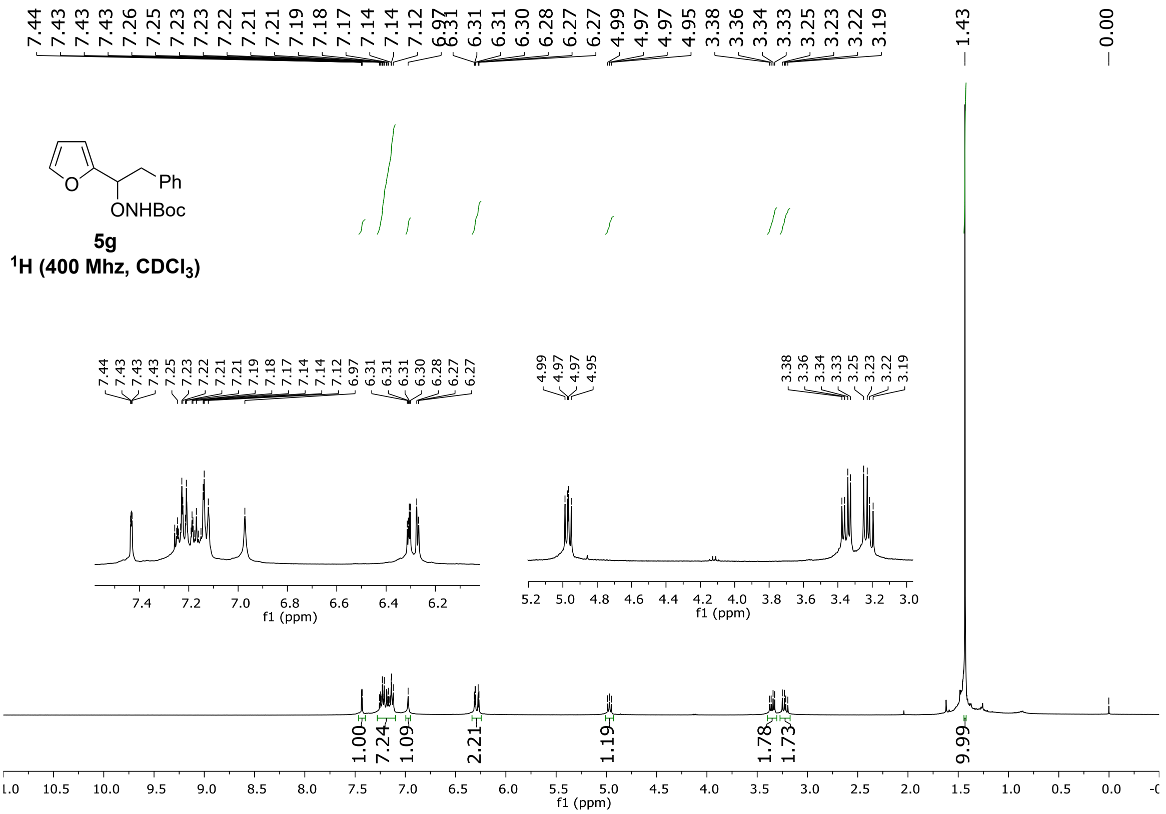

^{1}H NMR Spectrum of *tert*-butyl (1-(furan-2-yl)-2-phenylethoxy)carbamate (**5g**)

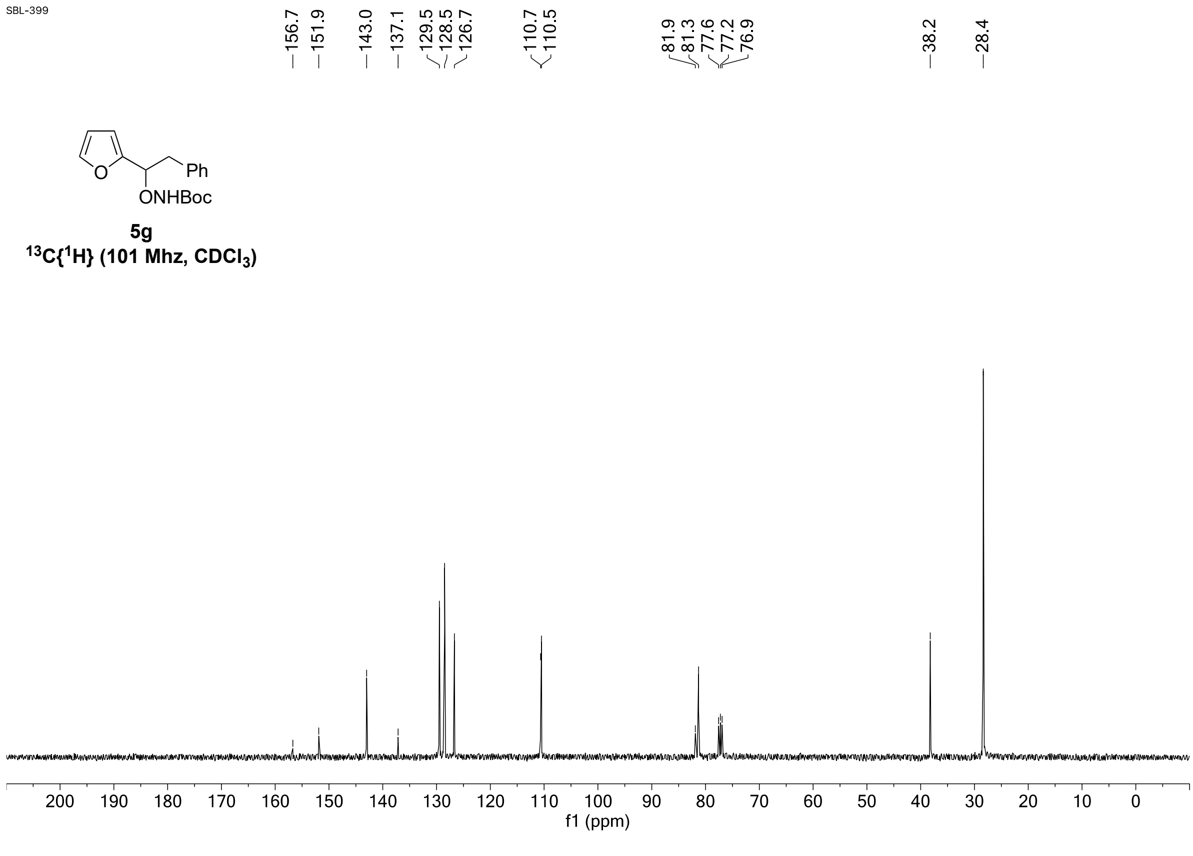

^{13}C{^{1}H} NMR Spectrum of *tert*-butyl (1-(furan-2-yl)-2-phenylethoxy)carbamate (**5g**)

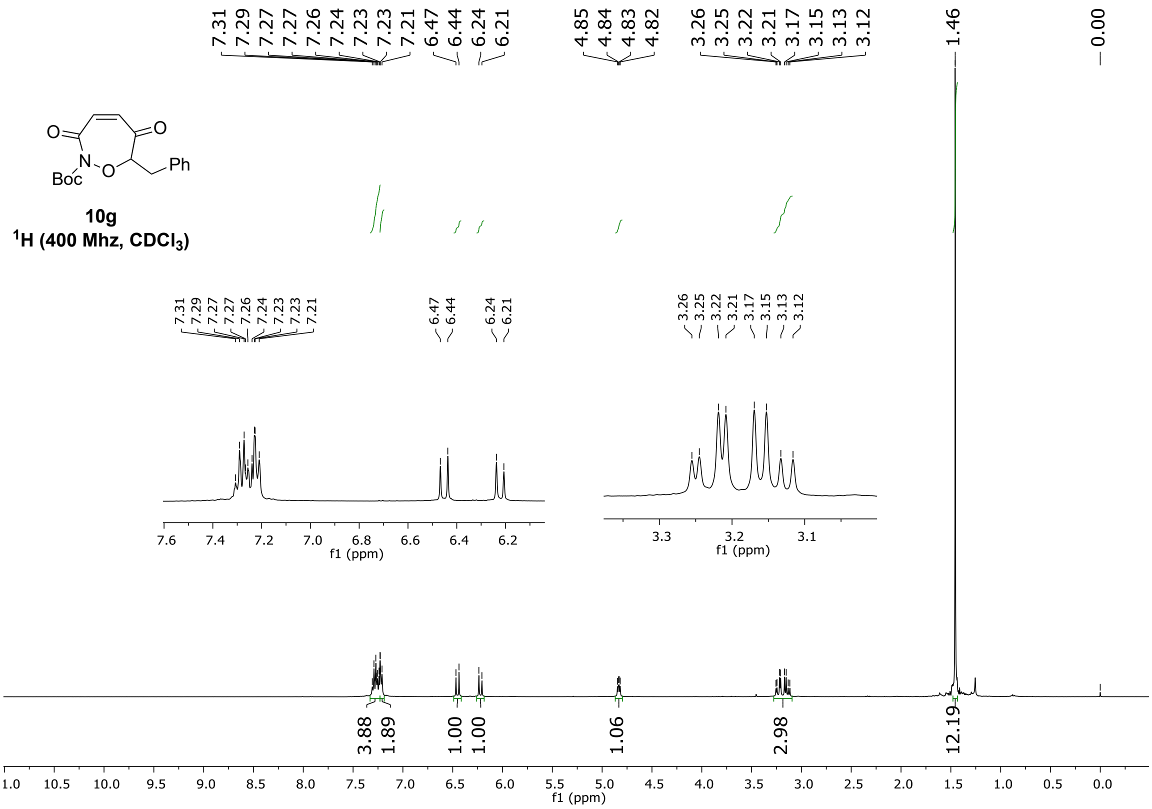

¹H NMR Spectrum of *tert*-butyl 7-benzyl-3,6-dioxo-6,7-dihydro-1,2-oxazepine-2(*3H*)-carboxylate (**10g**)

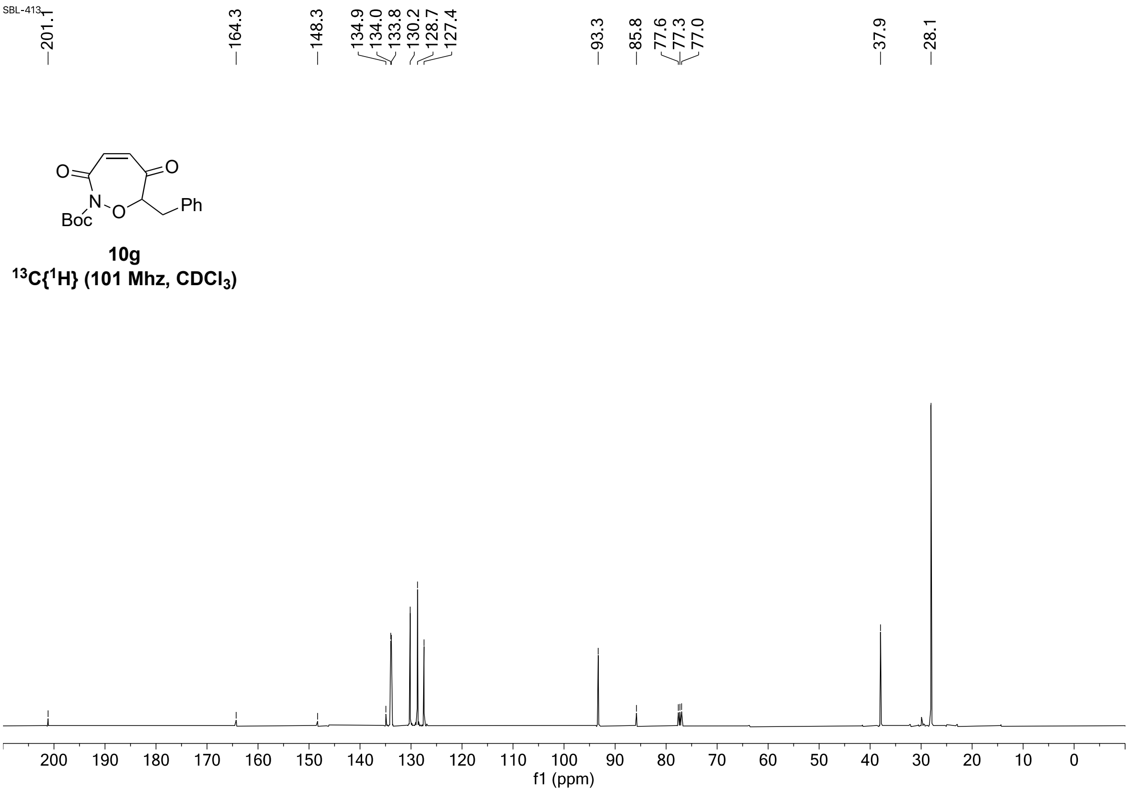

¹³C{¹H} NMR Spectrum of *tert*-butyl 7-benzyl-3,6-dioxo-6,7-dihydro-1,2-oxazepine-2(*3H*)-carboxylate (**10g**)

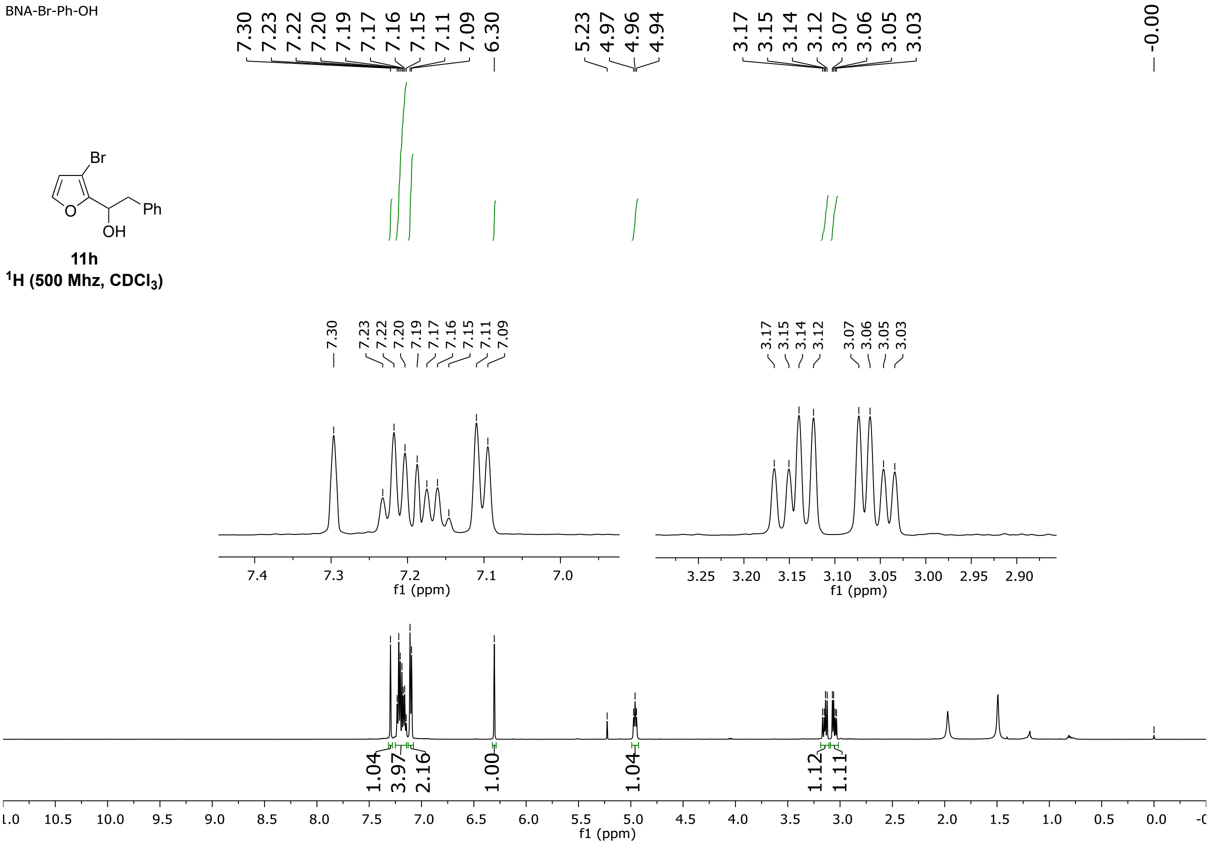

^{1}H NMR Spectrum of 1-(3-bromofuran-2-yl)-2-phenylethanol (**11h**)

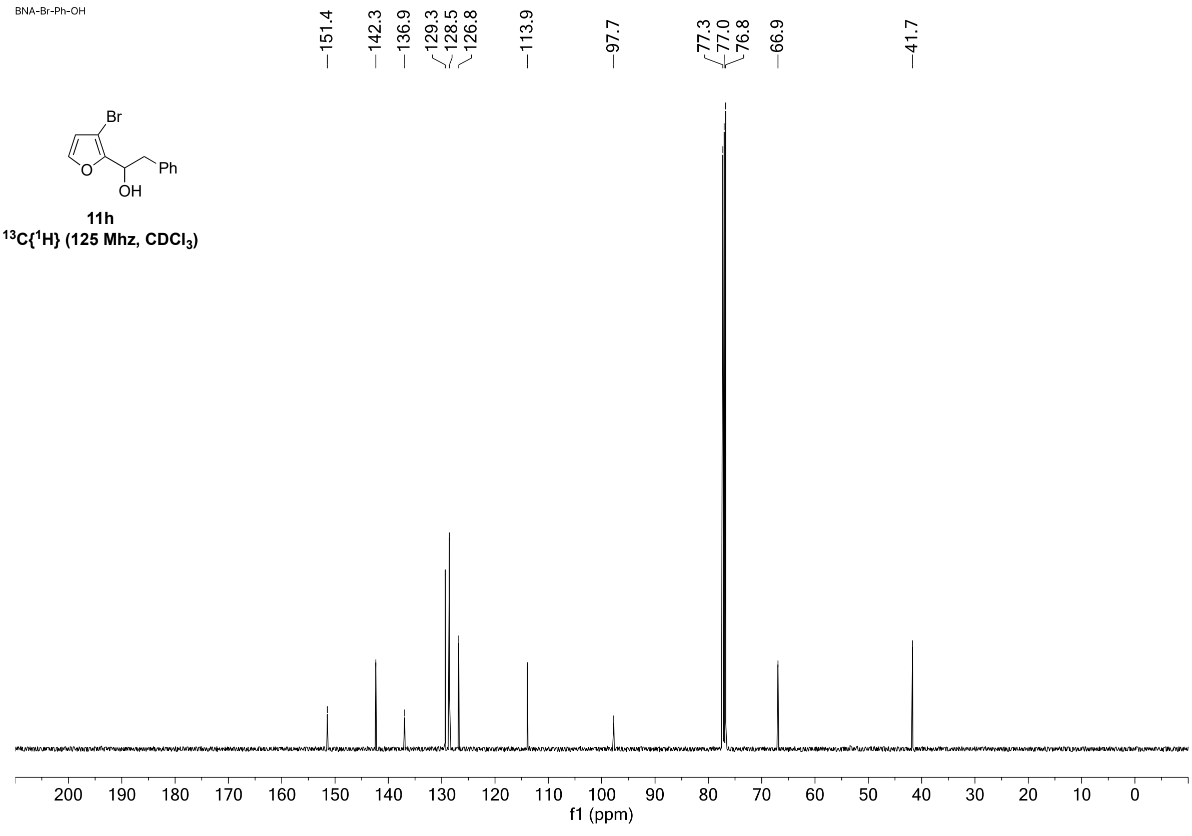

^{13}C{^{1}H} NMR Spectrum of 1-(3-bromofuran-2-yl)-2-phenylethanol (**11h**)

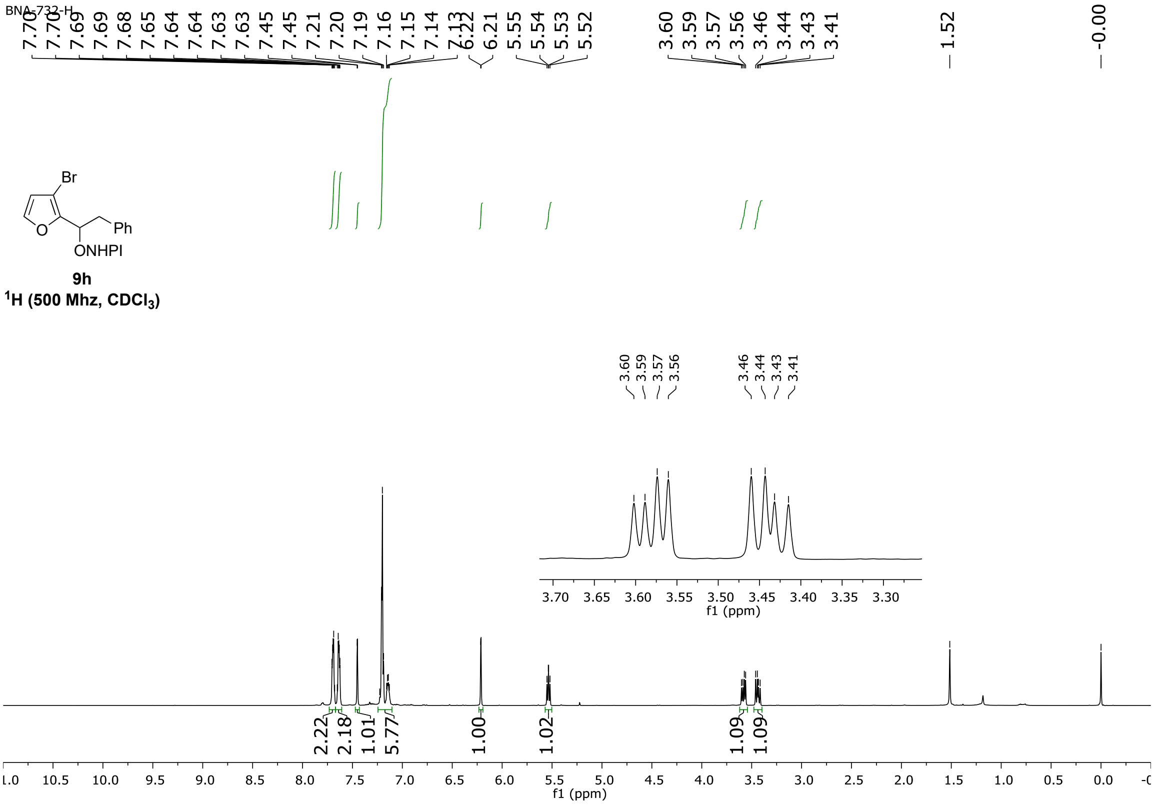

^{1}H NMR Spectrum of 2-(1-(3-bromofuran-2-yl)-2-phenylethoxy)isoindoline-1,3-dione (**9h**)

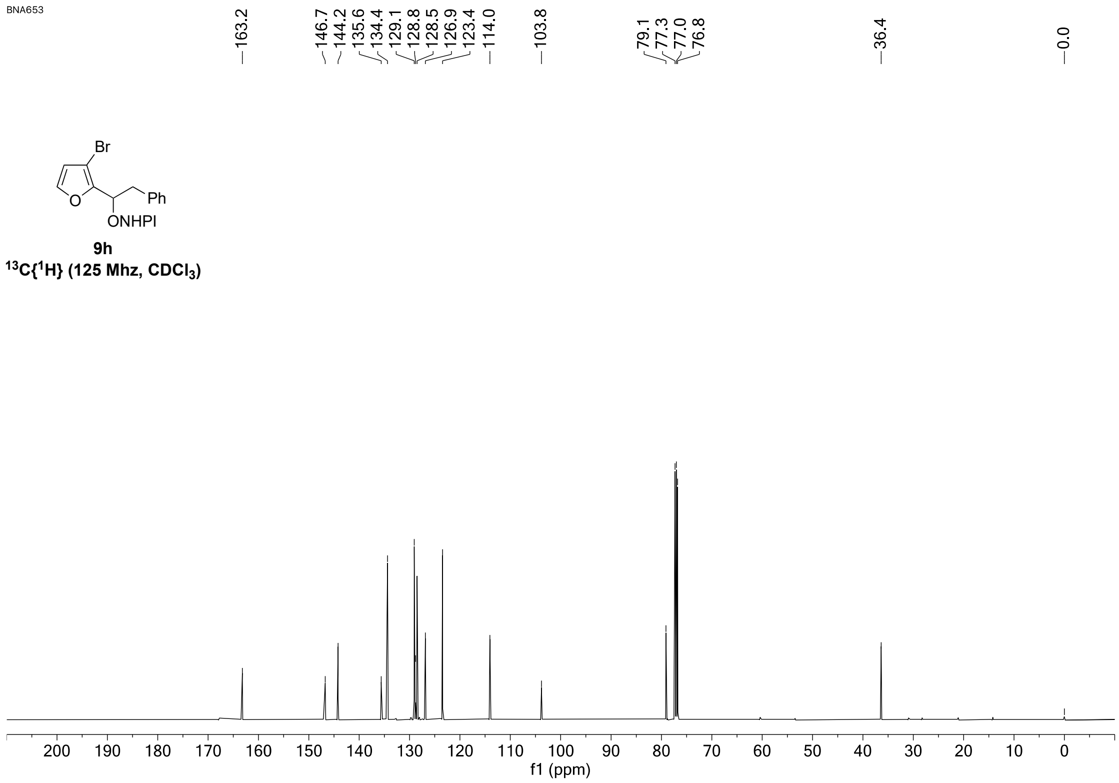

^{13}C{^{1}H} NMR Spectrum of 2-(1-(3-bromofuran-2-yl)-2-phenylethoxy)isoindoline-1,3-dione (**9h**)

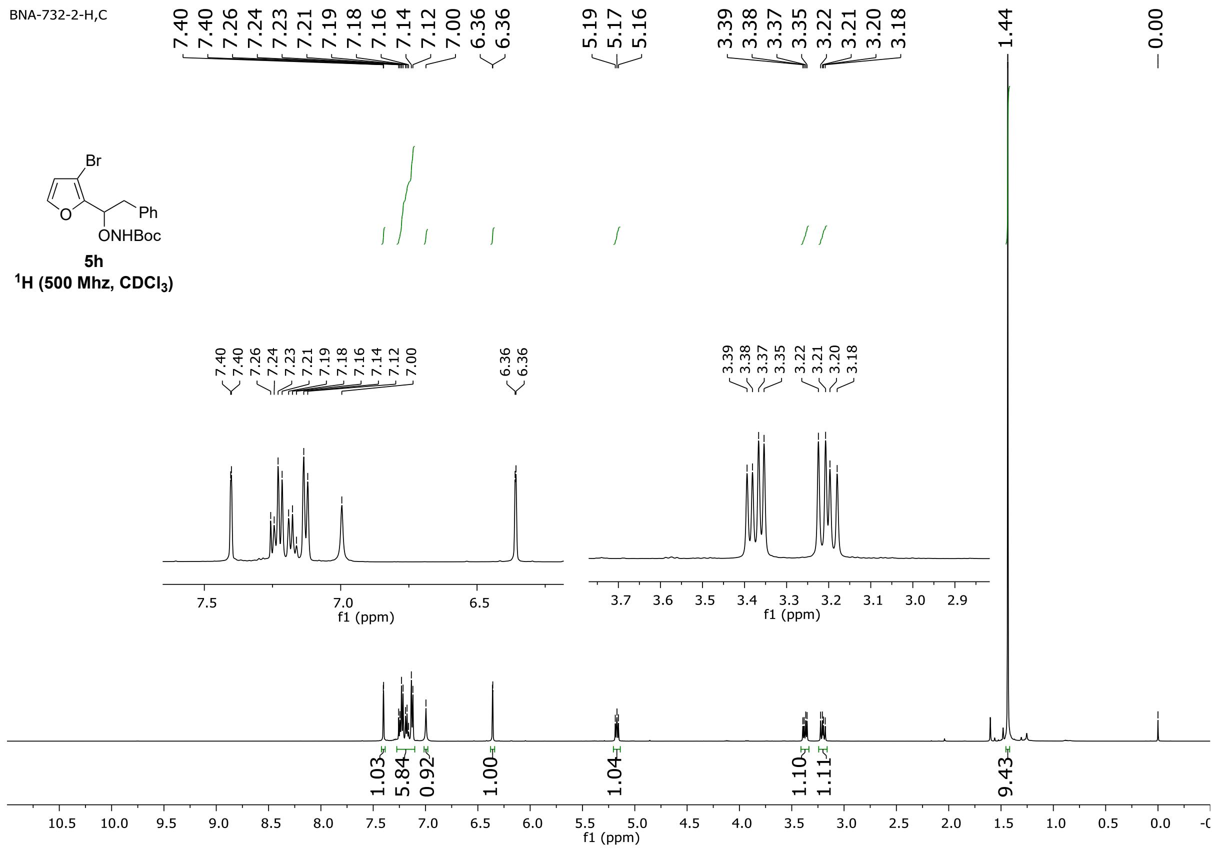

^{1}H NMR Spectrum of *tert*-butyl (1-(3-bromofuran-2-yl)-2-phenylethoxy)carbamate (**5h**)

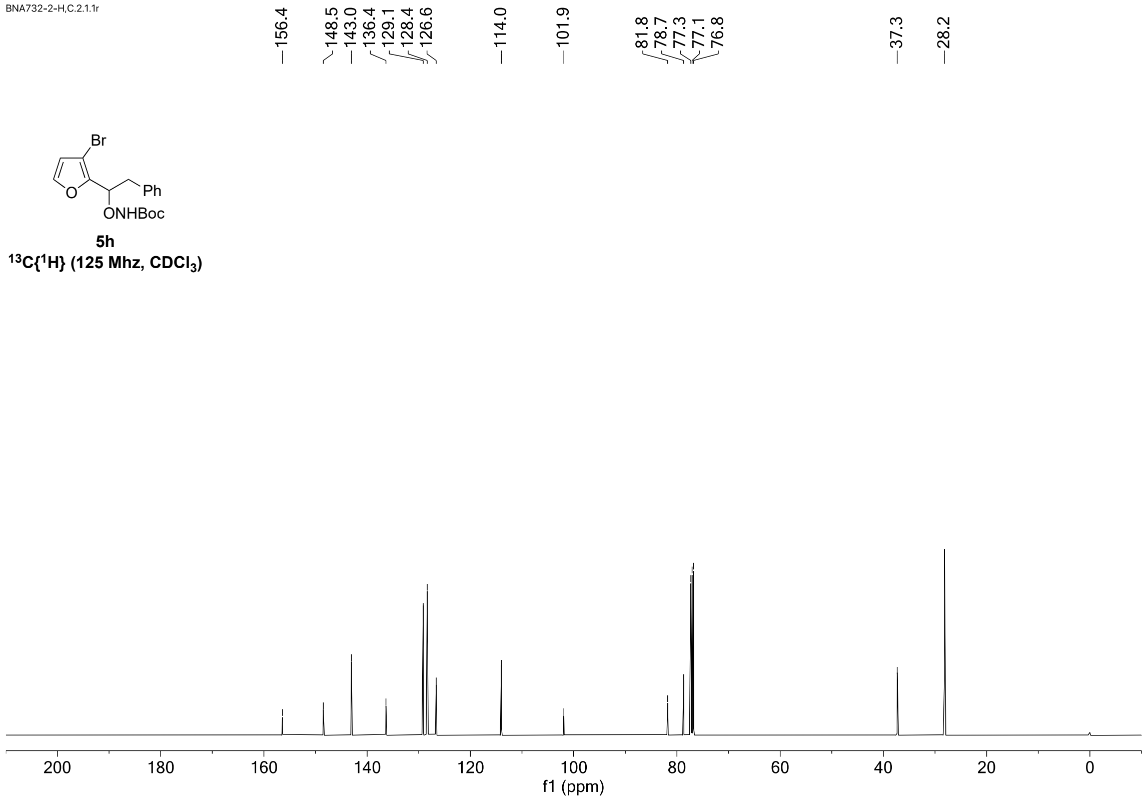

^{13}C{^{1}H} NMR Spectrum of *tert*-butyl (1-(3-bromofuran-2-yl)-2-phenylethoxy)carbamate (**5h**)

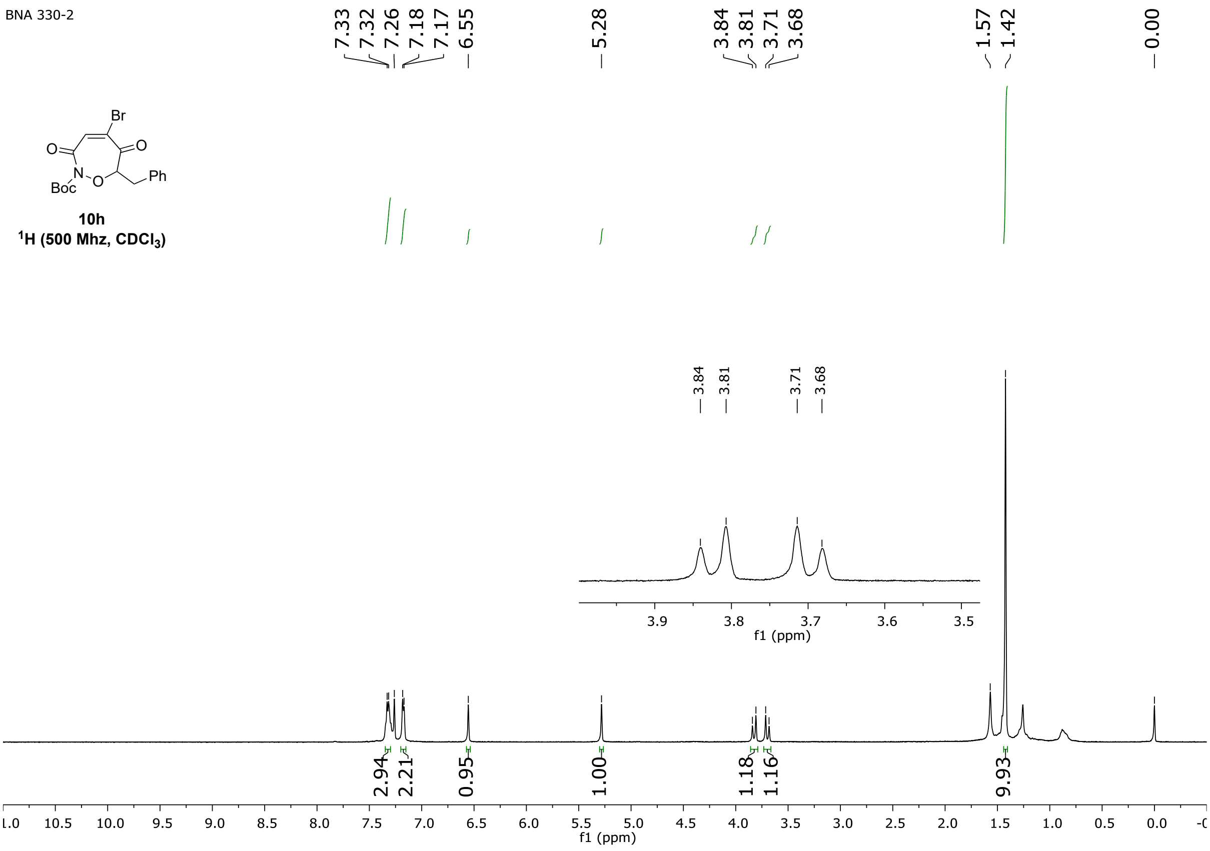

^{1}H NMR Spectrum of *tert*-butyl 7-benzyl-5-bromo-3,6-dioxo-6,7-dihydro-1,2-oxazepine-2(*3H*)-carboxylate (**10h**)

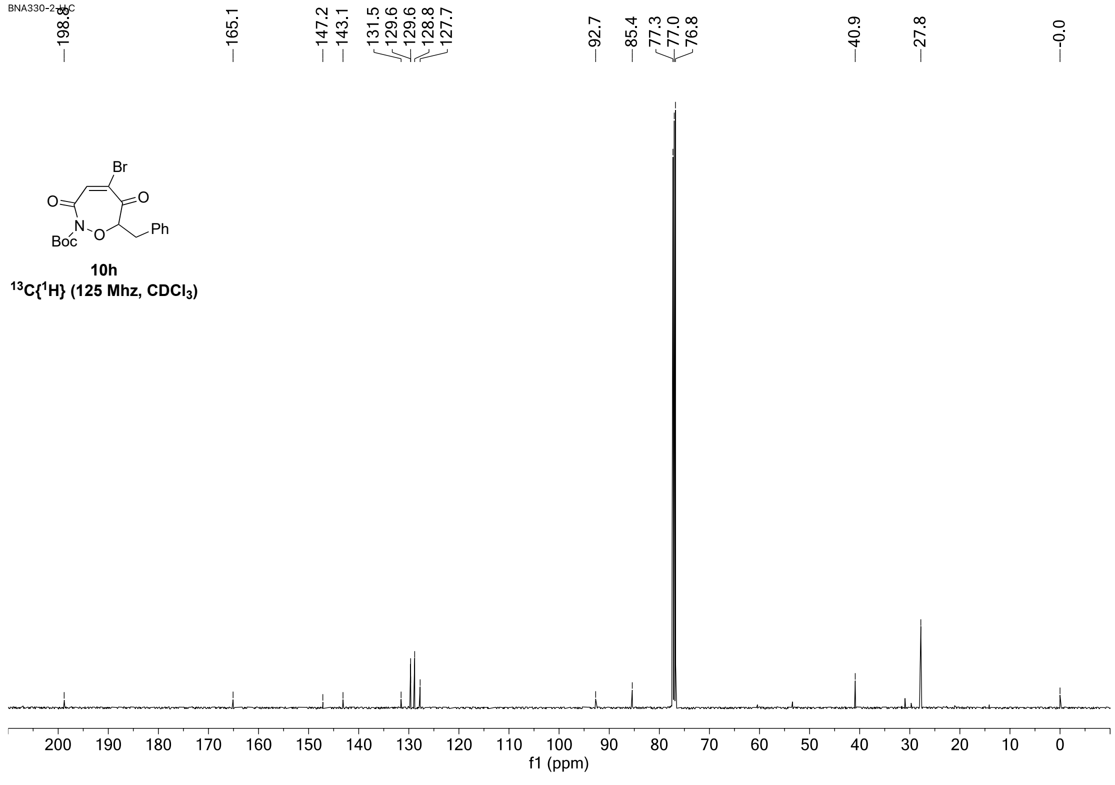

^{13}C{^{1}H} NMR Spectrum of *tert*-butyl 7-benzyl-5-bromo-3,6-dioxo-6,7-dihydro-1,2-oxazepine-2(*3H*)-carboxylate (**10h**)

6. REFERENCES

[1] Stęsik, K.; Franczyk, A.; Czapik, A.; Kownacki, I.;J. Walkowiak, J. Hydrosilylation of Carbonyl Compounds Catalyzed by Iridium(I) Complexes with (−)-Menthol-Based Phosphorus(III) Ligands. *ChemCatChem* **2023**, *15*, e202201510.

[2] Zhao, H.; John, W.; Dankwardt, J. W.; Koenig, S. G.; Singh, S. P. Directed metalation and regioselective functionalization of 3-bromofuran and related heterocycles with NaHMDS. *Tetrahedron Lett.* **2012**, *53* (2), 166-169.

[3] Yim, H., K.; Wong, H. N. C. Diastereoselective Addition Reactions of Furyl Sulfonylimine Using Chiral Boronates as Auxiliary: Application to the Enantioselective Synthesis of 2,3-Disubstituted Furyl Sulfonylamides. *J. Org. Chem.* **2004**, *69*, 2892-2895.

[4] Spek, A. L. Structure Validation in Chemical Crystallography. *Acta Crystallographica Section D* **2009**, *65* (2), 148–155. https://doi.org/10.1107/S090744490804362X.

[5] Macrae, C. F.; Edgington, P. R.; McCabe, P.; Pidcock, E.; Shields, G. P.; Taylor, R.; Towler, M.; van de Streek, J. Mercury: Visualization and Analysis of Crystal Structures. *J. Appl. Crystallogr.* **2006**, *39* (3), 453–457. https://doi.org/10.1107/S002188980600731X.

[6] Bourhis, L. J.; Dolomanov, O. V; Gildea, R. J.; Howard, J. A. K.; Puschmann, H. The Anatomy of a Comprehensive Constrained, Restrained Refinement Program for the Modern Computing Environment – Olex2 Dissected. *Acta Crystallographica Section A* **2015**, *71* (1), 59–75. https://doi.org/10.1107/S2053273314022207.

[7] Sheldrick, G. M. SHELXT – Integrated Space-Group and Crystal-Structure Determination. *Acta Crystallographica Section A* **2015**, *71* (1), 3–8. https://doi.org/10.1107/S2053273314026370.

[8] Bruker, SAINT, version 8.34A, Bruker AXS Inc., Madison, Wisconsin, USA, 2013.
